# Enantioselective PCCP Brønsted acid-catalyzed aminalization of aldehydes

**DOI:** 10.3762/bjoc.17.160

**Published:** 2021-09-16

**Authors:** Martin Kamlar, Robert Reiberger, Martin Nigríni, Ivana Císařová, Jan Veselý

**Affiliations:** 1 Department of Organic Chemistry Charles Univerzity, Hlavova 2030/8, Prague 2, 12800, Czech Republic; 2 Department of Inorganic Chemistry, Charles Univerzity, Hlavova 2030/8, Prague 2, 12800, Czech Republic

**Keywords:** aminalization, Brønsted acid, organocatalysis, PCCP, pentacarboxycyclopentadiene

## Abstract

Here we present an enantioselective aminalization of aldehydes catalyzed by Brønsted acids based on pentacarboxycyclopentadienes (PCCPs). The cyclization reaction using readily available anthranilamides as building blocks provides access to valuable 2,3-dihydroquinazolinones containing one stereogenic carbon center with good enantioselectivity (ee up to 80%) and excellent yields (up to 97%).

## Introduction

Nitrogen-containing heterocyclic compounds are commonly occurring in nature and constitute the core structures of many biologically important compounds. An important example of such heterocycles are 2,3-dihydroquinazolinones which scaffold can be found in various compounds exhibiting pharmacological properties [1–6]. Some of them are currently used to treat numerous diseases, such as the diuretic drug fenquizone used for the treatment of hypertension [7–8], or evodiamine, a stimulant used in fat reduction or inflammation [9–11]. Moreover, it was reported that both enantiomers of 2,3-dihydroquinazolinones exhibit different bioactivities [12–13]. Thus, the development of enantioselective synthetic strategies towards 2,3-dihydroquinazolinone derivatives has drawn the attention of organic chemists for a long time [14–18], even though the aminal stereocenter is sensitive to racemization [12].

**Figure 1:**
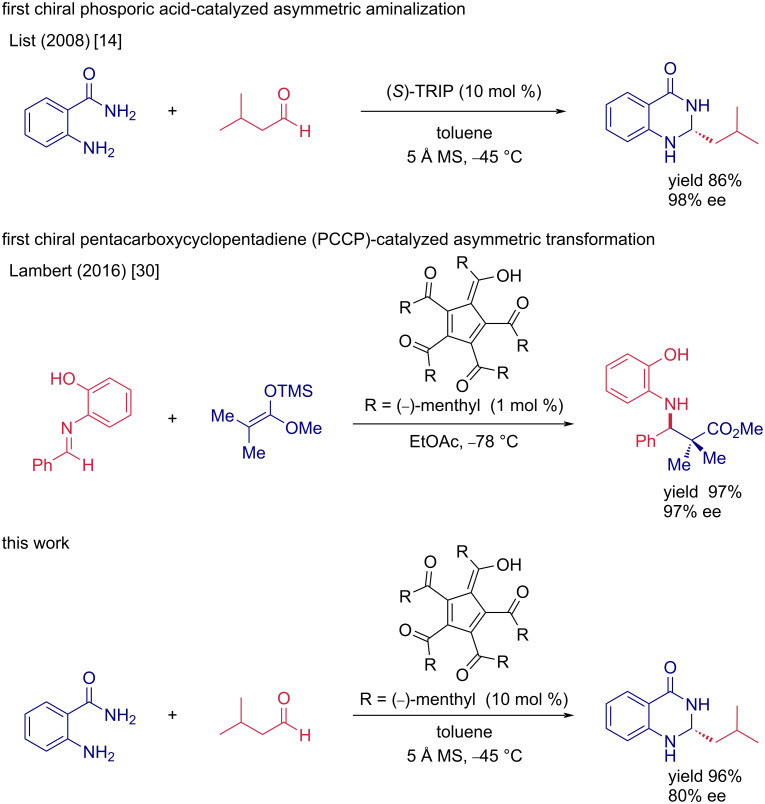
Synthetic strategies employing chiral Brønsted acid catalysis.

The well-established and straightforward approach in the asymmetric organocatalytic synthesis of molecules with this moiety uses the reaction between aldehydes and anthranilamide building blocks. The advantage of this methodology lies in the fact that both starting materials are readily available, and the enantioselectivity of such cyclization reactions can be controlled by chiral Brønsted acids. In the scope of Brønsted acid catalysis, chiral phosphoric acids (CPA) are dominating as potent catalysts in various asymmetric transformations [19–23], although the synthesis of these catalysts is expensive and laborious [24]. One of the most frequent examples of CPAs is the binaphthol (BINOL)-derived phosphoric acid class of catalysts, firstly reported by Akiyama [25] and Terada [26]. Soon after, BINOL-derived phosphoric acids were employed in the enantioselective synthesis of 2,3-dihydroquinazolinones. The initial report in this area was made by List and co-workers, using an (*S*)-TRIP derivative as the chiral catalyst (Figure 1) [14]. Soon after, Rueping et al. developed a similar methodology catalyzed by other chiral BINOL-phosphoric acids [15]. However, the reaction suffered from limited scope to aromatic aldehydes without an *ortho*-substitution; the corresponding dihydroquinazolinones were obtained in high yields and with good enantiomeric purities. In 2013, Lin and co-workers published the application of a chiral SPINOL-phosphoric acid in the asymmetric aminalization reaction [27]. Tian´s research group developed the synthesis of dihydroquinazolinones from preformed imines instead of aldehydes catalyzed by BINOL-phosphoric acid [17]. The corresponding aminals were prepared with a wide range of substitutions using aromatic, α,β-unsaturated, or aliphatic imines. Apart from chiral phosphoric acids, chiral quaternary ammonium salts were successfully employed as catalysts in asymmetric dihydroquinazolinone synthesis [18]. Regarding the above-mentioned strategies involving chiral Brønsted acids, we envisioned that chiral pentacarboxycyclopentadiene (PCCP) derivatives could be used in the enantioselective aminalization of aldehydes with anthranilamide derivatives. PCCPs were firstly reported by Otto Diels [28–29], but recently, Lambert and co-workers introduced a new generation, chiral PCCPs (Figure 1) [30]. Due to the high stability of the aromatic cyclopentadienyl anion, PCCPs exhibit a low p*K*_a_ value comparable to that of phosphoric acids. Contrary to chiral phosphoric acids, PCCPs offer less laborious and inexpensive preparation protocols [31–32], which makes them an interesting alternative for chiral Brønsted acid-catalyzed transformations [30–35].

## Results and Discussion

Herein, we describe our findings regarding the aminalization of aldehydes using PCCP catalysis. Our investigation commenced with the screening of the reaction between anthranilamide (**1a**) and isovaleraldehyde (**2a**) in the presence of 10 mol % of catalyst **II** (Table 1). First, we turned our attention to the solvent and temperature effect concerning the yield and the enantioselectivity of the aminalization reaction. While most solvents tested showed to be effective at room temperature, the enantiomeric purity of the corresponding aminal **3a** was low in all cases (Table 1, entries 1–5). On the other hand, the yield of **3a** was satisfactory in all reactions. In particular, when the reaction between **1a** and **2a** was performed in toluene, the isolated yield of **3a** was almost quantitative (97%, entry 1 in Table 1). In our pursuit of better enantioselectivity, we continued with the reaction proceeded in toluene at lower temperatures. We found a temperature of −45 °C as optimal for the enantiocontrol of the model reaction, affording the product **3a** in 90% yield with an enantiomeric purity of 66% ee (Table 1, entry 7). Additionally, the effect of molecular sieves on the course of the reaction was investigated and the obtained results demonstrated that molecular sieves dramatically improved the enantioselectivity (Table 1, entries 9–11). In particular, when the aminalization reaction between **1a** and **2a** was carried out in the presence of 5 Å molecular sieves, the corresponding product **3a** was delivered in high yield (96%) and with enantiomeric purity 80% ee (Table 1, entry 11). In addition, the effect of the catalyst loading on the course of the reaction was examined. Our data clearly show that reducing the catalyst loading of **II** caused a significant decrease in the enantioselectivity (Table 1, entries 12 and 13). It is worth mentioning that no differences in the enantioselectivity were observed after a prolonged exposure of compound **3a** to the chiral PCCP catalyst **II** indicating a relatively high stability of the new chiral carbon center in product **3a**.

**Table 1:** Optimization of reaction conditions for the aminalization reaction between **1a** and **2a**.

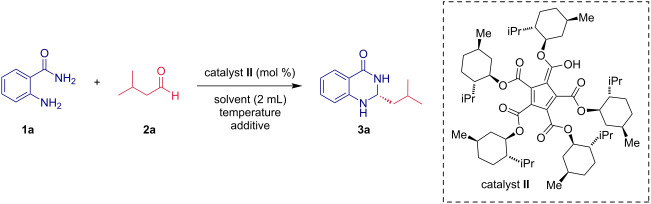							

entry	solvent	temperature [°C]	cat. loading [mol %]	additive	time [h]	yield [%]^a^	ee [%]^b^

1	toluene	25	10	–	0.5	97	50
2	THF	25	10	–	1	72	50
3	MTBE	25	10	–	1	50	40
4	DCM	25	10	–	1	93	45
5	EtOAc	25	10	–	1	86	44
6	toluene	0	10	–	12	96	58
7	toluene	−45	10	–	20	90	66
8	toluene	−65	10	–	48	65	60
9	toluene	−45	10	3 Å MS	20	81	71
10	toluene	−45	10	4 Å MS	21	73	73
11	toluene	−45	10	5 Å MS	21	96	80
12	toluene	−45	5	5 Å MS	18	91	74
13	toluene	−45	2	5 Å MS	16	86	74

^a^Isolated yield; ^b^determined by chiral HPLC.

Next, a small set of functionalized derivatives of cyclopentadienes as organocatalysts was surveyed in the model reaction (Table 2). Apart from model catalyst **II**, equipped with five (−)-menthol units, also the sterically less demanding amide-type catalyst **III** and the thiourea derivative **IV** were tested (Table 2). First, the diamide-type catalyst **III** was examined (Table 2, entry 4). Although complete conversion of **1a** and **2a** was achieved after a significantly prolonged time (7 days), the aminal **3a** was isolated in a good yield of 60%. Unfortunately, the reaction proceeded nearly in a racemic fashion. An inefficient catalyst showed up to be the PCCP catalyst derivatized with thiourea functional units (**IV**); a formation of **3a** was not observed even after prolonged reaction time (Table 2, entry 5). It is also worth mentioning that the non-catalyzed reaction did not deliver the corresponding product **3a** even after 40 hours (Table 2, entry 1). Based on the results summarized in Table 2, the chiral PCCP catalyst **II** was selected as the optimal catalyst.

**Table 2:** Catalyst screening of the aminalization reaction between **1a** and **2a**.

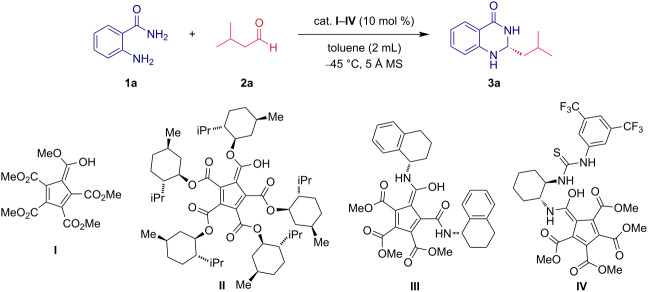				

entry	catalyst	time [h]	yield [%]^a^	ee [%]^b^

1	–	40	n.d.	n.d.
2	**I**	16	95	0
3	**II**	21	96	80
4	**III**	168	60	2
5	**IV**	168	n.d.	n.d.

^a^Isolated yield; ^b^determined by chiral HPLC.

**Scheme 1:**
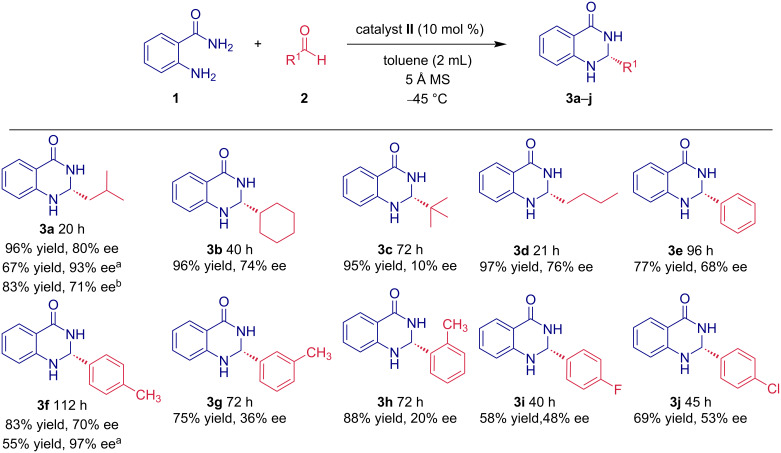
The substrate scope of the aminalization reaction for different aldehydes. ^a^After recrystallization; ^b^reaction run at 1 mmol scale.

With the optimized reaction conditions in our hands, we continued investigating the scope of the reaction. First, we focused on the reactivity of anthranilamide (**1a**) with various aldehydes **2a–j** (Scheme 1). Generally, aliphatic aldehydes delivered the cyclic aminals **3a–d** in excellent yields between 95–97% and enantiomeric purities between 74–80% ee. However, the sterically demanding pivalaldehyde (**2c**) needed a prolonged reaction time to reach the complete conversion. In addition, a significant drop in the enantioselectivity (10% ee) of **3c** was observed. Also, benzaldehyde derivatives were successfully tested in the aminalization reaction. However, a decrease in reactivity and enantioselectivity was observed when compared to aliphatic aldehydes. The corresponding products **3e–j** were isolated in lower yields (58–83%) with enantiomeric purities ranging from 20 to 70% ee. For example, when benzaldehydes substituted with fluorine or chlorine in the *para*-position were employed in catalytic reaction with anthranilamide (**1a**), the corresponding derivatives **3i**,**j** were isolated in 58 and 69% yield, respectively. The rates of enantioselectivity for both reactions were lower and averaged only around 50%. In addition, the role of an electron-donating methyl group on the aromatic ring was investigated. When *p*-tolualdehyde (**1f**) was used in the cyclization reaction with anthranilamide (**1a**), the corresponding aminal **3f** was obtained in high yield (83%) and with good enantiomeric excess of 70% ee. On the other hand, when *m*- or *o*-tolualdehyde were employed in aminalization reaction, a significant drop in the enantioselectivity was observed. Aminals **3g** and **3h** were obtained with 36 and 20% ee, respectively. We have also tested the reaction between anthranilamide (**1a**) and isovaleraldehyde (**2a**) in 1 mmol scale. The obtained results suggested that the reaction proceeded with slightly lower efficiency giving product **3a** in 83% yield and 71% ee. On the other hand, we found that the desired product of aminalization reaction could be readily obtained in higher enantiomeric purity after crystallization from ethyl acetate. This was demonstrated for products **3a** and **3f**, that were obtained in enantiomeric purities of 93% and 97% ee, respectively (Scheme 1).

**Scheme 2:**
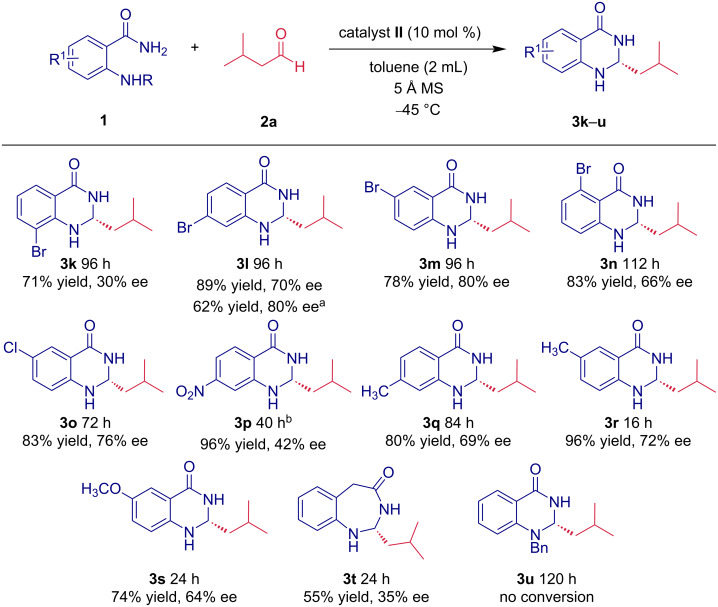
The substrate scope of the intermolecular aminalization reaction for anthranilamide derivatives. ^a^After recrystallization; ^b^THF used as a reaction solvent.

Next, we turned our attention to the substitution of anthranilamide (Scheme 2). First, the effect of bromine as a slightly electron-withdrawing substituent on the aromatic ring was investigated. The position of bromine on the aromatic ring had a dramatic effect on the enantiomeric purity of the formed products **3k–n**. When a bromine substituent is introduced in the “3” position of anthranilamide, the enantiomeric enrichment of aminal **3k** reached only 30% ee. In contrast, substitution with bromine either in position “4” and “5” led to a formation of products **3l** and **3m** with enantiomeric purities of 70% ee and 80% ee, respectively. Finally, reaction with anthranilamide substituted with bromine in position “6” led to corresponding aminal **3n** with an enantiomeric excess of 66% ee. We also increased the enantiomeric purity of **3l** from 70% to 80% ee after crystallization from ethyl acetate. When anthranilamide substituted with a chlorine in the “5” position was used, the enantioselectivity of the reaction reached a value of 76% ee*,* and the yield of the corresponding aminal **3o** exceeded 80%. Next, the effect of a strongly electron-withdrawing nitro group present on anthranilamide moiety was investigated. The reaction carried out in toluene did not reach a complete conversion even after a prolonged reaction time. When more polar THF was used as the solvent, the corresponding product **3p** was obtained after 40 hours in an excellent yield of 96%; however, the enantiomeric purity of **3p** was only 42% ee. Anthranilamides containing electron-donating methyl and methoxy groups were also well-tolerated in the aminalization reaction. For example, reaction with anthranilamide bearing a methyl group in the “4” position delivered product **3q** in good yield (80%) and enantiopurity (69% ee). A higher yield (96%) and enantiopurity (72% ee) was reached with anthranilamide **1r**, having a methyl group in the position “5”. To further broaden the scope of the aminalization reaction, we prepared 2-(2-aminophenyl)acetamide (**1t**) and tested it in the reaction with isovaleraldehyde (**2a**) to access benzodiazepinone derivatives. The reaction proceeded smoothly with complete conversion within 24 hours, yielding the desired benzodiazepinone derivative **3t** in 55%. However, the enantiomeric purity dropped significantly to 35% ee. Additionally, we tested the influence of substitution of the aromatic amine and prepared the benzyl-protected anthranilamide **1u**. Unfortunately, the reaction between **1u** and isovaleraldehyde (**2a**) did not deliver the corresponding product **3u** even after a prolonged reaction time.

**Figure 2:**
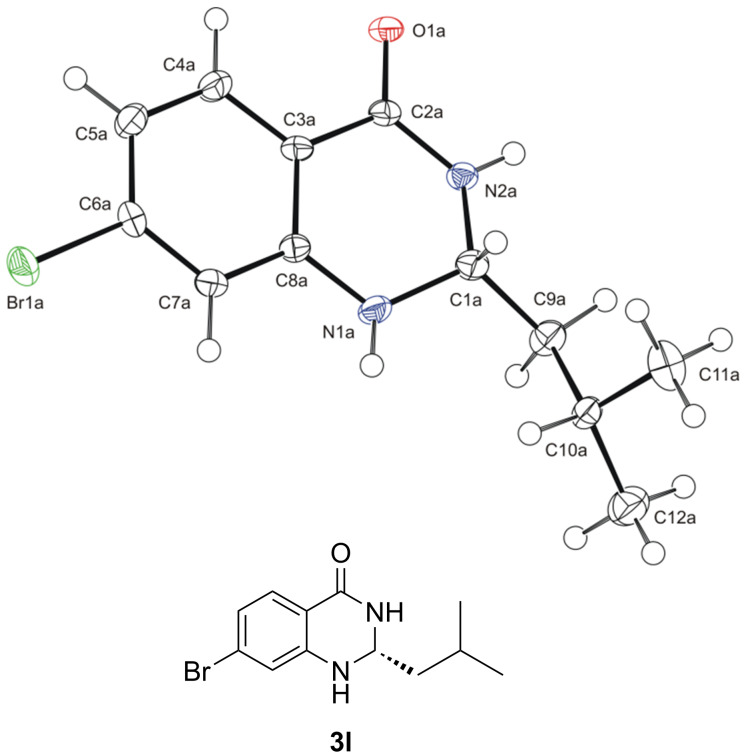
X-ray single-crystal structure of aminal **3l** with the displacement ellipsoids drawn at the 30% probability level.

To determine the absolute configuration of aminals **3a–t**, derivative **3l** was subjected to X-ray crystallographic analysis. The absolute configuration of the stereogenic center (C1) was assigned as *R* (Figure 2, for details see Supporting Information File 1) [36], which is in agreement with the configuration of aminals obtained by List and co-workers [14].

## Conclusion

In summary, we have reported an organocatalytic asymmetric aminalization reaction between aldehydes and anthranilamides catalyzed by a PCCP catalyst as a cheap and readily available option to conventional chiral BINOL phosphoric acids. The reaction tolerates a wide range of substitutions of anthranilamides and aromatic and aliphatic aldehydes, yielding the corresponding dihydroquinazolinones in excellent yields (up to 97%) and enantiopurities up to 80% ee. We demonstrated that bulkiness of aldehydes negatively affected the enantiocontrol of the process, and highly enantiomerically enriched dihydroquinazolinones can be achieved by crystallization (up to 97% ee). The developed methodology can also be used to form tetrahydrobenzodiazepinones; however, a significant drop in the yield and enantioselectivity was observed.

## Supporting Information

File 1 General synthetic procedures, characterization of compounds, X-ray experimental data, and copies of ^1^H and ^13^C NMR spectra.
