# Supplementary material for: Enantioselective PCCP Brønsted acid-catalyzed aminalization of aldehydes
Source: Beilstein J Org Chem. 2021 Sep 16;17:2433–40. doi: 10.3762/bjoc.17.160 (PMC8450961; doi:10.3762/bjoc.17.160)
Supplement: File 1 — General synthetic procedures, characterization of compounds, X-ray experimental data, and copies of 1H and 13C NMR spectra. [file Beilstein_J_Org_Chem-17-2433-s001.pdf]

BEILSTEIN JOURNAL OF ORGANIC CHEMISTRY

# Supporting Information

for

# Enantioselective PCCP Brønsted acid-catalyzed aminalization of aldehydes

Martin Kamlar, Robert Reiberger, Martin Nigríni, Ivana Císařová and Jan Veselý

*Beilstein J. Org. Chem.* **2021,** *17,* 2433–2440. doi:10.3762/bjoc.17.160

## General synthetic procedures, characterization of compounds, X-ray experimental data, and copies of $^{1}$H and $^{13}$C NMR spectra

License and Terms: This is a supporting information file under the terms of the Creative Commons Attribution License (https://creativecommons.org/licenses/by/4.0). Please note that the reuse, redistribution and reproduction in particular requires that the author(s) and source are credited and that individual graphics may be subject to special legal provisions.
The license is subject to the *Beilstein Journal of Organic Chemistry* terms and conditions: (https://www.beilstein-journals.org/bjoc/terms)

# Table of contents

Table of contents ........................................ S1
General ........................................ S2
Starting materials ........................................ S3
Preparation of PCCP catalysts ........................................ S3
Preparation of anthranilamide derivatives ........................................ S5
General procedure for aminalization of aldehydes ........................................ S6
NMR spectra ........................................ S14
HPLC chromatograms ........................................ S45
X-Ray section ........................................ S65
References ........................................ S67

# General

Chemicals and solvents were either purchased puriss *p.a.* from commercial suppliers or purified by standard techniques. For thin-layer chromatography (TLC), silica gel plates Merck 60 F254 were used and compounds were visualized by irradiation with UV light and/or by treatment with a solution of phosphomolybdic acid (AMC) or vaniline followed by heating. The solution of AMC was prepared from phosphomolybdic acid (25 g), $Ce(SO_4)_2 \cdot H_2O$ (10 g), conc. $H_2SO_4$ (60 mL) and $H_2O$ (940 mL). The solution of vanilline was prepared from vanilline (15 g) in ethanol (250 mL) and conc. sulfuric acid (2.5 mL). Column chromatography was performed using silica gel from Fluka (40–63 µm). $^{1}$H, $^{19}$F, and $^{13}$C NMR spectra were recorded with a Bruker AVANCE III 400. Chemical shifts for protons are given in δ and are referenced to residual protium in the NMR solvent (chloroform-*d*: δ = 7.26 ppm, DMSO-$d_6$: δ = 2.50 ppm, acetonitrile-$d_3$ = 1.94 ppm). Chemical shifts for carbon are referenced to the carbon in NMR solvent (chloroform-*d*: δ = 77.0 ppm, DMSO-$d_6$: δ = 39.5 ppm, acetonitrile-$d_3$ = 118.2 ppm). The coupling constants *J* are given in Hz. Chiral HPLC was carried out using a LC20AD Shimadzu liquid chromatograph with an SPD-M20A diode array detector with columns Daicel Chiralpak® IA, Daicel Chiralpak® IB, Daicel Chiralpak® AD, Daicel Chiralpak® ODH, Daicel Chiralpak® IG. Optical rotations were measured on AU-Tomatica polarimeter, Autopol III. Specific optical rotations are given in concentrations *c* [g/100 mL]. IR DRIFT spectras were recorded with a Nicolet AVATAR 370 FT-IR in $cm^{-1}$. High-resolution mass spectra were recorded with a LCQ Fleet spectrometer.

# Starting materials

## Preparation of PCCP catalysts

**Tetramethyl 5-(hydroxy(methoxy)methylene)cyclopenta-1,3-diene-1,2,3,4-tetracarboxylate (I):**

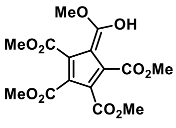


I

Compound **I** was prepared according to literature[1]; **$^{1}$H-NMR** (400 MHz, $CDCl_3$): $\delta_H$ = 20.09 (s, 1H), 4.04 (s, 6H), 3.90 (s, 6H), 3.76 (s, 3H) ppm; **$^{13}$C-NMR** (101 MHz, $CDCl_3$) δ = 172.4 (2C), 167.8 (2C), 163.3, 133.7 (2C), 117.0, 106.4 (2C), 55.7 (2C), 52.7 (2C), 52.0 ppm; **MS** (ESI+) *m*/*z*: calc. for $C_{15}H_{15}O_{10}$ [M-H]$^-$: 355.1, found: 355.0.

**Tetrakis((1*R*,2*S*,5*R*)-2-isopropyl-5-methylcyclohexyl) 5-(hydroxy(((1*R*,2*S*,5*R*)-2-isopropyl-5-methylcyclohexyl)oxy)methylene)cyclopenta-1,3-diene-1,2,3,4-tetracarboxylate (II):**

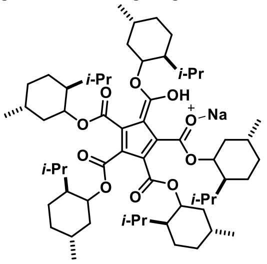


II

Compound **II** was prepared according to the published procedure[1]; **$^{1}$H-NMR** (400 MHz, $CDCl_3$) $\delta_H$ = 20.30 (s, 1H), 5.11 – 4.63 (m, 5H), 2.72 – 0.40 (m, 90H) ppm; **13C NMR** $\delta_C$ = 172.1 (2C) 167.1 (2C), 162.7, 134.3 (2C), 118.8, 106.5 (2C), 81.2 (2C), 76.6 (2C), 75.7, 47.5 (2C), 46.2 (3C), 41.6 (2C), 40.8, 40.3 (2C), 34.4-34.0 (5C), 32.0-31.7 (5C), 25.6-25.4 (5C), 23.3-21.0 (15C), 16.6-15.7 (5C) ppm; **HRMS** (ESI+) *m*/*z*: calc. for $C_{60}H_{96}O_{10}Na$ [M+Na]$^+$: 999.7, found: 999.9.

**Trimethyl (*E*)-5-(hydroxy(((*S*)-1,2,3,4-tetrahydronaphthalen-1-yl)amino)methylene)-4-(((*S*)-1,2,3,4-tetrahydronaphthalen-1-yl)carbamoyl)cyclopenta-1,3-diene-1,2,3-tricarboxylate (III):**

In a dry flask PCCP **I** (300 mg, 0.84 mmol, 1.0 equiv) and (*S*)-(+)-1,2,3,4-tetrahydro-1-naphthylamine (0.22 mL, 1.54 mmol, 2.0 equiv) were dissolved in dry toluene (8.4 mL). Then, the reaction mixture was refluxed for 45 min. After cooling to room temperature solvents were evaporated on a rotavap. The crude product was purified by column chromatography ($CH_2Cl_2$/MeOH 20:1). The combined organic phases were washed with 1 M HCl (3 × 25 mL), dried over anhydrous $MgSO_4$, and the solvents were evaporated *in vacuo* to give the desired product **III** as red-brown syrup in 42% yield (206 mg).

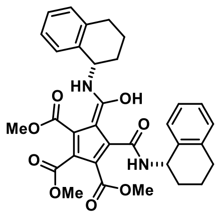


III

Red-brown syrup, 42 % yield (206 mg); **R*f*** = 0.89 ($CH_2Cl_2$/MeOH = 7:1, detected in vanilline). **$^{1}$H-NMR** (600 MHz, $CDCl_3$): $\delta_H$ = 19.94 (s, 1H), 11.43 (s, 2H), 7.25 – 7.08 (m, 8H), 5.36 (d, *J* = 6.7 Hz, 2H), 3.79 (s, 3H), 3.69 (s, 6H), 2.91 (dt, *J* = 17.0, 6.2 Hz, 2H), 2.80 (dt, *J* = 16.9, 6.3 Hz, 2H), 2.15 (td, *J* = 7.5, 6.4, 3.5 Hz, 2H), 1.99 (dt, *J* = 12.8, 7.0 Hz, 4H), 1.94 – 1.85 (m, 2H) ppm; **$^{13}$C-NMR** (151 MHz, $CDCl_3$): $\delta_C$ = 168.9, 168.6 (2C), 167.5 (2C), 137.5 (2C), 135.4 (2C), 131.7, 129.4 (2C), 128.8 (2C), 127.7 (2C), 126.3 (2C), 117.8, 115.3 (2C), 52.8 (2C), 52.1 (2C), 49.7 (2C), 29.8 (2C), 29.2 (2C), 20.2 (2C) ppm; **IR** (KBr): ν = 3431, 2950, 2863, 1739, 1631, 1607, 1440, 1350, 1299, 1222, 1162, 1099, 1072, 1024, 1003 cm$^{-1}$; $[\alpha]_D^{20}$ = –14.2 (*c* = 0.53; MeOH); **HRMS** (ESI-) *m*/*z*: calc. for $C_{33}H_{34}N_2O_8$ [M-H]$^-$: 585.2242, for: 585.2251.

**Tetramethyl 5-((((1*R*,2*R*)-2-(3-(3,5-bis(trifluoromethyl)phenyl)thioureido)cyclohexyl)amino)(hydroxy)methylene)cyclopenta-1,3-diene-1,2,3,4-tetracarboxylate (IV):**

In a dry flask PCCP **I** (200 mg, 0.561 mmol, 1.0 equiv) and 1-((1*R*,2*R*)-2-aminocyclohexyl)-3-(3,5-bis(trifluoromethyl)phenyl)thiourea (216 mg, 0.561 mmol, 1.0 equiv) were dissolved in dry toluene (7 mL). Then the reaction mixture was refluxed for 60 min. After cooling to room temperature, solvents were evaporated on a rotavap. The crude product was purified by column chromatography ($CH_2Cl_2$/MeOH 20:1). The combined organic phases were washed with 1 M HCl (3 × 25 mL), dried over anhydrous $MgSO_4$, and the solvents were evaporated *in vacuo* to give the desired product **IV** as brown solid in 82% yield (330 mg).

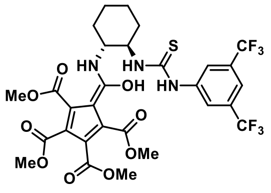


IV

Brown solid**,** yield 82% (330 mg), m.p. 67-68 °C; $\mathbf{R}_f$ = 0.89 ($CH_2Cl_2$/MeOH = 7:1, detected in vanilline).**$^1$H-NMR** (400 MHz, MeOD) $\delta_H$ 8.25 (s, 2H), 7.67 (s, 1H), 4.60 (bs, 1H), 3.73 (s, 12H), 2.91 (ddd, *J* = 11.8, 10.6, 4.2 Hz, 1H), 2.20 – 2.00 (m, 2H), 1.83 (d, *J* = 10.2 Hz, 2H), 1.53 (q, *J* = 12.3, 11.8 Hz, 1H), 1.48 – 1.27 (m, 3H) ppm; **$^{13}$C-NMR** (101 MHz, MeOD): $\delta_C$ = δ 183.6, 169.9, 169.86 (3C), 142.96 (2C), 132.63 (q, *J* = 33.4 Hz, 2C), 124.7 (q, *J* = 273 Hz, 2C); 124.4 (3C), 118.4 (2C), 118.3 (q, *J* = 4 Hz, 2C), 64.1, 56.5, 56.3, 52.0 (4C), 32.1, 31.1, 25.5, 24.7 ppm; **$^{19}$F NMR** (376 MHz, MeOD): $\delta_F$ -64.5; **IR** (KBr): ν = 3550, 3311, 3049, 3005, 2951, 2868, 2787, 1699, 1601, 1545, 1469, 1385, 1360, 1329, 1279, 1219, 1178, 1134, 1109, 1074 $cm^{-1}$; $[\boldsymbol{\alpha}]_{\mathbf{D}}^{\mathbf{20}}$ = – 40.8 (*c* = 2.04; DMSO); **HRMS** ($ESI^-$) *m*/*z*: calc. for $C_{29}H_{28}F_6N_3O_9S$ $[M-H]^-$: 708.1529, for: 708.1531.

## Preparation of anthranilamide derivatives

2-Amino-4-bromobenzamide (**1l**) and 2-amino-5-methylbenzamide (**1r**) and were prepared according to the literature[2], 2-amino-6-bromobenzamide (**1n**) was prepared according to the published procedure[3], 2-(2-aminophenyl)acetamide (**1t**) was prepared according to the published procedure[4] and 2-(benzylamino)benzamide (**1u**) was prepared according to the published procedure[5].

**2-Amino-4-bromobenzamide** (**1l**)

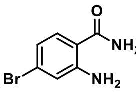

1l

[6]. **$^{1}$H-NMR** (400 MHz, DMSO-d$_6$) $\delta_H$ = 7.78 (s, 1H), 7.46 (d, *J* = 8.5 Hz, 1H), 7.15 (s, 1H), 6.99 – 6.86 (m, 1H), 6.80 (s, 2H), 6.71 – 6.57 (m, 1H) ppm; **$^{13}$C-NMR** (101 MHz, DMSO-d$_6$) $\delta_C$ = 170.5, 151.6, 130.7, 125.3, 118.1, 116.8, 112.7 ppm; **MS** (ESI+) *m*/*z*: calc. for $C_7H_6BrN_2ONa$ [M-H+Na]$^-$: 236.0, found: 236.2.

**2-Amino-6-bromobenzamide** (**1n**)

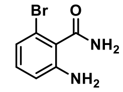

1n

Characterization according to the literature[7]. **$^{1}$H-NMR** (400 MHz, $CDCl_3$) ) $\delta_H$ = 6.98 (t, *J* = 8.0 Hz, 1H), 6.91 (dd, *J* = 7.9 Hz, *J´* = 1.0 Hz, 1H), 6.63 (dd, *J* = 8.0 Hz, *J´* = 1.0 Hz, 1H), 6.03 (s, 2H), 4.59 (s, 2H) ppm; **$^{13}$C-NMR** (101 MHz, $CDCl_3$) $\delta_C$ = 169.5, 147.5, 131.7, 122.4, 121.2, 119.9, 115.5 ppm; **MS** (ESI+) *m*/*z*: calc. for $C_7H_8BrN_2O$ [M+H]$^+$: 215.0, found: 215.0.

**2-Amino-5-methylbenzamide** (**1r**)

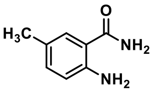

1r

Characterization according to the literature[2]. **$^{1}$H-NMR** (400 MHz, DMSO-d$_6$) $\delta_H$ = 7.67 (d, *J* = 15.3 Hz, 1H), 7.41 – 7.30 (m, 2H), 6.98 (s, 1H), 6.95 (dd, *J* = 8.3 Hz, *J´* = 1.8 Hz, 1H), 6.59 (d, *J* = 8.3 Hz, 1H), 6.31 (s, 2H), 2.14 (s, 3H) ppm; **$^{13}$C-NMR** (101 MHz, DMSO-d$_6$) $\delta_C$ = 171.3, 147.9, 132.7, 128.7, 122.7, 116.5, 113.8, 20.0 ppm; **MS** (ESI+) *m*/*z*: calc. for $C_8H_{10}N_2ONa$ [M+Na]$^+$: 173.1, found: 173.1.

**2-(2-Aminophenyl)acetamide (1t)**

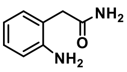

1t

Brown solid, yield 50% (110 mg), m.p. 140-141 °C (from MeOH); **$^{1}$H-NMR** (400 MHz, $CDCl_3$) ) $\delta_H$ = 7.11 (td, *J* = 7.7 Hz, *J´* = 1.5 Hz, 1H), 7.07 – 7.01 (m, 1H), 6.78 – 6.67 (m, 2H), 5.75 (bs, 2H), 4.05 (bs, 1H), 3.47 (s, 2H) ppm; **$^{13}$C-NMR** (101 MHz, $CDCl_3$) $\delta_C$ = 173.9, 145.5, 131.0, 128.9, 120.2, 119.2, 116.6, 40.5 ppm; IR (KBr): ν = 3348, 3400, 3195, 1658, 1622, 1281 cm$^{-1}$; **HRMS** (ESI+) *m*/*z*: calc. for $C_8H_{11}N_2O$ [M+H]$^+$: 151.0866, found: 151.0865.

**2-(Benzylamino)benzamide (1u)**

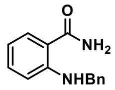

1u

Characterization according to the literature[5]. **$^{1}$H-NMR** (400 MHz, DMSO) $\delta_H$ = 8.59 (s, 1H), 7.86 (s, 1H), 7.62 (d, *J* = 7.2 Hz, 1H), 7.33 (d, *J* = 3.7 Hz, 3H), 7.28 – 7.16 (m, 4H), 6.61 (d, *J* = 8.2 Hz, 1H), 6.53 (t, *J* = 7.1 Hz, 1H), 4.38 (d, *J* = 5.3 Hz, 2H) ppm; **$^{13}$C-NMR** (101 MHz, DMSO) $\delta_C$ = 171.6, 149.6, 139.7, 132.5, 129.1, 128.5 (2C), 128.2, 127.1(2C), 126.8, 114.2, 111.5, 46.0 ppm**;** **MS** (ESI+) *m*/*z*: calc. for $C_{14}H_{15}N_2O$ [M+H]$^+$: 227.1, found: 227.1.

# General procedure for the aminalization of aldehydes

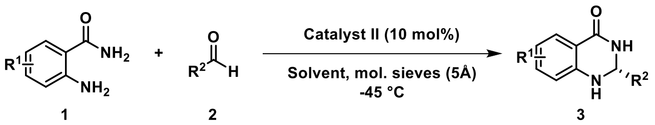

*General procedure*:
To the amide **1** (0.1 mmol, 1.0 equiv) in a dry flask, catalyst **II** (10 mg, 0.01 mmol, 0.1 equiv) and molecular sieves (5 Å, 30 mg) were added. The reaction mixture was degassed and filled with argon. Solids were dissolved in dry toluene or THF (1 mL), and the resulted solution was cooled to −45 °C followed by the dropwise addition of the corresponding aldehyde **2** (0.1 mmol, 1.0 equiv) dissolved in dry toluene or THF (1 mL). Then, the reaction mixture was allowed to stir at the indicated temperature until complete consumption of starting material was observed. The reaction mixture was then directly loaded on silica gel and purified by column chromatography (*n*-hexane/EtOAc) to give the desired aminals **3a**–**t**.

**(*R*)-2-Isobutyl-2,3-dihydroquinazolin-4(1*H*)-one (3a):**

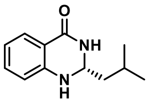

3a

The title compound **3a** was prepared according to the general procedure (reaction time: 20 hours, solvent: toluene, mobile phase (*n*-hexane/EtOAc 3:1 to 2:1), affording the title compound as white solid in yield 96 % (19.5 mg), m.p. 144-145 °C (from EtOAc), 81 % (93% after recrystallization) *ee*; $\mathbf{R}_f$ = 0.39 (*n*-hexane/EtOAc 1:1). **$^{1}$H-NMR** (400 MHz, ($CDCl_3$): $\delta_H$ = 7.87 (dd, *J* = 7.8 Hz, *J´* = 1.5 Hz, 1H), 7.28 (ddd, *J* = 8.4 Hz, *J´* = 7.5, 1.7 Hz, 1H), 6.89 (s, 1H), 6.88 – 6.79 (m, 1H), 6.71 – 6.64 (m, 1H), 4.91 (tt, *J* = 6.5 Hz, *J´* = 1.6 Hz, 1H), 4.35 (s, 1H), 1.80 (dp, *J* = 13.2 Hz, *J´* = 6.6 Hz, 1H), 1.73 – 1.60 (m, 2H), 0.97 (d, *J* = 1.3 Hz, 3H), 0.95 (d, *J* = 1.3 Hz, 3H) ppm; **$^{13}$C-NMR** (101 MHz, $CDCl_3$) $\delta_C$ = 165.7, 147.6, 133.8, 128.6, 119.4, 116.4, 115.0, 63.7, 44.5, 23.9, 22.8, 22.7 ppm; $[\alpha]_D^{20}$ = –107.7 (*c* = 0.39, THF); **Enantiomeric excess** (80 % *e.e.*) was determined by HPLC using chiral OD-H column (mobile phase: *n*-heptane/propan-2-ol = 80:20, λ = 210 nm, *V* = 1 mL/min, *T* = 25 °C), $t_R$ = 8.1 min (*minor. enantiomer),* $t_R$ = 10.1 min (*major. enantiomer)*; **MS** (ESI+) *m*/*z*: calc. for $C_{12}H_{16}N_2O$ $[M+Na]^+$: 227, found: 227.

***(R)*-2-Cyclohexyl-2,3-dihydroquinazolin-4(1*H*)-one (3b)**

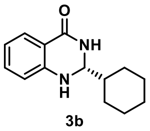

3b

The title compound **3b** was prepared according to the general procedure (reaction time: 40 hours, solvent: toluene, mobile phase (*n*-hexane/EtOAc 1:1), affording the title compound as white semi-solid in yield 96 % (22 mg), 74 % *ee.*; $\mathbf{R}_f$ = 0.25 (*n*-hexane/EtOAc 1:1). **$^{1}$H-NMR** (400 MHz, ($CDCl_3$): $\delta_H$ = 7.86 (d, *J* = 7.8 Hz, 1H), 7.31 – 7.25 (m, 1H), 6.85 – 6.76 (m, 1H), 6.65 (d, *J* = 8.1 Hz, 1H), 6.33 (bs, 1H), 4.63 (d, *J* = 5.0 Hz, 1H), 4.31 (bs, 1H), 1.94 – 1.56 (m, 6H), 1.44 – 0.99 (m, 5H) ppm; **$^{13}$C-NMR** (101 MHz, $CDCl_3$) $\delta_C$ = 165.4, 147.5, 133.9, 128.6, 119.1, 115.8, 114.6, 69.7, 42.8, 27.6, 26.3, 25.9 ppm; $[\alpha]_D^{20}$ = –68.2 (*c* = 0.33, THF); **Enantiomeric excess** (74 % *e.e.*) was determined by HPLC using chiral IA column (mobile phase: *n*-heptane/propan-2-ol 80:20, λ = 190 nm, *V* = 1 mL/min, *T* = 25 °C), $t_R$ = 7.9 min (*minor enantiomer),* $t_R$ = 9.7 min (*major enantiomer*; **MS** (ESI+) *m*/*z*: calc. for $C_{14}H_{18}N_2ONa$ $[M+Na]^+$: 253.1, found: 253.2.

***(R)*-2-*tert*-Butyl-2,3-dihydroquinazolin-4(1*H*)-one (3c)**

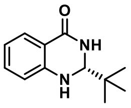

3c

The title compound **3c** was prepared according to the general procedure (reaction time: 72 hours, solvent: toluene, mobile phase (*n*-hexane/EtOAc = 1:1), affording the title compound as white solid in yield 95 % (19.0 mg), m.p. 156-159 °C (from EtOAc), 10% *ee.*; $\mathbf{R}_f$ = 0.30 (*n*-hexane/EtOAc 1:1). **$^{1}$H-NMR** (400 MHz, ($CDCl_3$): $\delta_H$ = 7.85 (dd, *J* = 7.8 Hz, *J´* = 1.3 Hz, 1H), 7.27 (ddd, *J* = 8.7 Hz, *J´* = 7.6 Hz, *J´´* = 1.5 Hz, 1H), 6.83 – 6.75 (m, 1H), 6.65 (d, *J* = 8.0 Hz, 1H), 6.24 (s, 1H), 4.57 (s, 1H), 4.32 (s, 1H), 1.01 (s, 6H) ppm; **$^{13}$C-NMR** (101 MHz, $CDCl_3$) $\delta_C$ = 165.5, 147.7, 134.0, 128.6, 119.0, 115.1, 114.4, 73.6, 35.5, 24.7 ppm; $[\alpha]_D^{20}$ = –4.0 (*c* = 0.25, THF); **Enantiomeric excess** (10 % *e.e.*) was determined by HPLC using chiral IH column (mobile phase: *n*-heptane/propan-2-ol 80:20, λ = 190 nm, *V* = 1 mL/min, *T* = 25 °C), $t_R$ = 12.1 min (*minor enantiomer),* $t_R$ = 13.9 min (*major enantiomer)*; **HRMS** (ESI+) *m*/*z*: calc. for $C_{12}H_{17}N_2O$ $[M+H]^+$: 205.1335, found: 205.1336.

**(*R*)-2-Butyl-2,3-dihydroquinazolin-4(1*H*)-one (3d):**

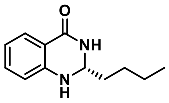

3d

The title compound **3d** was prepared according to the general procedure (reaction time: 21 hours, solvent: toluene, mobile phase (*n*-hexane/EtOAc 3:1 to 2:1), affording the title compound as white semi-solid in yield 97 % (19.7

mg) and 76 % *ee*. $\mathbf{R}_f$ = 0.39 (*n*-hexane/EtOAc 1:1, detected in vanilline). **$^1$H-NMR** (400 MHz, $CDCl_3$): $\delta_H$ = 7.87 (d, *J* = 7.7 Hz, 1H), 7.32 – 7.27 (m, 1H), 6.89 – 6.80 (m, 1H), 6.13 (s, 1H), 4.87 (t, *J* = 5.8 Hz, 1H), 4.20 (s, 1H), 1.90 – 1.71 (m, 2H), 1.40 (dq, *J* = 7.0 Hz, *J´* = 3.6 Hz, 4H), 0.94 (t, *J* = 7.0 Hz, 3H) ppm; **$^{13}$C-NMR** (101 MHz, $CDCl_3$) $\delta_C$ = 165.3, 147.5, 133.9, 128.8, 119.6, 116.1, 114.9, 65.5, 35.5, 26.3, 22.6, 14.1 ppm; $[\alpha]_D^{20}$ = –97,8 (*c* = 0.23, THF); **Enantiomeric excess** (76 % *e.e.*) was determined by HPLC using chiral IG column (mobile phase: *n*-heptane/propan-2-ol 80:20, λ = 200 nm, *V* = 1 mL/min, *T* = 25 °C), $t_R$ = 9.3 min (*minor enantiomer),* $t_R$ = 10.1 min (*major enantiomer);* **MS** (ESI+) *m*/*z*: calc. for $C_{12}H_{16}N_2O$ [M + Na]$^+$: 227, found: 227.

**(*R*)-2-Phenyl-2,3-dihydroquinazolin-4(1*H*)-one (3e):**

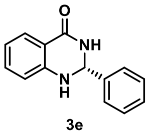

3e

The title compound **3e** was prepared according to the general procedure (reaction time: 96 hours, solvent: toluene, mobile phase (*n*-hexane/EtOAc 3:1), affording the title compound as white semi-solid in the yield 77 % (17.3 mg), 68 % *ee.* $\mathbf{R}_f$ = 0.48 (*n*-hexane/EtOAc = 1:1). **$^1$H-NMR** (400 MHz, $(CD_3)_2SO$ ): $\delta_H$ = 8.28 (t, *J* = 2.0 Hz, 1H), 7.61 (dd, *J* = 7.8, 1.6 Hz, 1H), 7,55 – 7.47 (m, 2H), 7.45 – 7.31 (m, 3H), 7.24 (ddd, *J* = 8.6, 7.2, 1.6 Hz, 1H), 7.11 (s, 1H), 6.75 (dd, *J* = 8.1, 1.0 Hz, 1H), 6.67 (td, *J* = 7.4, 1.1 Hz, 1H) ppm; $[\alpha]_D^{20}$ = –135.3 (*c* = 0.26; THF); **Enantiomeric excess** (68 % *e.e.*) was determined by HPLC using chiral AD-H column (mobile phase: *n*-heptane/propan-2-ol = 80:20, λ = 228 nm, *V* = 1 mL/min, *T* = 25 °C), $t_R$ = 11.7 min (*minor enantiomer)*, $t_R$ = 13.7 min (*major enantiomer)*; **MS** (ESI+) *m*/*z*: calc. for $C_{14}H_{12}N_2O$ [M + Na]$^+$: 247, found: 246.

**(*R*)-2-Tolyl-2,3-dihydroquinazolin-4(1*H*)-one (3f):**

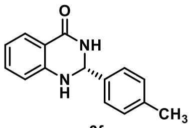

3f

The title compound **3f** was prepared according to the general procedure (reaction time: 112 hours, solvent: toluene, mobile phase (*n*-hexane/EtOAc 2:1)), affording the title compound as white solid in the yield 83 % (20 mg), m.p. 222 °C (from EtOAc), 70% (97% after recrystallization) *e.e.* $\mathbf{R}_f$ = 0.25 (*n*-hexane/EtOAc = 1:1). **$^1$H-NMR** (400 MHz, ($CDCl_3$): $\delta_H$ = 7.95 (d, *J* = 6.6 Hz, 1H), 7.48 (d, *J* = 8.0 Hz, 2H), 7.37 – 7.30 (m, 1H), 7.25 (d, *J* = 8.2 Hz, 2H), 6.90 (t, *J* = 7.5 Hz, 1H), 6.66 (d, *J* = 8.0 Hz, 1H), 5.87 (s, 1H), 5.77 (s, 1H), 4.35 (s, 1H), 2.40 (s, 3H) ppm; **$^{13}$C-NMR** (101 MHz, $CDCl_3$) $\delta_c$ = 165.0, 147.5, 140.4, 135.8, 134.1, 129.9, 128. 9, 127.5, 119.8, 114.7, 69.1, 21.4 ppm; $[\alpha]_D^{20}$ = –52.5 (*c* = 0.20; THF); **Enantiomeric excess** (70 % *e.e.*) was determined by HPLC using chiral IA column (mobile phase: *n*-heptane/propan-2-ol 90:10, λ = 190 nm, *V* = 1 mL/min, *T* = 25 °C), $t_R$ = 25. 9 min (*minor enantiomer)*, $t_R$ = 29.8 min (*major enantiomer)*; **HRMS** (ESI+) *m*/*z*: calc. for $C_{15}H_{15}N_2O$ [M+H]$^+$: 239.1179, found: 239.1181.

**(*R*)-2-(*m*-Tolyl)-2,3-dihydroquinazolin-4(1*H*)-one (3g):**

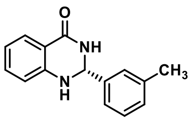

3g

The title compound **3g** was prepared according to the general procedure (reaction time: 30 hours, solvent: toluene, mobile phase (*n*-hexane/EtOAc 2:1), affording the title compound as white semi-solid in yield 75 % (18 mg), 36 % *e.e.*; $\mathbf{R}_f$ = 0.45 (*n*-hexane/EtOAc = 1:1). **$^1$H-NMR** (400 MHz, ($CDCl_3$): $\delta_H$ = 7.95 (dd, *J* = 7.8 Hz, *J´* = 1.6 Hz, 1H), 7.42 (d, *J* = 1.9 Hz, 1H), 7.38 – 7.30 (m, 3H), 7.25 (m, 1H), 6.90 (td, *J* = 7.5 Hz, *J´* = 1.0 Hz, 1H), 6.67 (dd, *J* = 8.1 Hz, *J´* = 1.0 Hz, 1H), 5.86 (s, 1H), 5.79 (s, 1H), 4.38 (s, 1H), 2.39 (s, 3H) ppm; **$^{13}$C-NMR** (101 MHz, $CDCl_3$) = $\delta_C$ = 164.9, 147.4, 139.2, 138.6, 134.2, 131.0, 129.2, 128.9, 128.2, 124.6, 119.8, 115.8, 114.7, 69.2, 21.5 ppm; $[\alpha]_D^{20}$ = -91.8 (c = 0.31, THF); **Enantiomeric**

**excess** (36% *e.e.*) was determined by HPLC using chiral IA column (mobile phase: n-heptane/propan-2-ol 90:10, λ = 225 nm, V = 1 mL/min, T = 25 °C), $t_R$ = 21.2 min (minor enantiomer), $t_R$ = 24.6 min (major enantiomer); **LC-MS** (ESI$^+$) m/z: calc. for $C_{15}H_{15}N_2O$ [M+]$^+$: 239, found: 239.

**(*R*)-2-(*o*-Tolyl)-2,3-dihydroquinazolin-4(1*H*)-one (3h):**

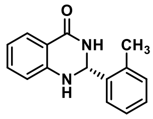

3h

The title compound **3h** was prepared according to the general procedure (reaction time: 72 hours, solvent: toluene, mobile phase (*n*-hexane/EtOAc 2:1 to 3:2), affording the title compound as pink semi-solid in yield 88 % (21 mg), 20 % *e.e.*; **R*f*** = 0.45 (n-hexane/EtOAc = 1:1). **$^1$H-NMR** (400 MHz, ($CDCl_3$): $\delta_H$ = 7.93 (dd, *J* = 7.8 Hz, *J´* = 1.6 Hz, 1H), 7.71 (dd, *J* = 7.2 Hz, *J´* = 2.0 Hz, 1H), 7.36 – 7.27 (m, 3H), 7.23 – 7.19 (m, 1H), 6.89 (td, *J* = 7.5 Hz, *J´* = 1.1 Hz, 1H), 6.69 (dd, *J* = 8.1 Hz, *J´* = 1.1 Hz, 1H), 6.14 (s, 1H), 5.77 (s, 1H), 4.43 (s, 1H), 2.46 (s, 3H) ppm; **$^{13}$C-NMR** (101 MHz, $CDCl_3$) = $\delta_C$ 165.2, 147.8, 136.5, 136.0, 134.1, 131.4, 129.8, 128.9, 127.8, 126.9, 119.7, 115.9, 114.9, 66.0, 19.2 ppm; $[\alpha]_D^{20}$ = -30.9 (c = 0.38, THF); **Enantiomeric excess** (20 % *e.e.*) was determined by HPLC using chiral IA column (mobile phase: *n*-heptane/propan-2-ol 90:10, λ = 225 nm, V = 1 mL/min, T = 25 °C), $t_R$ = 21.2 min (*minor enantiomer*), $t_R$ = 31.9 min (*major enantiomer*); **LC-MS** (ESI$^+$) m/z: calc. for $C_{15}H_{15}N_2O$ [M+]$^+$: 239, found: 239.

**(*R*)-2-(4-Fluorophenyl)-2,3-dihydroquinazolin-4(1*H*)-one (3i):**

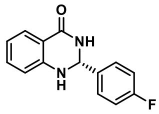

3i

The title compound **3i** was prepared according to the general procedure (reaction time: 40 hours, solvent: toluene, mobile phase (*n*-hexane/EtOAc 2:1)), affording the title compound as white solid in the yield 58 % (14 mg), m.p. 269-271 °C (from EtOAc), 48% *e.e.* **R*f*** = 0.3 (*n*-hexane/EtOAc = 1:1). **$^1$H-NMR (400 MHz, DMSO-d$_6$)** $\delta_H$ = 8.28 (s, 1H), 7.61 (dd, *J* = 7.8 Hz, *J´* = 1.5 Hz, 1H), 7.57 – 7.49 (m, 2H), 7.31 – 7.18 (m, 3H), 7.09 (s, 1H), 6.74 (d, *J* = 8.1 Hz, 1H), 6.72 – 6.57 (m, 1H), 5.77 (s, 1H). ppm; **$^{13}$C-NMR** (101 MHz, DMSO-d$_6$) $\delta_C$ = 163.6, 162.1 (d, *J* = 244.1 Hz), 147.8, 137.8 (d, *J* = 2.9 Hz), 133.4, 129.0 (d, *J* = 8.4 Hz, 2C), 127.4, 117.3, 115.1 (d, *J* = 21.5 Hz, 2C), 115.0, 114.5, 65.9 ppm; **$^{19}$F-NMR** (376 MHz, DMSO-d$_6$) δ = -110.46 (m); $[\alpha]_D^{20}$ = –61.0 (*c* = 0.16; THF); **Enantiomeric excess** (48 % *e.e.*) was determined by HPLC using chiral IC column (mobile phase: *n*-heptane/propan-2-ol 80:20, λ = 225 nm, *V* = 1 mL/min, *T* = 25 °C), $t_R$ = 12.2 min (*major enantiomer)*, $t_R$ = 14.5 min (*minor enantiomer)*; **LC-MS** (ESI$^+$) *m/z*: calc. for $C_{14}H_{12}FN_2O$ [M+H]$^+$: 243, found: 243.

**(*R*)-2-(4-Chlorophenyl)-2,3-dihydroquinazolin-4(1*H*)-one (3j):**

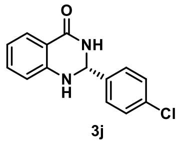

3j

The title compound **3j** was prepared according to the general procedure (reaction time: 45 hours, solvent: toluene, mobile phase (*n*-hexane/EtOAc 2:1)), affording the title compound as white solid in the yield 69 % (18 mg), m.p. 228-230 °C (from EtOAc), 53% *e.e.* **R*f*** = 0.28 (*n*-hexane/EtOAc = 1:1). **$^1$H-NMR (**400 MHz, DMSO-d$_6$) $\delta_H$ = 8.32 (s, 1H), 7.61 (dd, *J* = 7.7 Hz, *J´* = 1.5 Hz, 1H), 7.52 – 7.49 (m, 2H), 7.48 – 7.43 (m, 2H), 7.25 (ddd, *J* = 8.2 Hz, *J´* = 7.2 Hz, *J´´* = 1.6 Hz, 1H), 7.14 (s, 1H), 6.74 (d, *J* = 8.1 Hz, 1H), 6.71 – 6.64 (m, 1H), 5.77 (s, 1H) ppm; **$^{13}$C-NMR** (101 MHz, DMSO-d$_6$) $\delta_C$ = 163.5, 147.6, 140.7, 133.4, 133.0, 128.8 (2C), 128.3 (2C), 127.4, 117.3, 114.9, 114.5, 65.7 ppm; $[\alpha]_D^{20}$ = –33.6 (*c* = 0.21; THF); **Enantiomeric excess** (53 % *e.e.*) was determined by HPLC using chiral IA column (mobile phase: *n*-heptane/propan-2-ol 80:20, λ = 224 nm, *V* = 1 mL/min, *T* = 25 °C), $t_R$ = 10.3 min (*minor enantiomer)*, $t_R$ = 13.0 min (*major enantiomer)*; **LC-MS** (ESI$^+$) *m/z*: calc. for $C_{14}H_{12}ClN_2O$ [M+H]$^+$: 259, found: 259.

**(*R*)-8-Bromo-2-isobutyl-2,3-dihydroquinazolin-4(1*H*)-one (3k):**

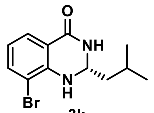

3k

The title compound **3k** was prepared according to the general procedure (reaction time: 96 hours, solvent: toluene, mobile phase (*n*-hexane/EtOAc 2:1 to 1:1), affording the title compound as white solid in yield 71 % (20 mg), m.p. 142-143 °C (from EtOAc), 30 % *ee.* $\mathbf{R}_f$ = 0.5 (*n*-hexane/EtOAc = 1:1). **$^1$H-NMR** (400 MHz, ($CDCl_3$): $\delta_H$ = 7.89 – 7.82 (m, 1H), 7.53 (dd, *J* = 7.9 Hz, *J´* = 1.4 Hz, 1H), 6.73 (t, *J* = 7.8 Hz, 1H), 6.53 (s, 1H), 4.98 (tt, *J* = 6.4 Hz, *J´* = 1.5 Hz, 1H), 4.77 (s, 1H), 1.82 (dq, *J* = 12.9 Hz, *J´* = 6.5 Hz, 1H), 1.75 – 1.68 (m, 1H), 1.02 (s, 3H), 1.00 (s, 3H) ppm; **$^{13}$C-NMR** NMR (101 MHz, $CDCl_3$) δ = 164.5, 144.9, 136.7, 128.1, 119.7, 117.3, 108.9, 63.6, 44.6, 24.1, 22.7 ppm; IR (KBr): ν = 3402, 3305, 2964, 1684, 1383, 748 $cm^{-1}$; $[\boldsymbol{\alpha}]_{\mathbf{D}}^{\mathbf{20}}$ = –8.6 (*c* = 0.29; THF); **enantiomeric excess** (30 % *e.e.*) was determined by HPLC using chiral IA column (mobil phase: *n*-heptane/propan-2-ol 80:20, λ = 190 nm, *V* = 1 mL/min, *T* = 25 °C), $t_R$ = 5.0 min (*minor. enantiomer)*, $t_R$ = 5.9 min (*major enantiomer)*; HRMS (ESI+) m/z: calc. for $C_{12}H_{15}BrN_2NaO$ $[M+Na]^+$: 305.0260, found: 305.0266.

**(*R*)-7-Bromo-2-isobutyl-2,3-dihydroquinazolin-4(1*H*)-one (3l):**

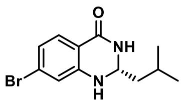

3l

The title compound **3l** was prepared according to the general procedure (reaction time: 96 hours, solvent: toluene, mobile phase (*n*-hexane/EtOAc 2:1)), affording the title compound as white solid in yield 89 % (25 mg), m.p. 166 °C (from EtOAc), 70% *ee.* $\mathbf{R}_f$ = 0.33 (*n*-hexane/EtOAc = 1:1). **$^1$H-NMR** (400 MHz, ($CDCl_3$): $\delta_H$ = 7.72 (d, *J* = 8.3 Hz, 1H), 6.96 (d, *J* = 8.3 Hz, 1H), 6.85 (d, *J* = 1.6 Hz, 1H), 6.61 (bs, 1H), 4.92 (t, *J* = 6.2 Hz, 1H), 4.34 (s, 1H), 1.78 (tq, *J* = 15.2 Hz, *J´* = 8.6 Hz, *J´´* = 7.7 Hz, 1H), 1.71 – 1.61 (m, 2H), 0.98 (s, 3H), 0.96 (s, 3H) ppm; **$^{13}$C-NMR** NMR (101 MHz, $CDCl_3$) δ = 164.8, 148.3, 130.2, 128.4, 122.7, 117.6, 115.1, 63.8, 44.6, 24.0, 22.7, 22.7 ppm; IR (KBr): ν = 3305, 3197, 2870, 1651, 1375, 1265 $cm^{-1}$; $[\boldsymbol{\alpha}]_{\mathbf{D}}^{\mathbf{20}}$ = –63.6 (*c* = 0.30; THF); **enantiomeric excess** (70 % *e.e.*) was determined by HPLC using chiral IA column (mobil phase: *n*-heptane/propan-2-ol 80:20, λ = 190 nm, *V* = 1 mL/min, *T* = 25 °C), $t_R$ = 7.3 min (*minor. enantiomer)*, $t_R$ = 8.2 min (*major enantiomer)*; HRMS (ESI+) m/z: calc. for $C_{12}H_{16}BrN_2O$ $[M+H]^+$: 283.0441, found: 283.0441.

**(*R*)-6-Bromo-2-isobutyl-2,3-dihydroquinazolin-4(1*H*)-one (3m):**

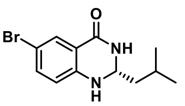

3m

The title compound **3m** was prepared according to the general procedure (reaction time: 96 hours, solvent: toluene, mobile phase (*n*-hexane/EtOAc 3:1 to 2:1), affording the title compound as light-yellow semi-solid in yield 78 % (22 mg) and 80 % *ee.* $\mathbf{R}_f$ = 0,51 (*n*-hexane/EtOAc = 1:1). **$^1$H-NMR** (400 MHz, $CDCl_3$): $\delta_H$ = 7.99 (d, *J* = 2.3 Hz, 1H), 7.37 (dd, *J* = 8.6 Hz, *J´* = 2.4 Hz, 1H), 6.57 (d, *J* = 8.6 Hz, 1H), 6.32 (s, 1H), 4.91 (t, *J* = 6.3 Hz, 1H), 4.23 (s, 1H), 1.78 (dp, *J* = 13.0 Hz, *J´* = 6.6 Hz, 1H), 1.66 (td, *J* = 7.7 Hz, *J´* = 7.1 Hz, *J´´* = 1.9 Hz, 2H), 0.99 (d, *J* = 1.3 Hz, 3H), 0.98 (d, *J* = 1.3 Hz, 3H) ppm; **$^{13}$C-NMR** (101 MHz, CDCl3) $\delta_C$ = 164.2, 146.3, 136.6, 131.3, 117.9, 116.8, 111.6, 63.7, 44.5, 24.0, 22.7 (2C) ppm; $[\boldsymbol{\alpha}]_{\mathbf{D}}^{\mathbf{20}}$ = –90,3 (*c* = 0,31; THF); **Enantiomeric excess** (80 % *e.e.*) was determined by HPLC using chiral OD-H (mobile phase: *n*-heptane/propan-2-ol 80:20, λ = 190 nm, *V* = 1 mL/min, *T* = 25 °C), $t_R$ = 9,7 min (*minor enantiomer)*, $t_R$ = 14,2 min (*major enantiomer)*; **MS** (ESI+) *m/z*: calc. for $C_{12}H_{15}BrN_2O$ $[M+H]^+$: 283.04, found: 282.93.

**(*R*)-5-Bromo-2-isobutyl-2,3-dihydroquinazolin-4(1*H*)-one (3n):**

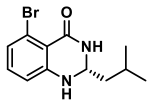

3n

The title compound **3n** was prepared according to the general procedure (reaction time: 112 hours in a toluene, mobile phase (*n*-hexane/EtOAc 2:1),

affording the title compound as white solid in yield 83 % (23 mg), m.p. 173 °C (from EtOAc), 66 % *ee*. **R*f*** = 0.15 (*n*-hexane/EtOAc 2:1). **$^{1}$H-NMR** (400 MHz, ($CDCl_3$): $\delta_H$ = 7.11 – 7.00 (m, 2H), 6.92 (s, 1H), 6.65 (dd, *J* = 7.9 Hz, *J´* = 1.1 Hz, 1H), 4.79 (t, *J* = 6.2 Hz, 1H), 4.40 (s, 1H), 1.84 (dp, *J* = 13.2 Hz, *J´* = 6.6 Hz, 1H), 1.71 (dt, *J* = 13.6 Hz, *J´* = 6.8 Hz, 1H), 1.59 (ddd, *J* = 13.7 Hz, *J´* = 7.7 Hz, *J´´* = 5.9 Hz, 1H), 0.97 (d, *J* = 4.0 Hz, 3H), 0.96 (d, *J* = 4.0 Hz, 3H) ppm; **$^{13}$C-NMR** (101 MHz, $CDCl_3$) $\delta_C$ = 163.3, 150.3, 133.3, 126.4, 123.7, 115.3, 114.9, 62.8, 43.8, 24.0, 22.9, 22.6 ppm; IR (KBr): ν = 3317, 2954, 1639, 1599, 1381, 1334 $cm^{-1}$; $[\alpha]_D^{20}$ = –93.9 (*c* = 0.33; THF); **Enantiomeric excess** (66 % *e.e.*) was determined by HPLC using chiral IA (mobile phase: *n*-heptane/propan-2-ol 80:20, λ = 190 nm, *V* = 1 mL/min, *T* = 25 °C), $t_R$ = 6,0 min (*minor enantiomer)*, $t_R$ = 6,7 min (*major enantiomer)*; **HRMS** (ESI+) *m*/*z*: calc. for $C_{12}H_{16}BrN_2O$ $[M+H]^+$: 283.0441, found: 283.0438.

**(*R*)-6-Chloro-2-isobutyl-2,3-dihydroquinazolin-4(1*H*)-one (3o):**

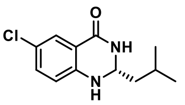

3o

The title compound **3o** was prepared according to the general procedure (reaction time: 72 hours, solvent: toluene, mobile phase (*n*-hexane/EtOAc 2:1), affording the title compound as white solid in yield 83 % (20 mg), m.p. 154-155 °C (from EtOAc), 76 % *ee.* **R*f*** = 0.39 (*n*-hexane/EtOAc 1:1, detected in vanilline). **$^{1}$H-NMR** (400 MHz, $CDCl_3$) $\delta_H$ = 7.88 – 7.78 (m, 1H), 7.23 (dd, *J* = 8.6 Hz, *J´* = 2.5 Hz, 1H), 6.88 (s, 1H), 6.62 (d, *J* = 8.6 Hz, 1H), 4.90 (t, *J* = 6.3 Hz, 1H), 4.31 (s, 1H), 1.79 (dq, *J* = 13.2 Hz, *J´* = 6.6 Hz, 1H), 1.66 (td, *J* = 6.8 Hz, *J´* = 4.8 Hz, 2H), 0.98 (s, 3H), 0.97 (s, 3H) ppm; **$^{13}$C-NMR** (101 MHz, $CDCl_3$) $\delta_C$ 164.5, 146.0, 133.7, 128.2, 124.5, 117.5, 116.5, 63.8, 44.4, 24.0, 22.8, 22.7 ppm; $[\alpha]_D^{20}$ = –116.1 (*c* = 0.28; THF); **Enantiomeric excess** (76 % *e.e.*) was determined by HPLC using chiral OD-H column (mobile phase: *n*-heptane/propan-2-ol 90:10, λ = 190 nm, *V* = 1 mL/min, *T* = 25 °C), $t_R$ = 9.2 min (*minor enantiomer)*, $t_R$ = 13.0 min (*major enantiomer)*; **MS** (ESI+) *m*/*z*: calc. for $C_{12}H_{15}ClN_2O$ $[M + Na]^+$: 261, found: 261.

**(*R*)-2-Isobutyl-7-nitro-2,3-dihydroquinazolin-4(1*H*)-one (3p):**

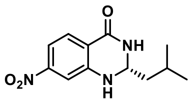

3p

The title compound **3p** was prepared according to the general procedure (reaction time: 40 hours, solvent: THF at -65 °C, mobile phase (*n*-hexane/EtOAc 2:1), affording the title compound as orange solid in yield 96 % (24 mg), m.p. 184 °C (from EtOAc), 42 % *ee.* **R*f*** = 0.37 (*n*-hexane/EtOAc 1:1, detected in vanilline). **$^{1}$H-NMR** (400 MHz, $CDCl_3$) $\delta_H$ 8.03 (d, *J* = 8.5 Hz, 1H), 7.63 (dd, *J* = 8.5 Hz, *J´* = 2.1 Hz, 1H), 7.53 (d, *J* = 2.1 Hz, 1H), 6.63 (s, 1H), 5.01 (t, *J* = 6.3 Hz, 1H), 4.61 (s, 1H), 1.83 (dt, *J* = 13.3 Hz, *J´* = 6.6 Hz, 1H), 1.71 (td, *J* = 7.6 Hz, *J´* = 7.0 Hz, *J´´* = 2.3 Hz, 2H), 1.02 (s, 3H), 1.00 (s, 2H) ppm; **$^{13}$C-NMR** (101 MHz, $CDCl_3$) $\delta_C$ = 163.5, 151.5, 147.8, 130.3, 120.5, 113.6, 109.8, 63.9, 44.7, 24.0, 22.7 (2C) ppm; $[\alpha]_D^{20}$ = –55.9 (*c* = 0.34; THF); **IR** (KBr): ν = 3512, 3067, 2944, 1745, 1329, 1269, 1159 $cm^{-1}$; **Enantiomeric excess** (36 % *e.e.*) was determined by HPLC using chiral IG column (mobile phase: *n*-heptane/propan-2-ol 80:20, λ = 254 nm, *V* = 1 mL/min, *T* = 25 °C), $t_R$ = 7.06 min (*major enantiomer)*, $t_R$ = 8.08 min (*minor enantiomer)*; **HRMS** (ESI+) *m*/*z*: calc. for $C_{12}H_{15}N_3O_3$ $[M+Na]^+$: 272.1006 found: 272.1007.

**(*R*)-2-Isobutyl-7-methyl-2,3-dihydroquinazolin-4(1*H*)-one (3q):**

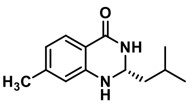

3q

The title compound **3q** was prepared according to *the general procedure* (reaction time: 84 hours, solvent: toluene, mobile phase (*n*-hexane/EtOAc 3:1 to 2:1), affording the title compound as yellow semi-solid in yield 80 % (18 mg), 69 % *ee.* **R*f*** = 0.2 (*n*-hexane/EtOAc 1:1). **$^{1}$H-NMR** (400 MHz,

$CDCl_3$) $\delta_H$ = 7.76 (d, *J* = 7.8 Hz, 1H), 6.67 (d, *J* = 7.9 Hz, 1H), 6.48 (s, 1H), 6.05 (s, 1H), 4.89 (t, J = 6.2 Hz, 1H), 4.13 (s, 1H), 2.29 (s, 3H), 1.76 (dq, *J* = 13.2 Hz, *J´* = 6.6 Hz, 1H), 1.64 (t, *J* = 6.7 Hz, 3H), 0.98 (s, 3H), 0.96 (s, 3H) ppm; **$^{13}$C-NMR** (101 MHz, $CDCl_3$) $\delta_H$ = 165.5, 147.6, 144.8, 128.8, 121.0, 115.3, 113.9, 63.8, 44.5, 24.1, 22.8, 22.7, 21.9 ppm; $[\boldsymbol{\alpha}]_{\mathbf{D}}^{\mathbf{20}}$ = –89.2 (*c* = 0.19; THF); **Enantiomeric excess** (69 % *e.e.*) was determined by HPLC using chiral IG column (mobile phase: *n*-heptane/propan-2-ol 80:20, λ = 223 nm, *V* = 1 mL/min, *T* = 25 °C), $t_R$ = 17.0 min (*minor enantiomer*), $t_R$ = 18.5 min (*major enantiomer*); **MS** (ESI+) *m*/*z*: calc. for $C_{13}H_{18}N_2O$ $[M + Na]^+$: 241, found: 241.

**(*R*)-2-Isobutyl-6-methyl-2,3-dihydroquinazolin-4(1*H*)-one (3r):**

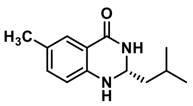

3r

The title compound **3r** was prepared according to the general procedure (reaction time: 16 hours, solvent: toluene, mobile phase (*n*-hexane/EtOAc 3:1 to 2:1), affording the title compound as white semi-solid in yield 96 % (20 mg) and 73 % *ee.* **$R_f$** = 0.18 (*n*-hexane/EtOAc 1:1, detected in vanilline). **$^1$H-NMR** (400 MHz, $CDCl_3$) $\delta_H$ = 7.69 (s, 1H), 7.12 (dd, *J* = 8.1 Hz, *J´* = 1.9 Hz, 1H), 6.60 (d, *J* = 8.1 Hz, 1H), 6.12 (s, 1H), 4.87 (t, *J* = 6.2 Hz, 1H), 4.07 (s, 1H), 2.27 (s, 3H), 1.77 (dq, *J* = 13.3 Hz, *J´* = 6.7 Hz, 1H), 1.65 (t, *J* = 6.7 Hz, 2H), 0.98 (d, *J* = 1.1 Hz, 3H), 0.97 (d, *J* = 1.1 Hz, 3H) ppm.; **$^{13}$C-NMR** (101 MHz, $CDCl_3$) $\delta_C$ = 165.6, 145.3, 134.8, 129.2, 128.6, 116.5, 115.3, 63.9, 44.4, 24.1, 22.8, 22.7, 20.6 ppm; $[\boldsymbol{\alpha}]_{\mathbf{D}}^{\mathbf{20}}$ = –111.1 (*c* = 0.27; THF); **Enantiomeric excess** (73 % *e.e.*) was determined by HPLC using chiral OD-H column (mobile phase: *n*-heptane/propan-2-ol 80:20, λ = 220 nm, *V* = 1 mL/min, *T* = 25 °C), $t_R$ = 7.2 min (*minor enantiomer*), $t_R$ = 9.3 min (*major enantiomer*); **MS** (ESI+) *m*/*z*: calc. for $C_{13}H_{18}N_2O$ $[M + Na]^+$: 241, found: 241.

**(*R*)-2-Isobutyl-6-methoxy-2,3-dihydroquinazolin-4(1*H*)-one (3s):**

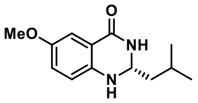

3s

The title compound **3s** was prepared according to the general procedure (reaction time: 24 hours, solvent: toluene, mobile phase (*n*-hexane/EtOAc 2:1 to 1:1), affording the title compound as white solid in yield 74 % (17 mg), m.p. 127 °C (from EtOAc), 64 % *ee.* **$R_f$** = 0.52 (*n*-hexane/EtOAc 1:3, detected in vanilline); **$^1$H-NMR** (400 MHz, $CDCl_3$): $\delta_H$ = 7.40 (d, *J* = 3.0 Hz, 1H), 6.93 (dd, *J* = 8.7 Hz, *J´* = 3.0 Hz, 1H), 6.67 (d, *J* = 8.7 Hz, 1H), 6.62 (s, 1H), 4.84 (t, *J* = 6.2 Hz, 1H), 4.00 (s, 1H), 3.78 (s, 3H), 1.80 (dp, *J* = 13.2 Hz, *J´* = 6.6 Hz, 1H), 1.65 (t, *J* = 6.7 Hz, 2H), 0.97 (d, *J* = 1.3 Hz, 3H), 0.95 (d, *J* = 1.3 Hz, 3H) ppm; **$^{13}$C-NMR** (101 MHz, $CDCl_3$) $\delta_C$ = 165.6, 153.6, 141.6, 122.4, 117.7, 117.4, 110.6, 64.0, 55.9, 44.2, 24.0, 22.8, 22.7 ppm; $[\boldsymbol{\alpha}]_{\mathbf{D}}^{\mathbf{20}}$ = –72.7 (*c* = 0.55; THF); **Enantiomeric excess** (64 % *e.e.*) was determined by HPLC using chiral IG column (mobile phase: *n*-heptane/propan-2-ol 80:20, λ = 190 nm, *V* = 1 mL/min, *T* = 25 °C), $t_R$ = 11.6 min (*minor enantiomer*), $t_R$ = 13.0 min (*major enantiomer*); **MS** (ESI+) *m*/*z*: calc. for $C_{13}H_{18}N_2O_2$ $[M + Na]^+$: 257, found: 257.

**(*R*)-2-Isobutyl-1,2,3,5-tetrahydro-4*H*-benzo[*d*][1,3]diazepin-4-one (3t)**

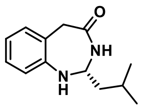

3t

The title compound **3t** was prepared according to the general procedure (reaction time: 24 hours, solvent: toluene, mobile phase (*n*-hexane/EtOAc 1:1), affording the title compound as white solid in yield 55 % (12 mg), m.p. 170-172 °C (from EtOAc), 35 % *ee.* **$^1$H-NMR** (400 MHz, $CDCl_3$): $\delta_H$ = 7.05 (t, J = 7.6 Hz, 1H), 6.97 (d, *J* = 7.6 Hz, 1H), 6.72 (td, *J* = 7.5 Hz, *J´* = 1.1 Hz, 1H), 6.53 (dd, *J* = 8.0 Hz, *J´* = 1.0 Hz, 1H), 6.13 (d, *J* = 7.2 Hz, 1H), 5.21 (p, *J* = 6.9 Hz, 1H), 4.56 (d, *J* = 15.1 Hz, 1H), 3.99 (d, *J* = 6.9 Hz, 1H), 3.29 (dd, *J* = 15.1 Hz, *J´* = 1.7 Hz, 1H), 1.80 (dp, *J* = 13.4 Hz, *J´* = 6.7 Hz, 1H), 1.56 (t, *J* = 7.0 Hz, 2H), 0.99 (s, 3H), 0.97 (s, 3H) ppm; **$^{13}$C-NMR** (101 MHz,

$CDCl_3$) $\delta_C$ = 172.8, 144.0, 132.3, 128.4, 119.7, 117.7, 116.1, 61.1, 43.8, 42.4, 24.7, 22.6, 22.5 ppm; $[\alpha]_D^{20}$ = –26.0 (*c* = 0.25; THF); **IR** (KBr): ν = 3305, 3192, 2960,1654, 1495 $cm^{-1}$; **Enantiomeric excess** (35 % *e.e.*) was determined by HPLC using chiral IA column (mobile phase: *n*-heptane/propan-2-ol 80:20, λ = 190 nm, *V* = 1 mL/min, *T* = 25 °C), $t_R$ = 7.0 min (*major enantiomer)*, $t_R$ = 10.7 min (*minor enantiomer)*; **HRMS** (ESI+) *m*/*z*: calc. for $C_{13}H_{19}N_2O$ $[M+Na]^+$: 219.1491 found: 219.1488.

# NMR spectra

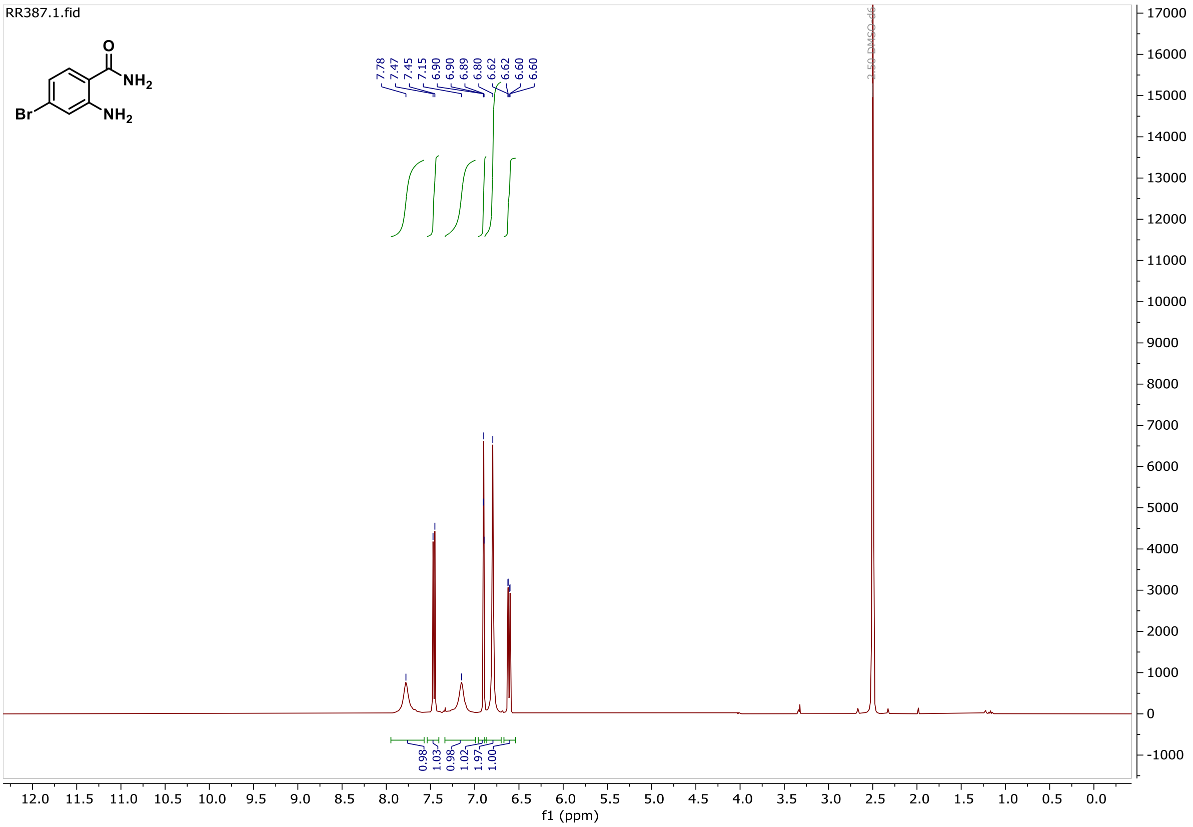

$^{1}$H NMR (400 MHz, DMSO) of **1l**.

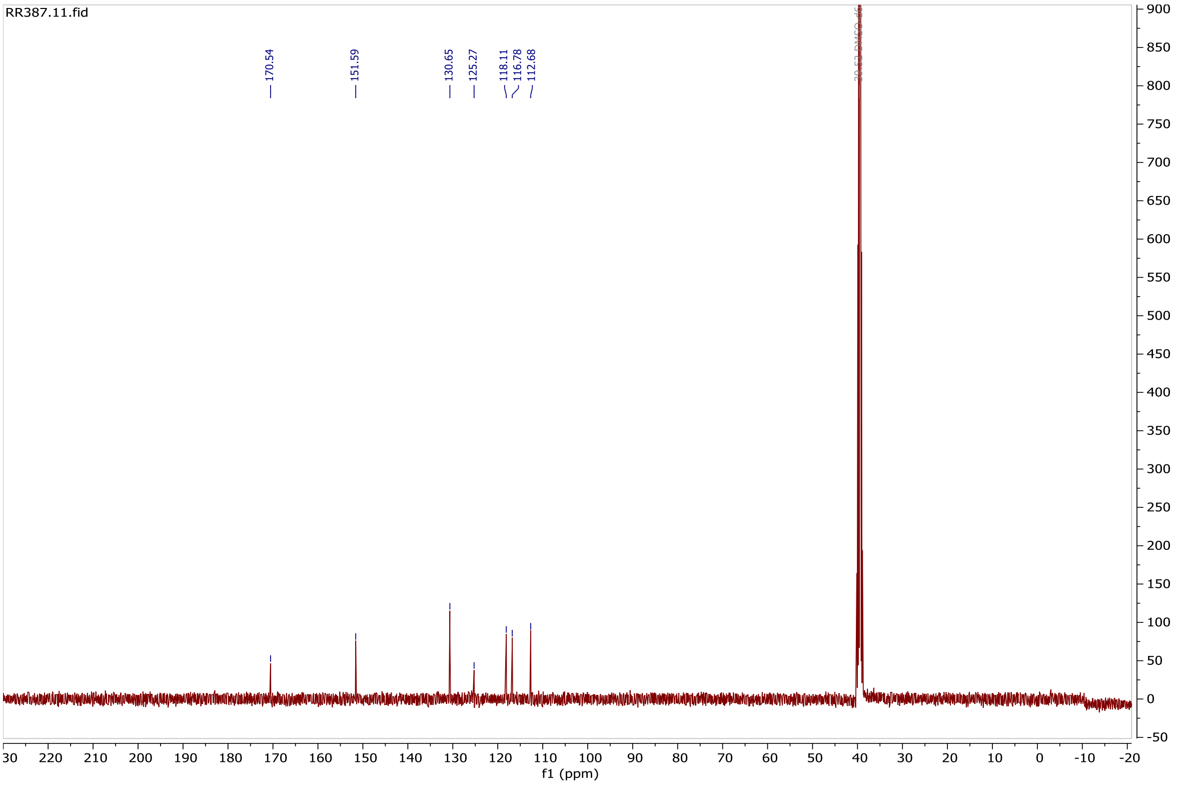

$^{13}$C{$^{1}$H} NMR (101 MHz, DMSO) of **1l**.

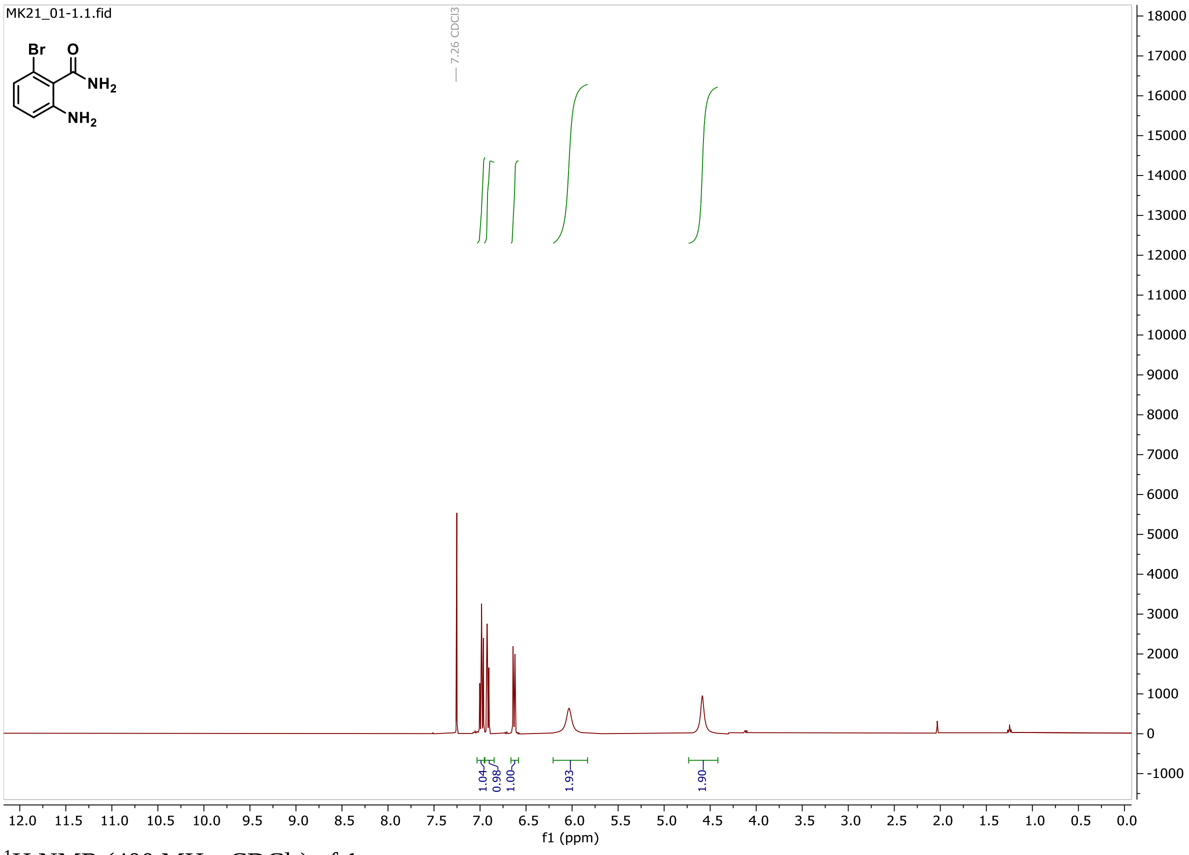


$^{1}H$ NMR (400 MHz, $CDCl_3$) of **1n**.

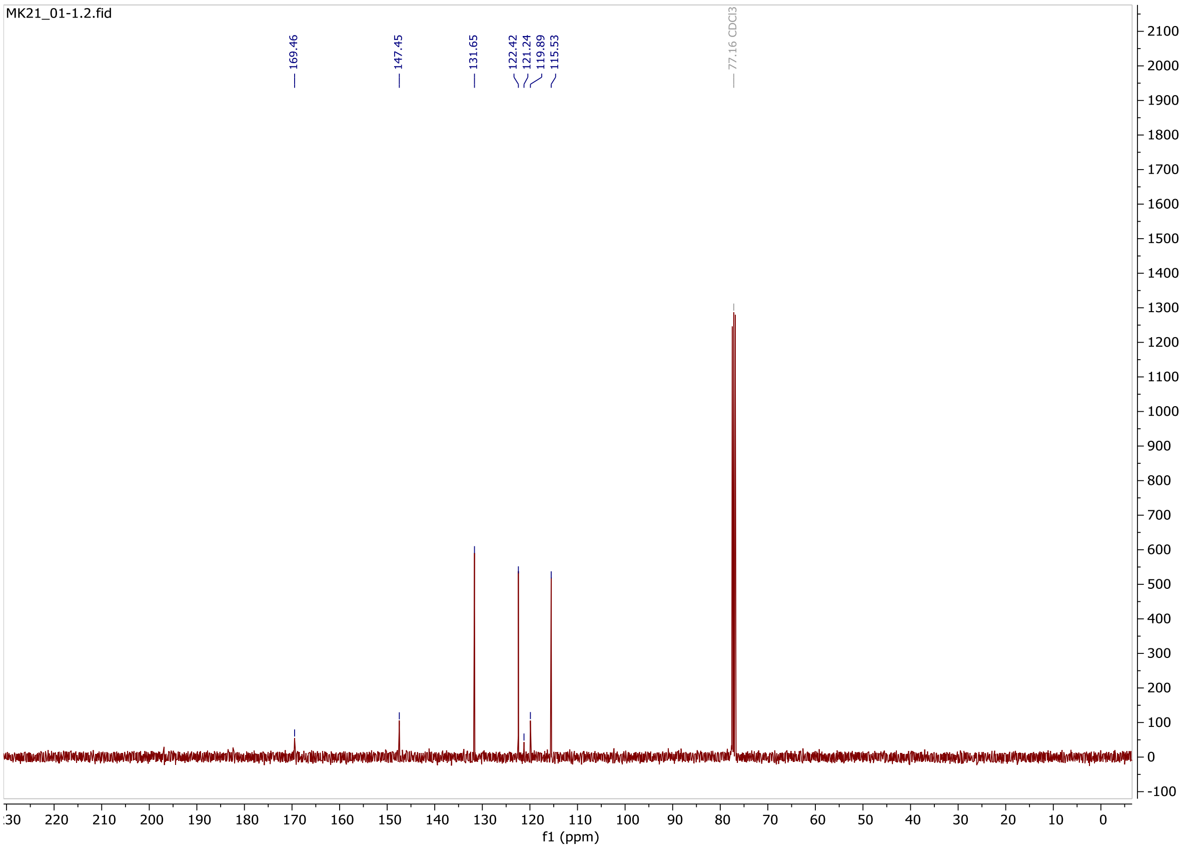


$^{13}C\{^{1}H\}$ NMR (101 MHz, $CDCl_3$) of **1n**.

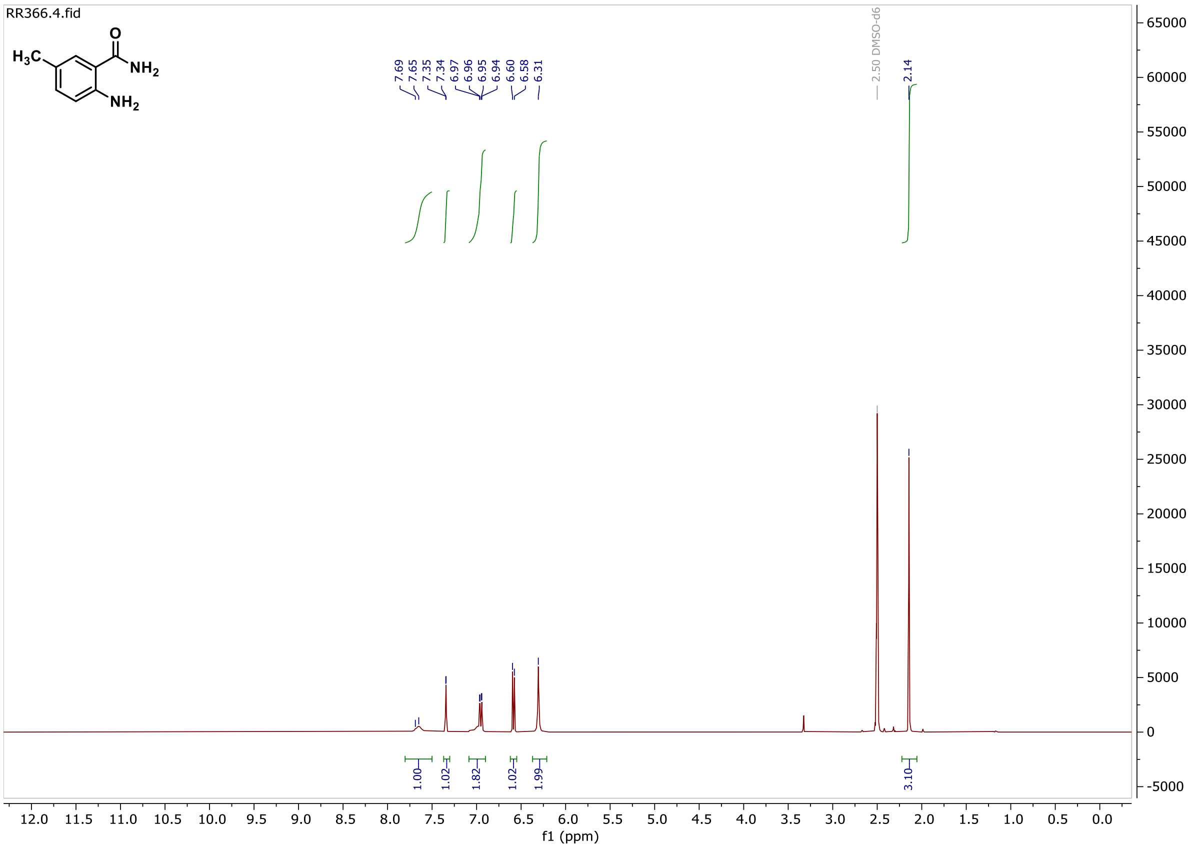

$^{1}H$ NMR (400 MHz, DMSO) of **1r**.

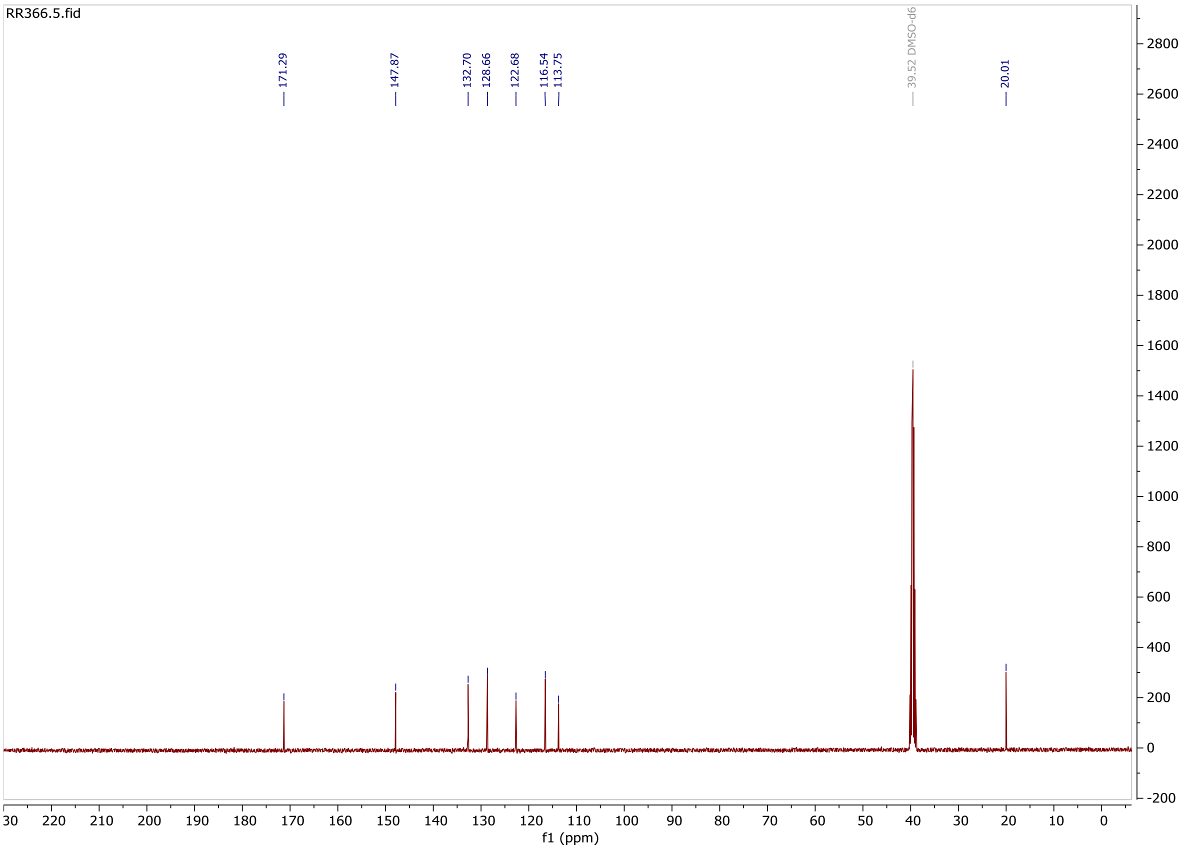

$^{13}C\{^{1}H\}$ NMR (101 MHz, DMSO) of **1r**.

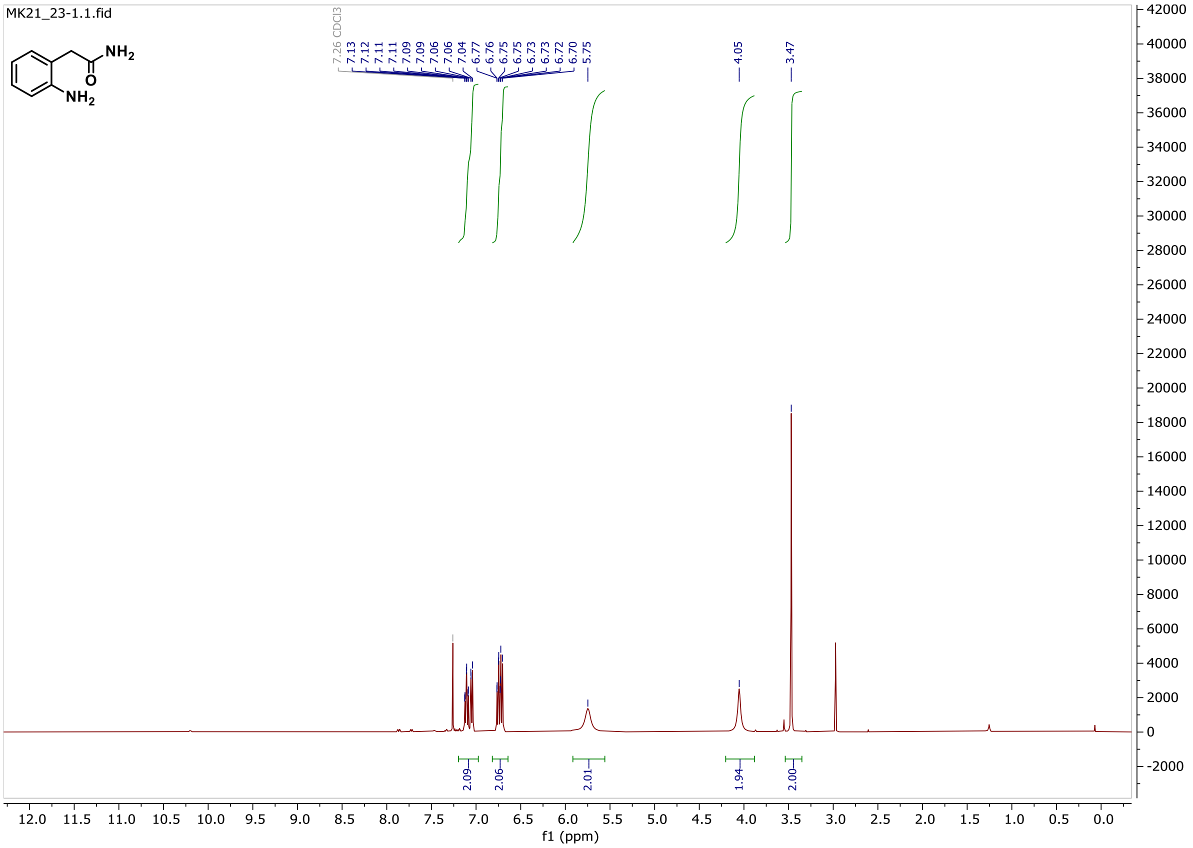


$^{1}$H NMR (400 MHz, CDCl$_3$) of **1t**.

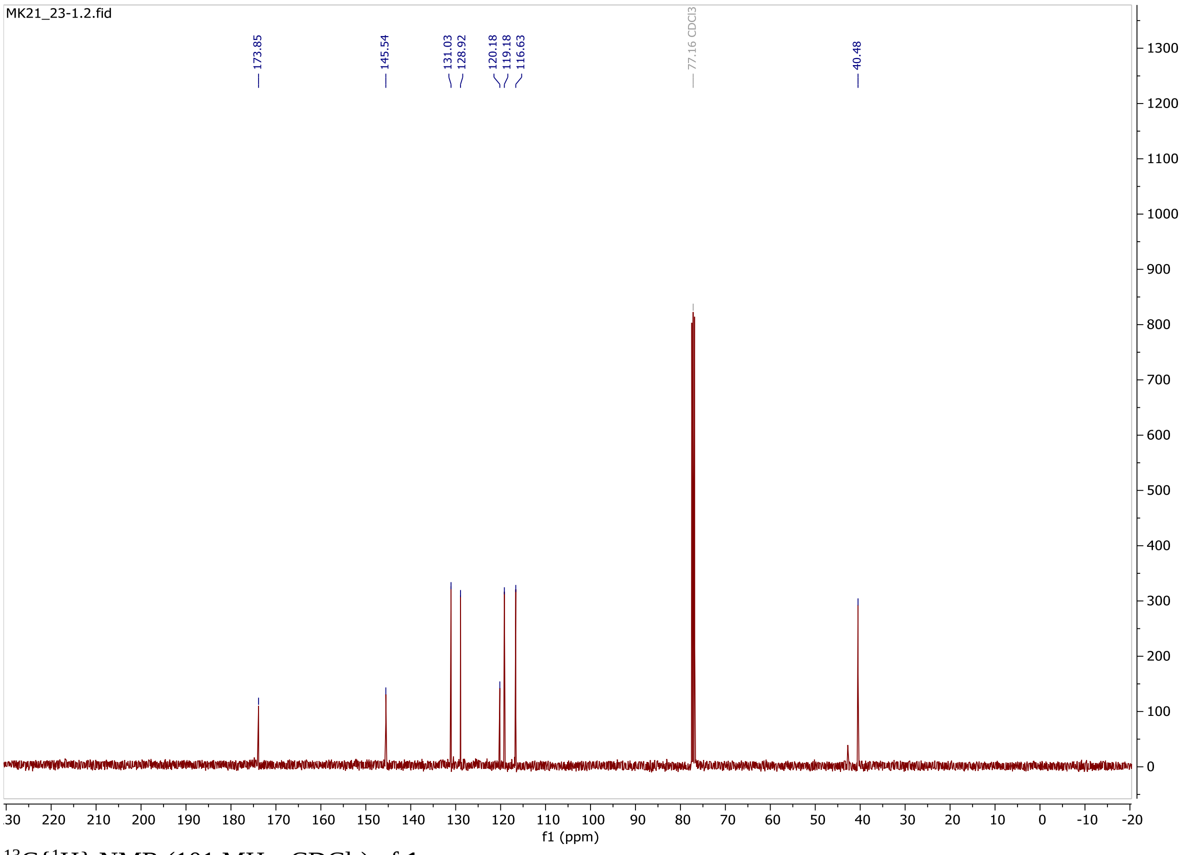


$^{13}$C{$^{1}$H} NMR (101 MHz, CDCl$_3$) of **1t**.

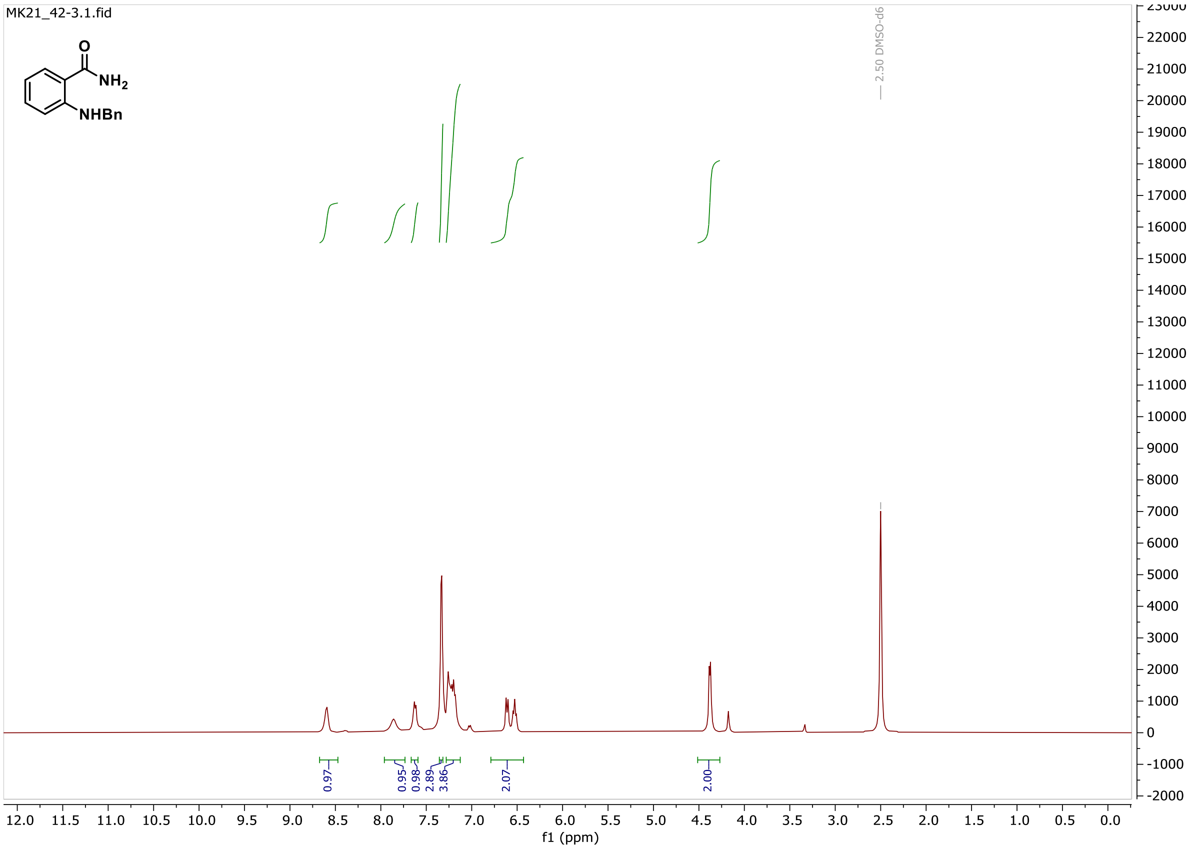


$^{1}$H NMR (400 MHz, DMSO) of **1u**.

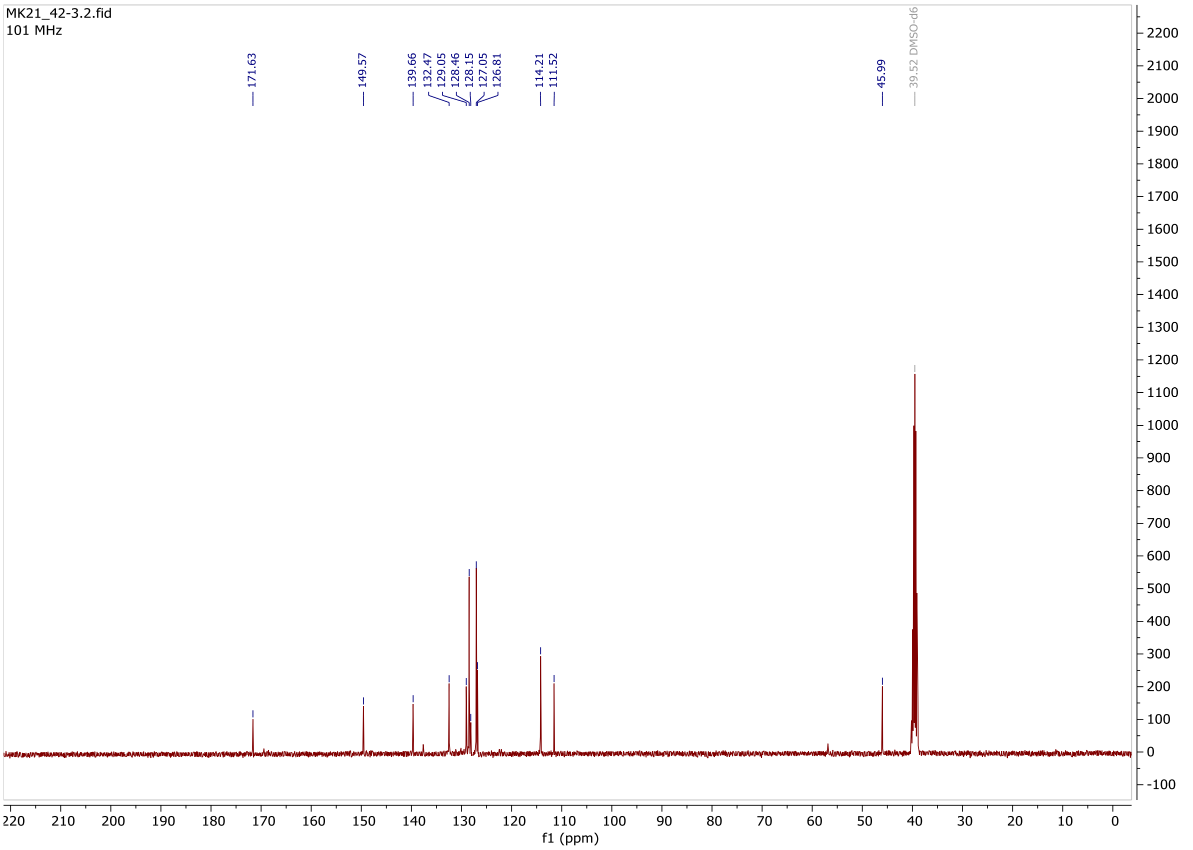


$^{13}$C{$^{1}$H} NMR (101 MHz, DMSO) of **1u**.

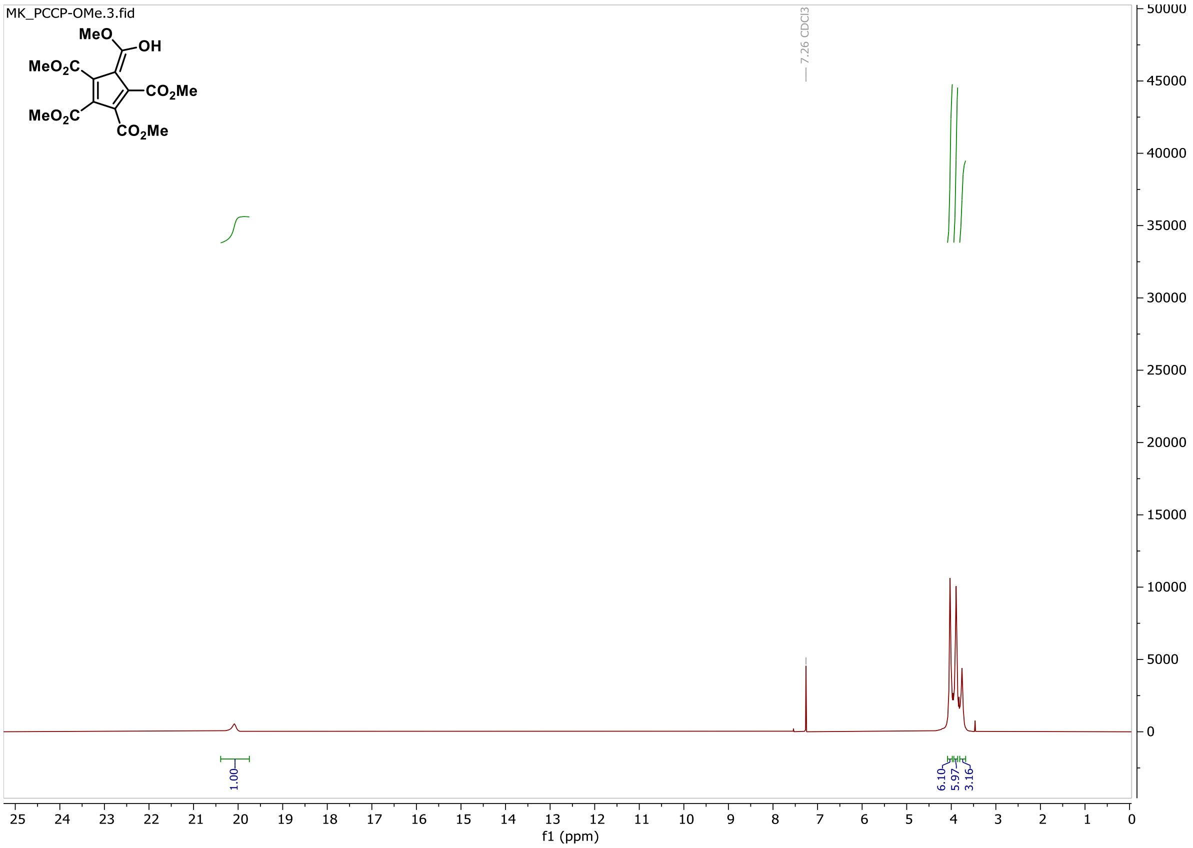


$^{1}H$ NMR (400 MHz, $CDCl_3$) of **I**.

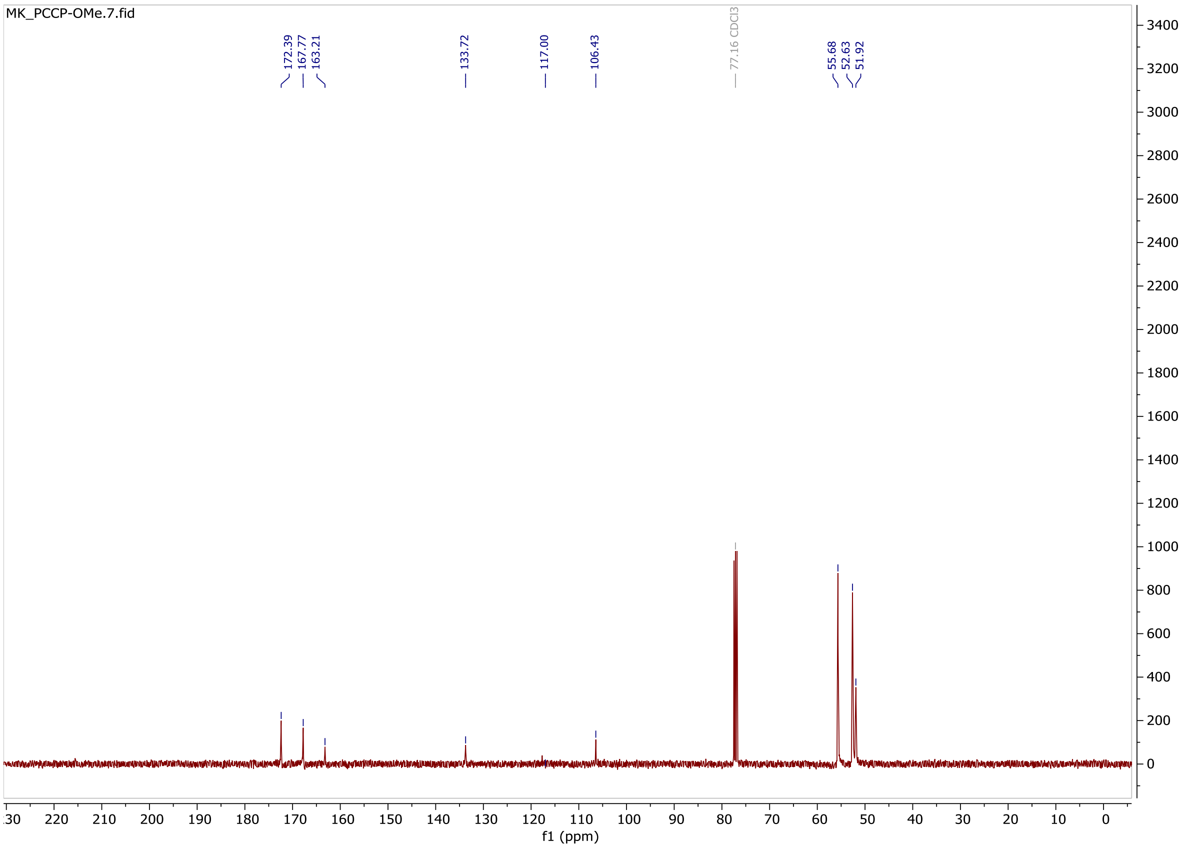


$^{13}C\{^{1}H\}$ NMR (101 MHz, $CDCl_3$) of **I**.

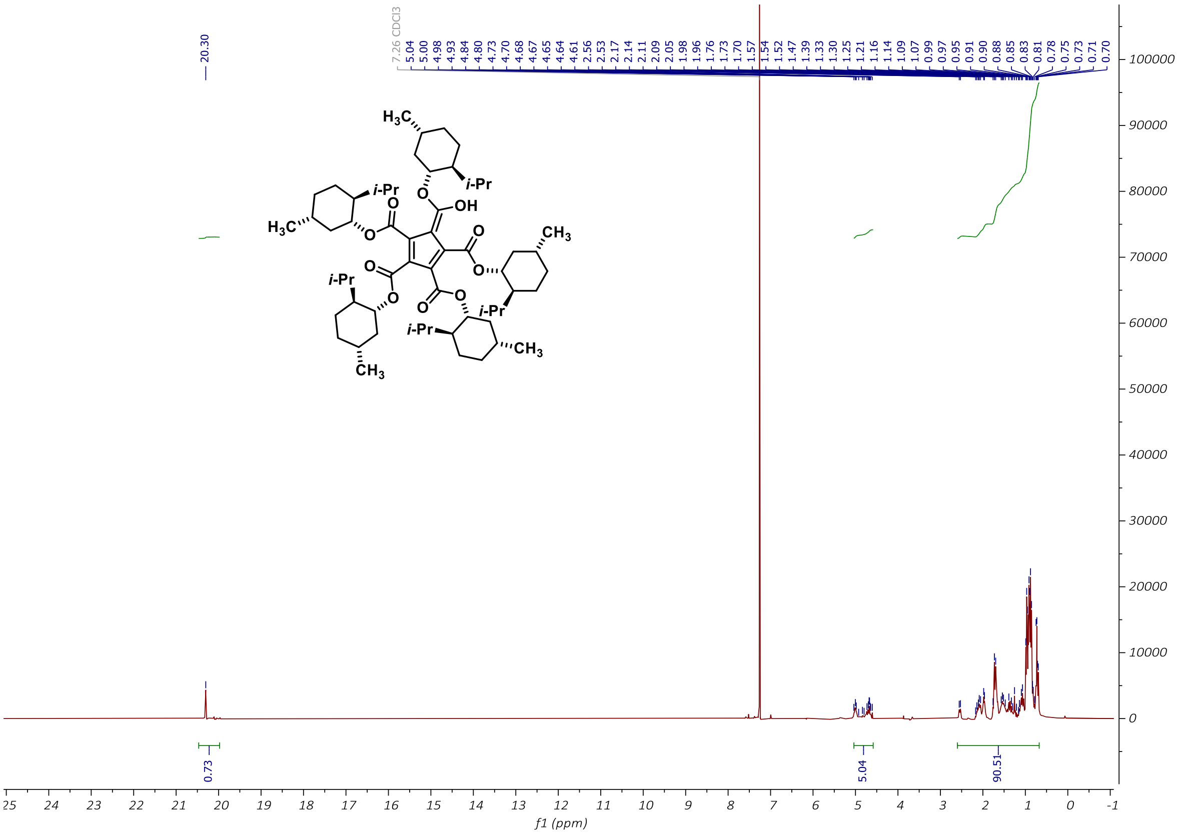

$^{1}$H NMR (400 MHz, $CDCl_3$) of **II**.

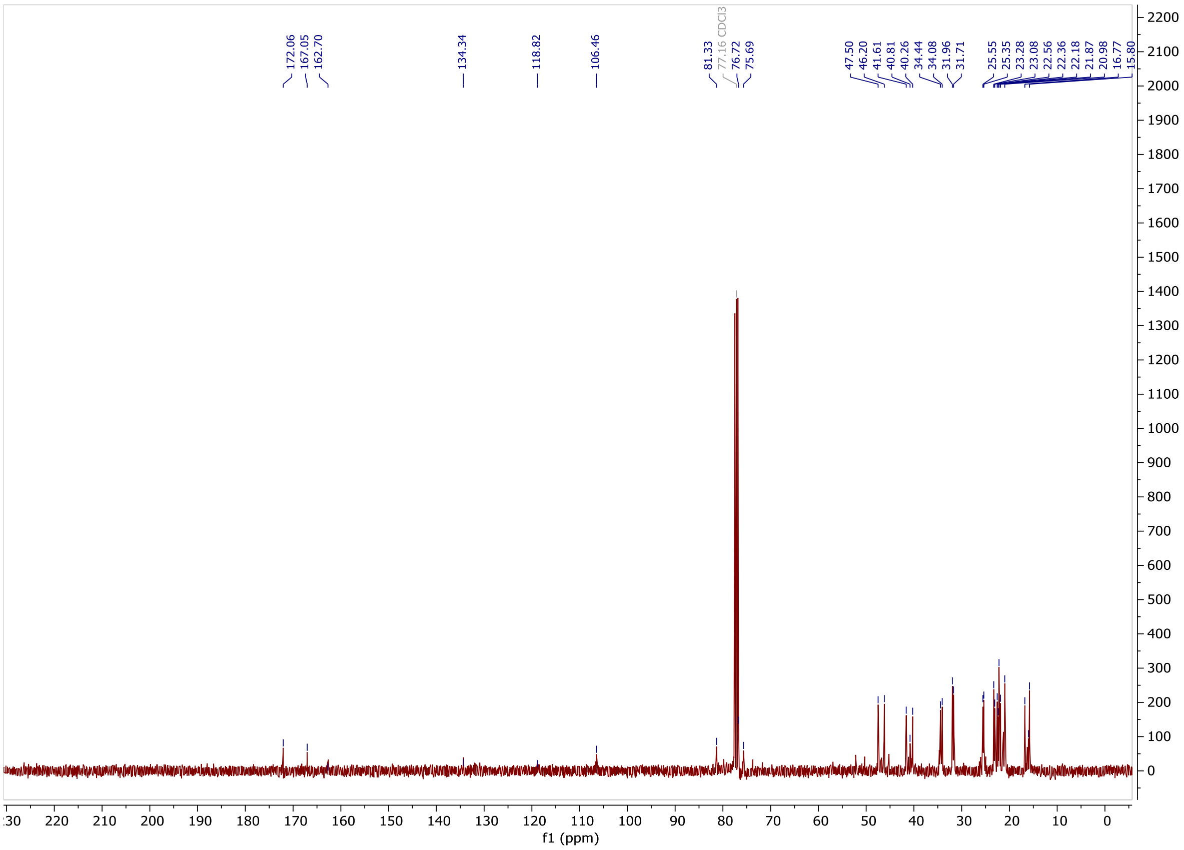

$^{13}$C{$^{1}$H} NMR (101 MHz, $CDCl_3$) of **II**.

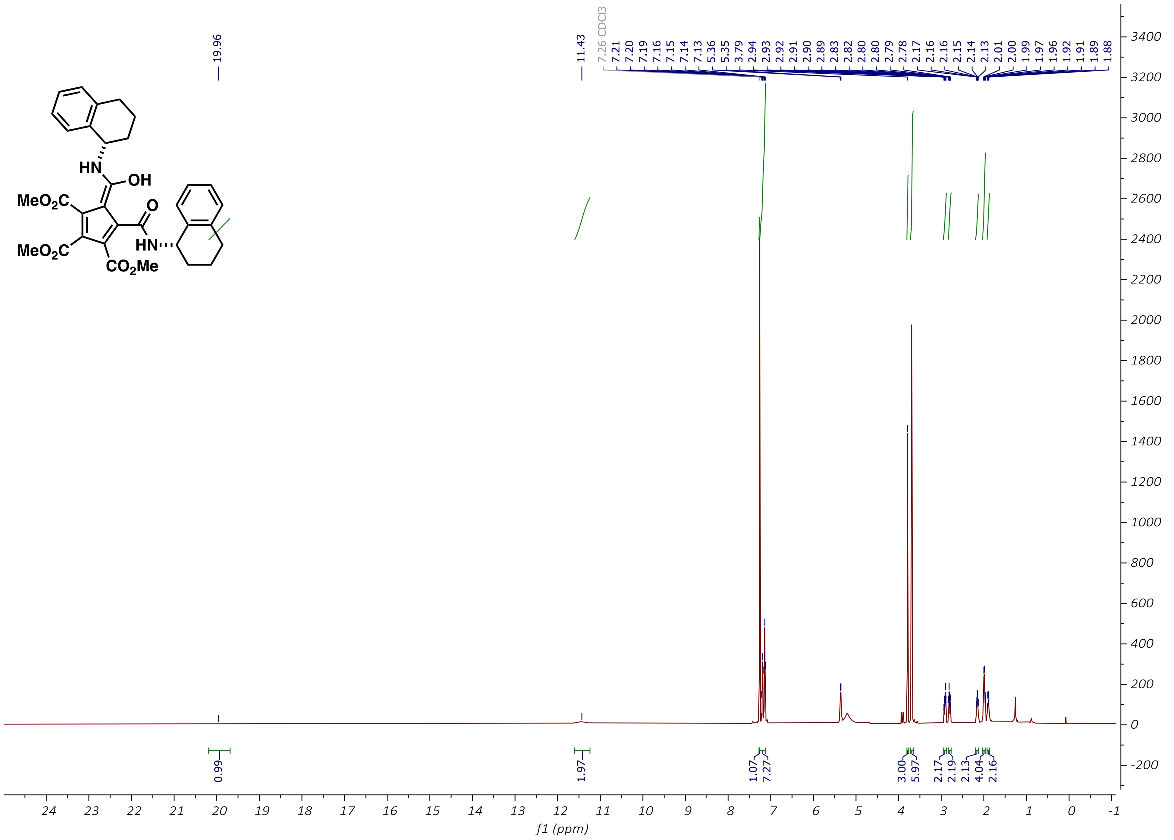

$^1H$ NMR (400 MHz, $CDCl_3$) of **III**.

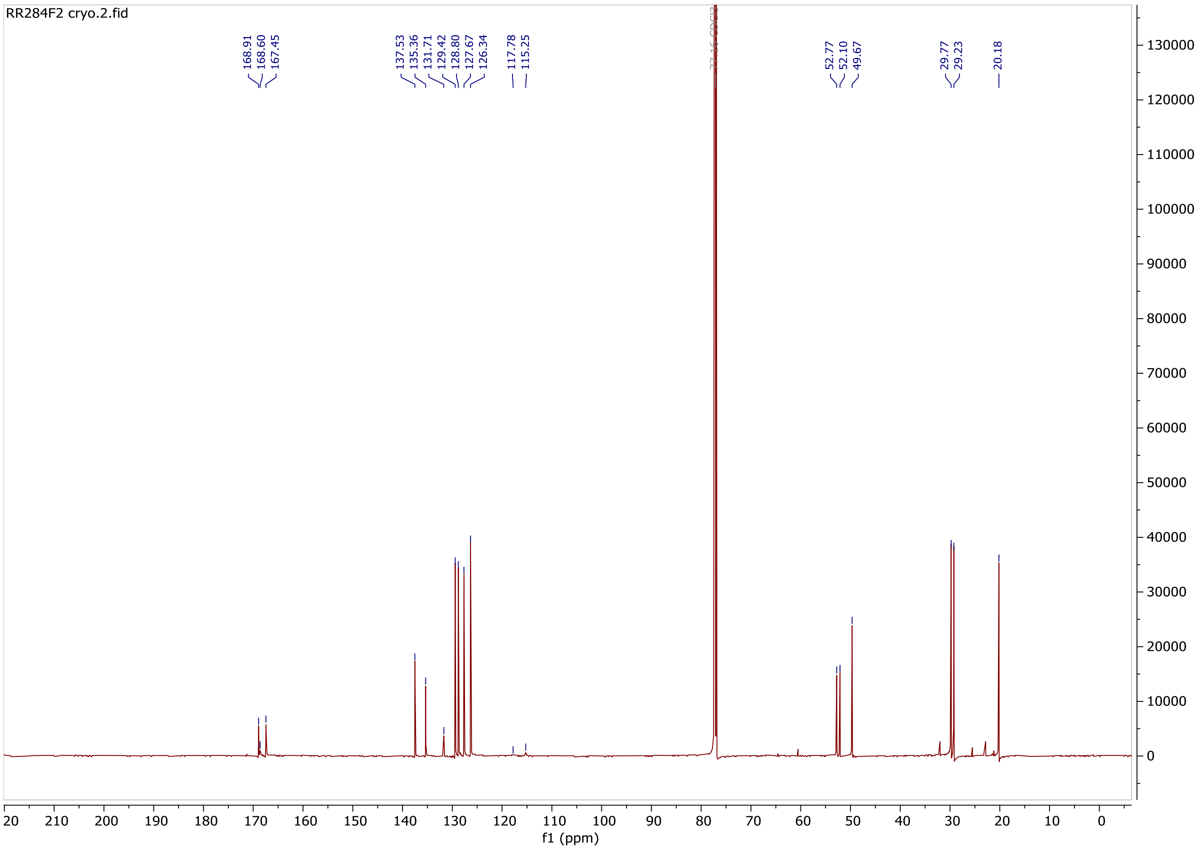

$^{13}C\{^1H\}$ NMR (101 MHz, $CDCl_3$) of **III**.

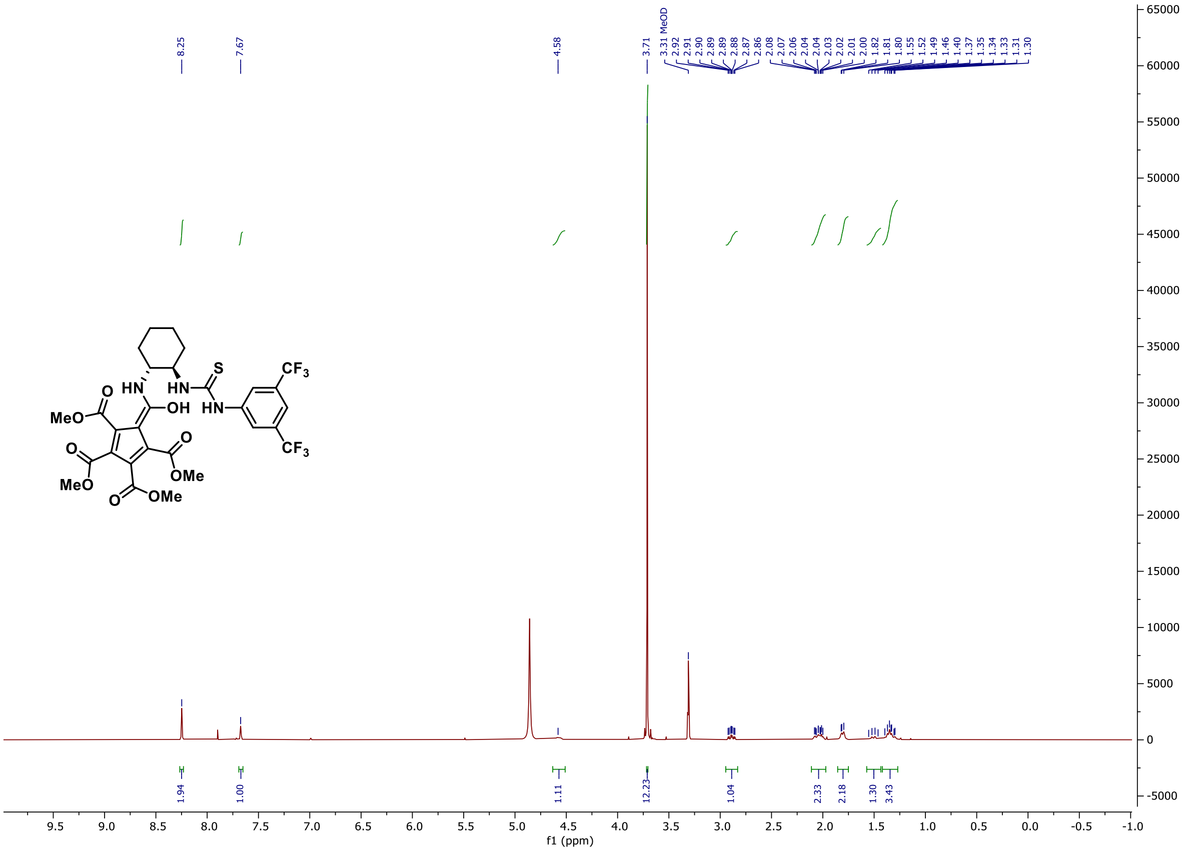


$^{1}H$ NMR (400 MHz, MeOD) of **IV**.

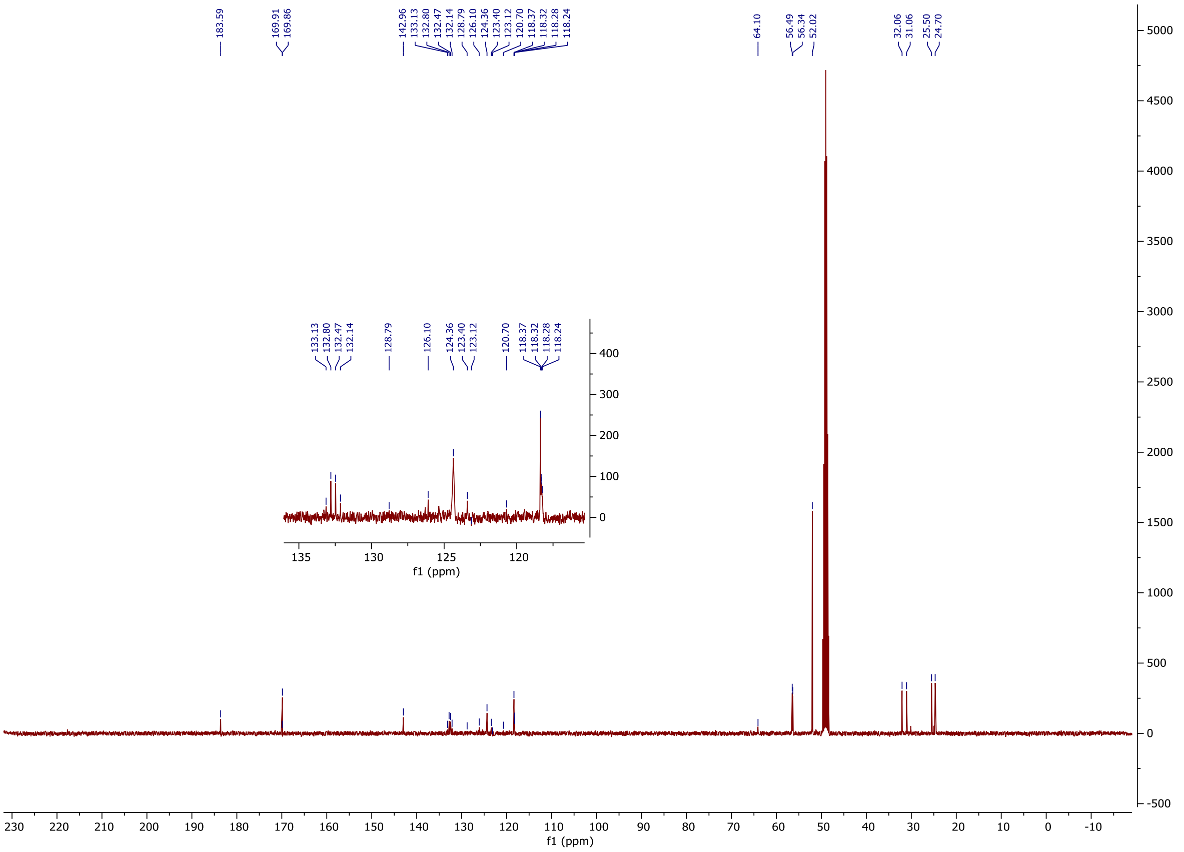


$^{13}C\{^{1}H\}$ NMR (101 MHz, MeOD) of **IV**.

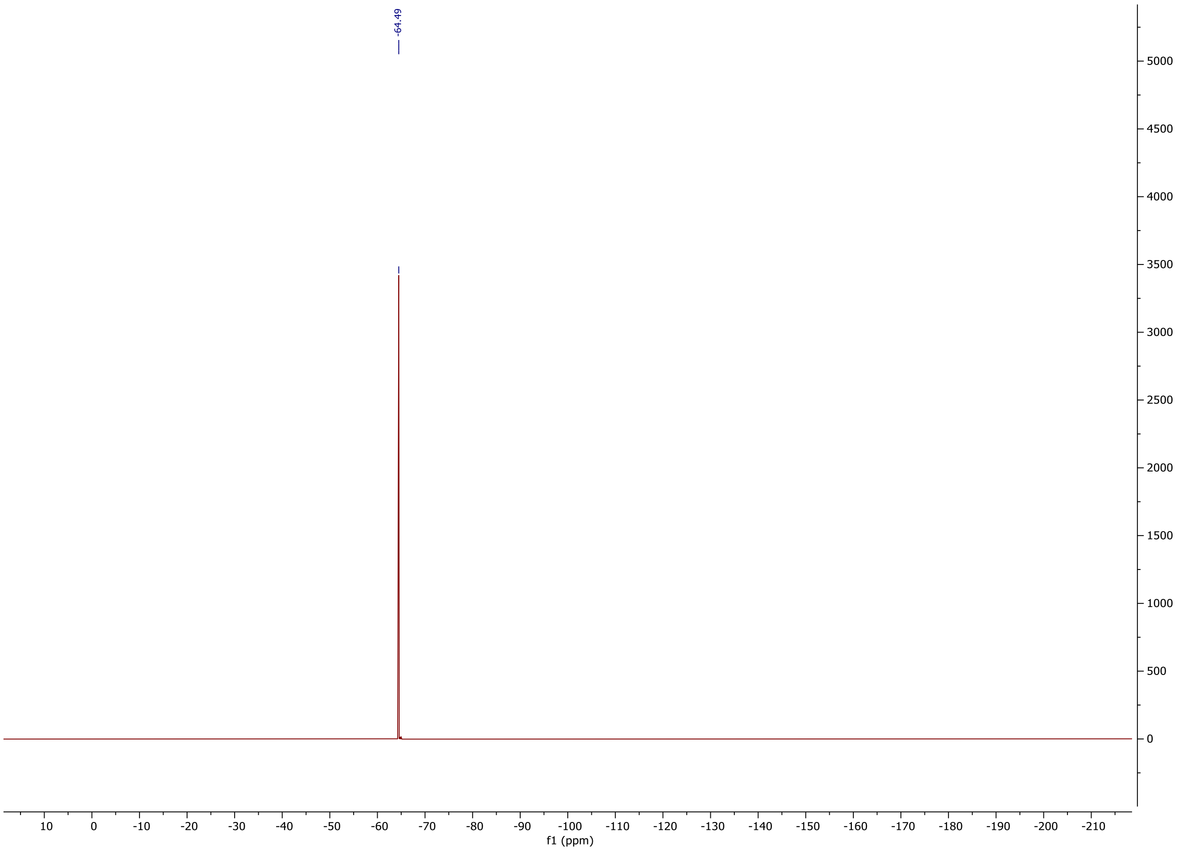

$^{19}F$ NMR (376 MHz, MeOD) of **IV**.

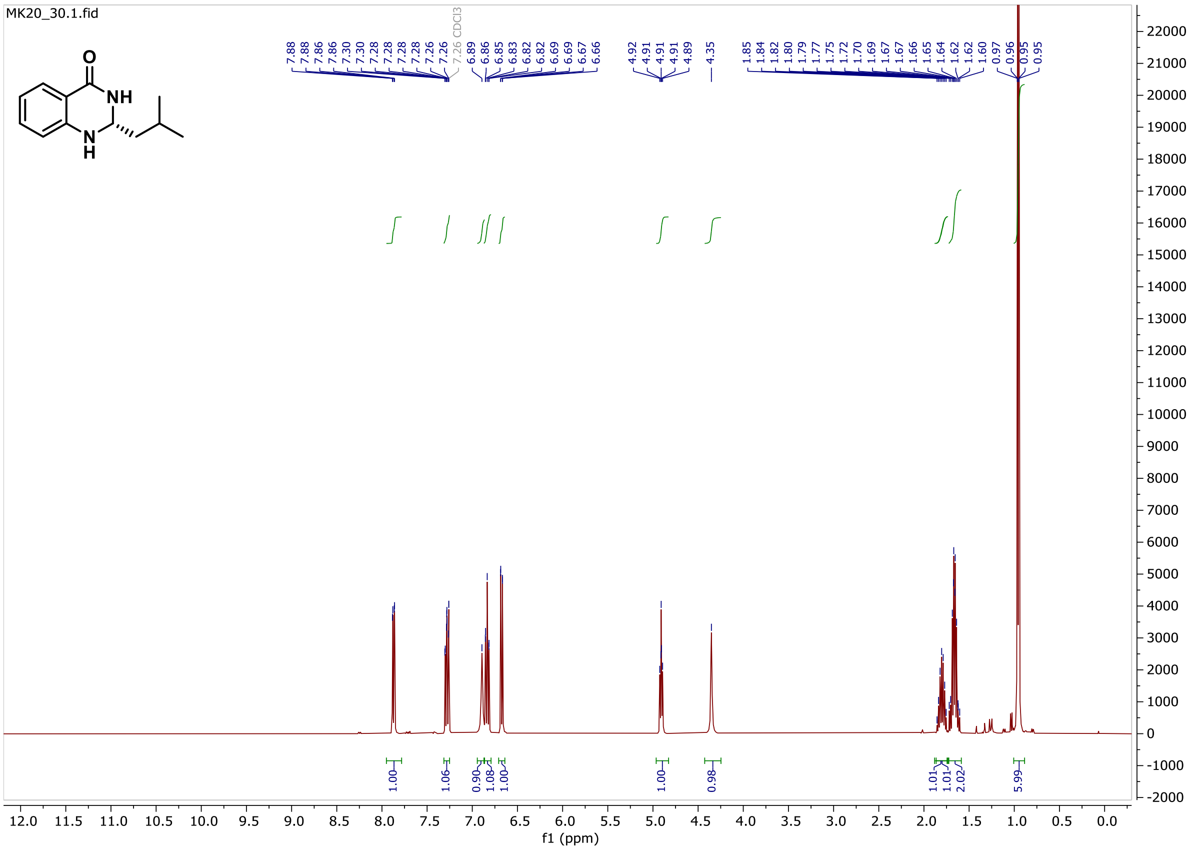

$^{1}H$ NMR (400 MHz, $CDCl_3$) of **3a**.

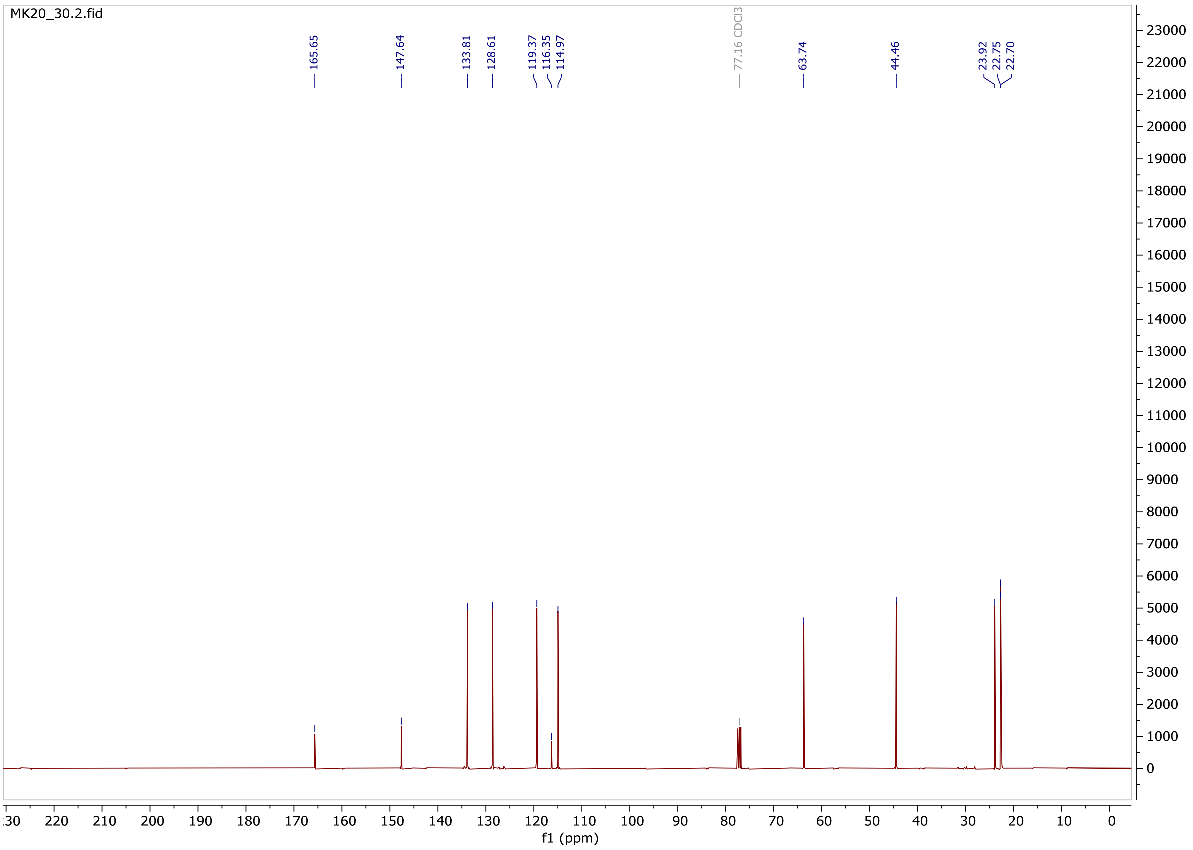

$^{13}C\{^{1}H\}$ NMR (101 MHz, $CDCl_3$) of **3a**.

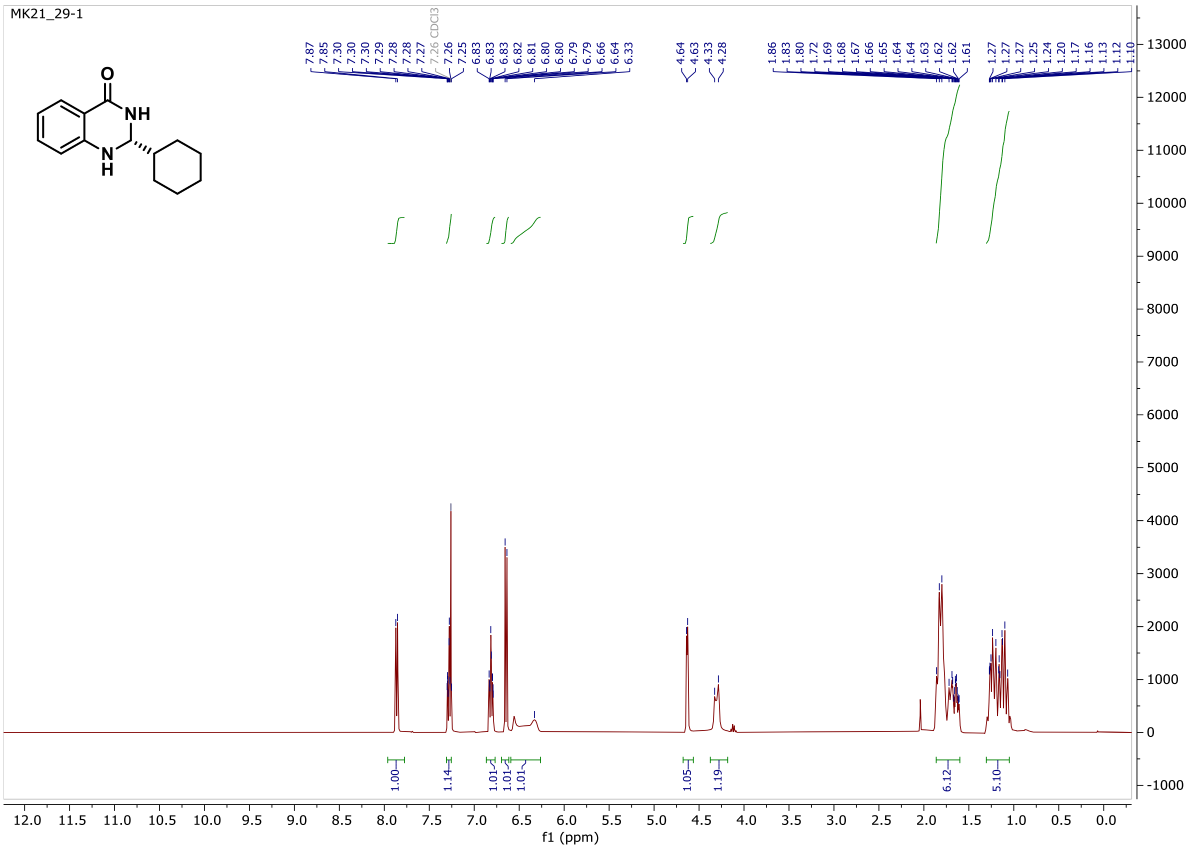


$^{1}$H NMR (400 MHz, $CDCl_3$) of **3b**.

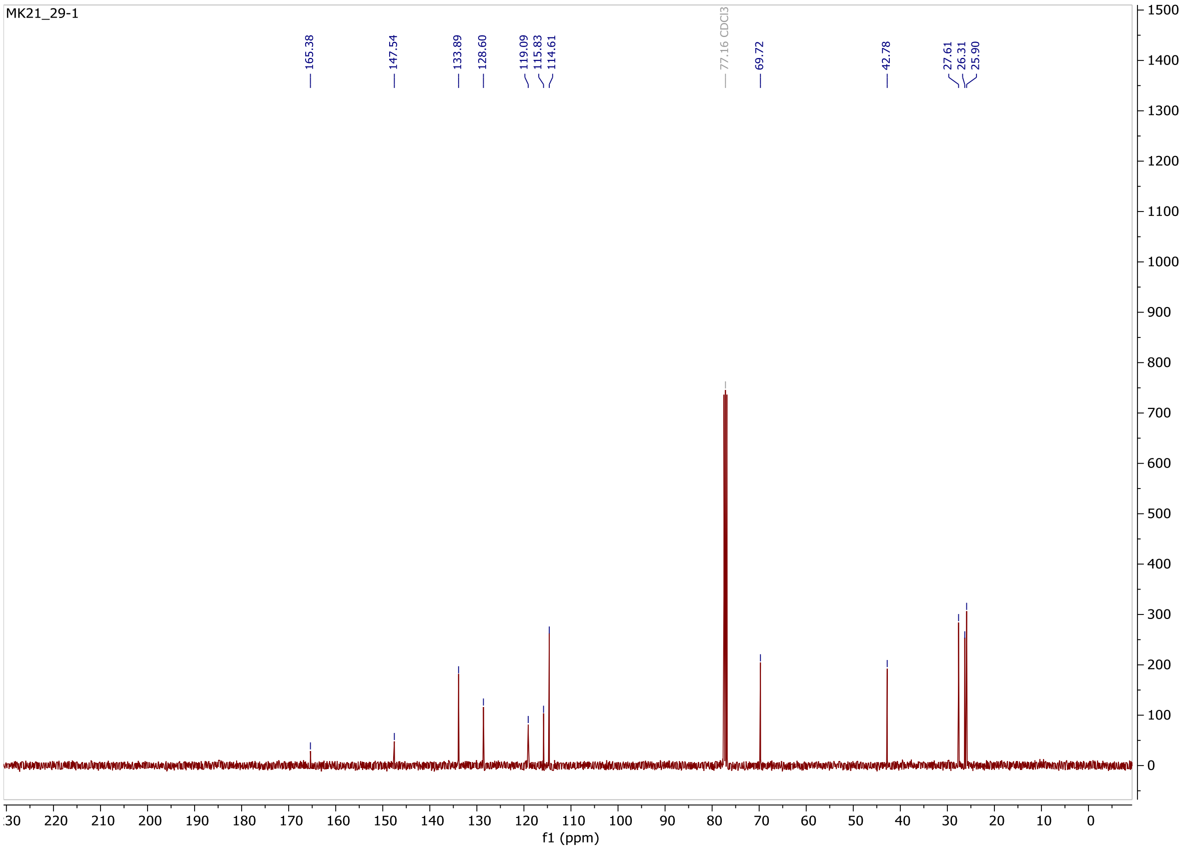


$^{13}$C{$^{1}$H} NMR (101 MHz, $CDCl_3$) of **3b**.

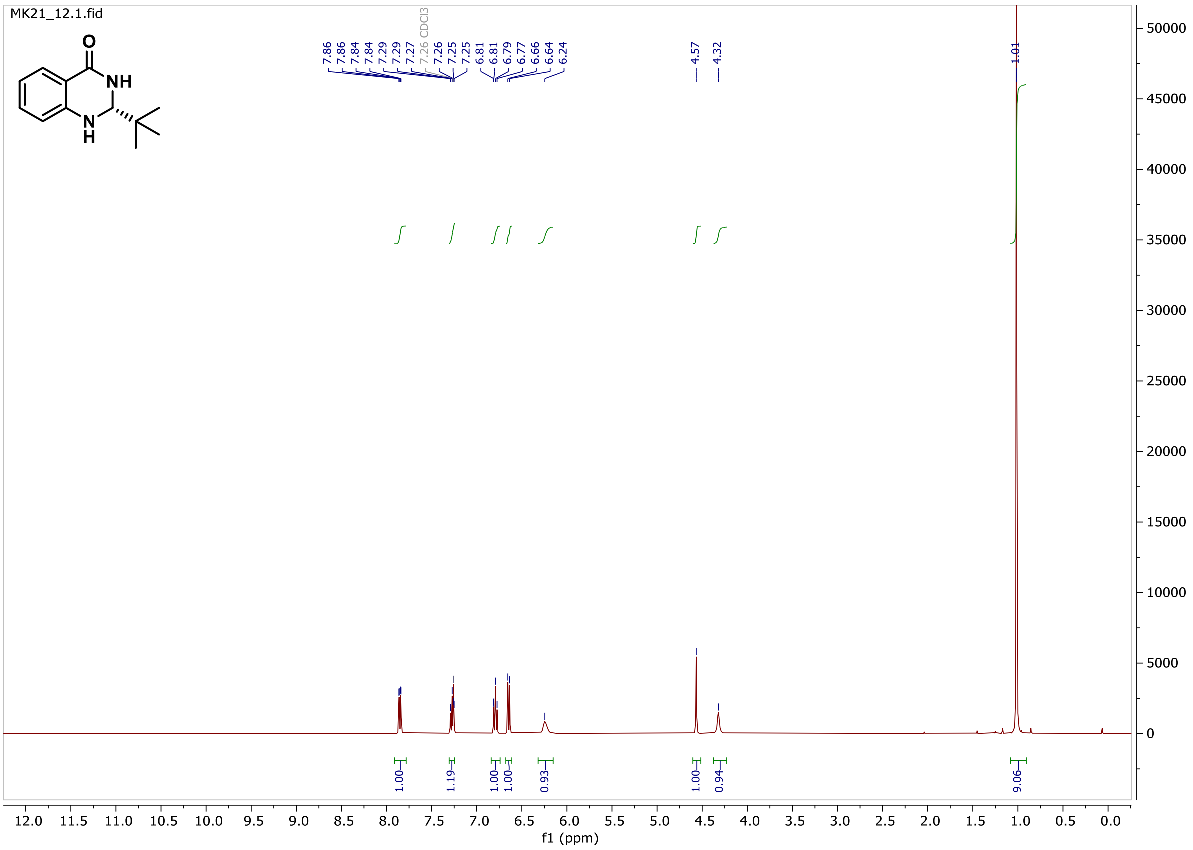

$^{1}$H NMR (400 MHz, $CDCl_3$) of **3c**.

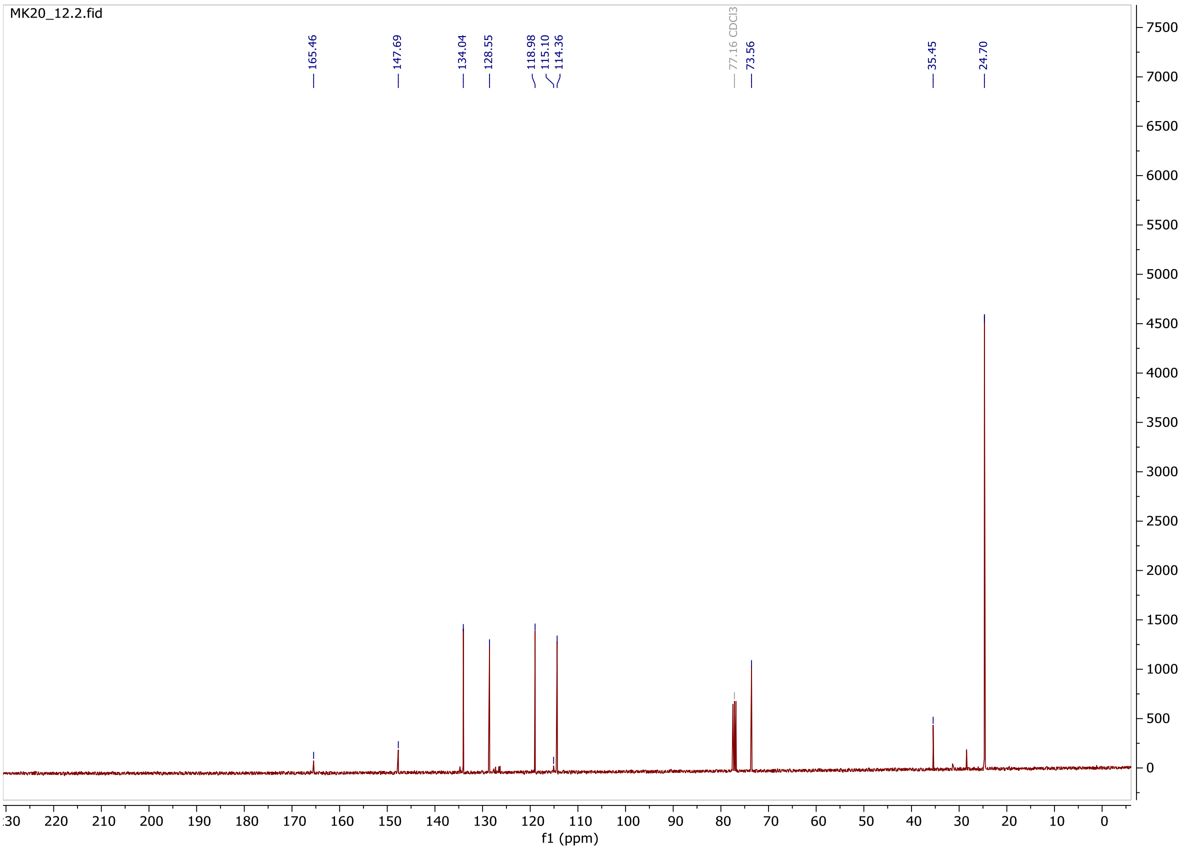

$^{13}$C{$^{1}$H} NMR (101 MHz, $CDCl_3$) of **3c**.

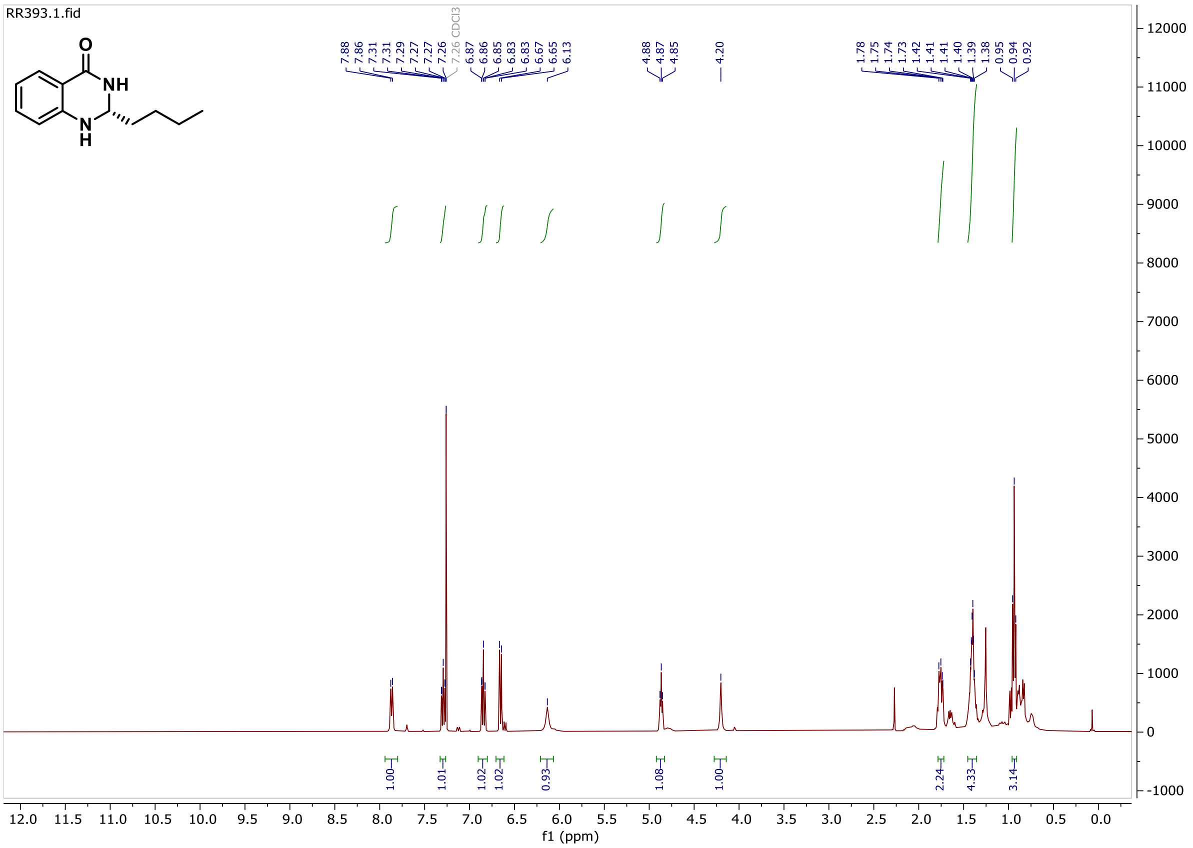

$^{1}H$ NMR (400 MHz, $CDCl_3$) of **3d**.

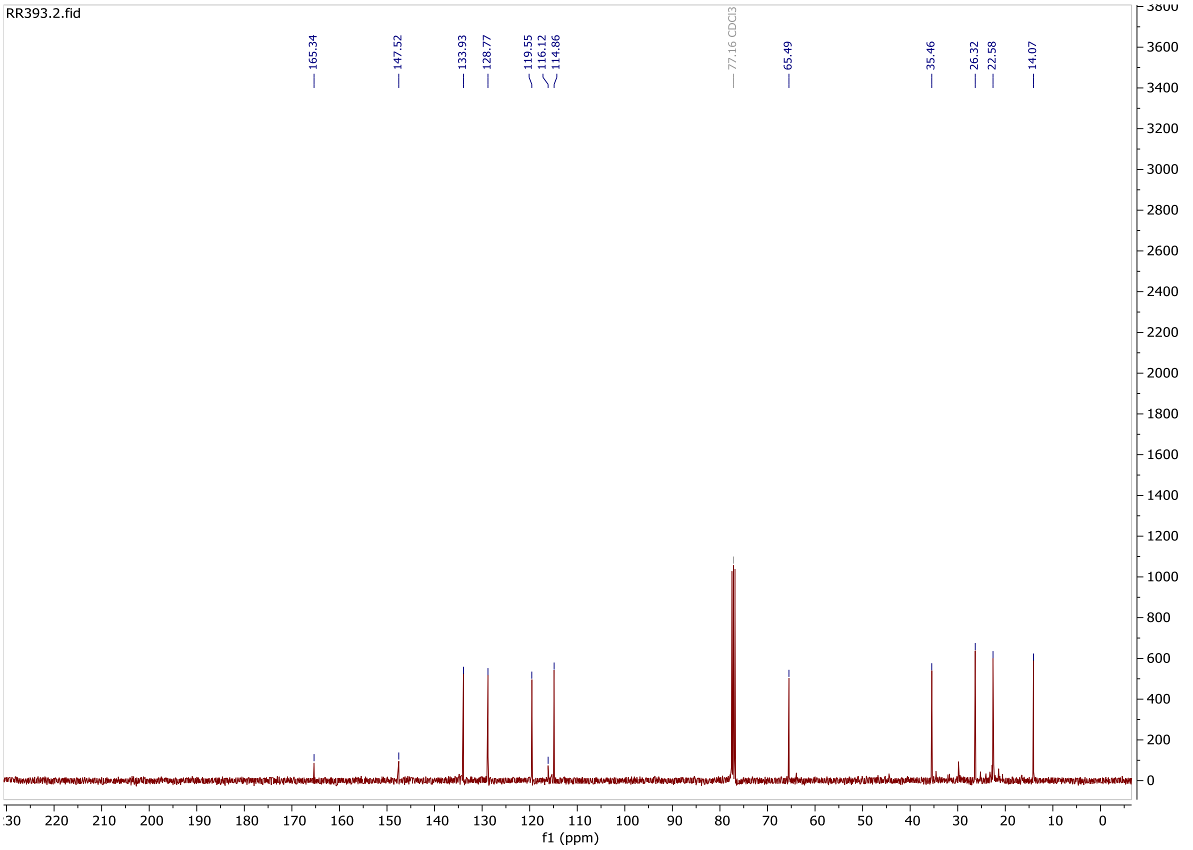

$^{13}C\{^{1}H\}$ NMR (101 MHz, $CDCl_3$) of **3d**.

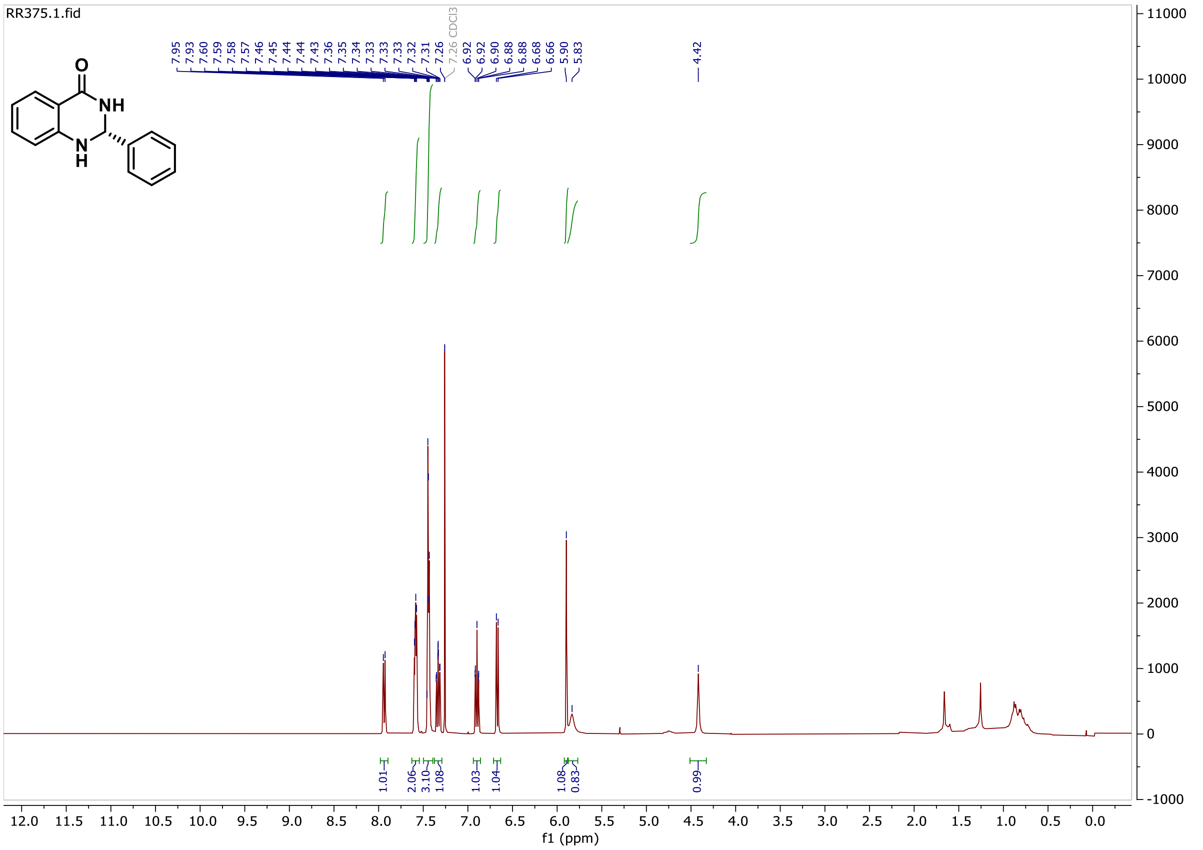

$^{1}$H NMR (400 MHz, $CDCl_3$) of **3e**.

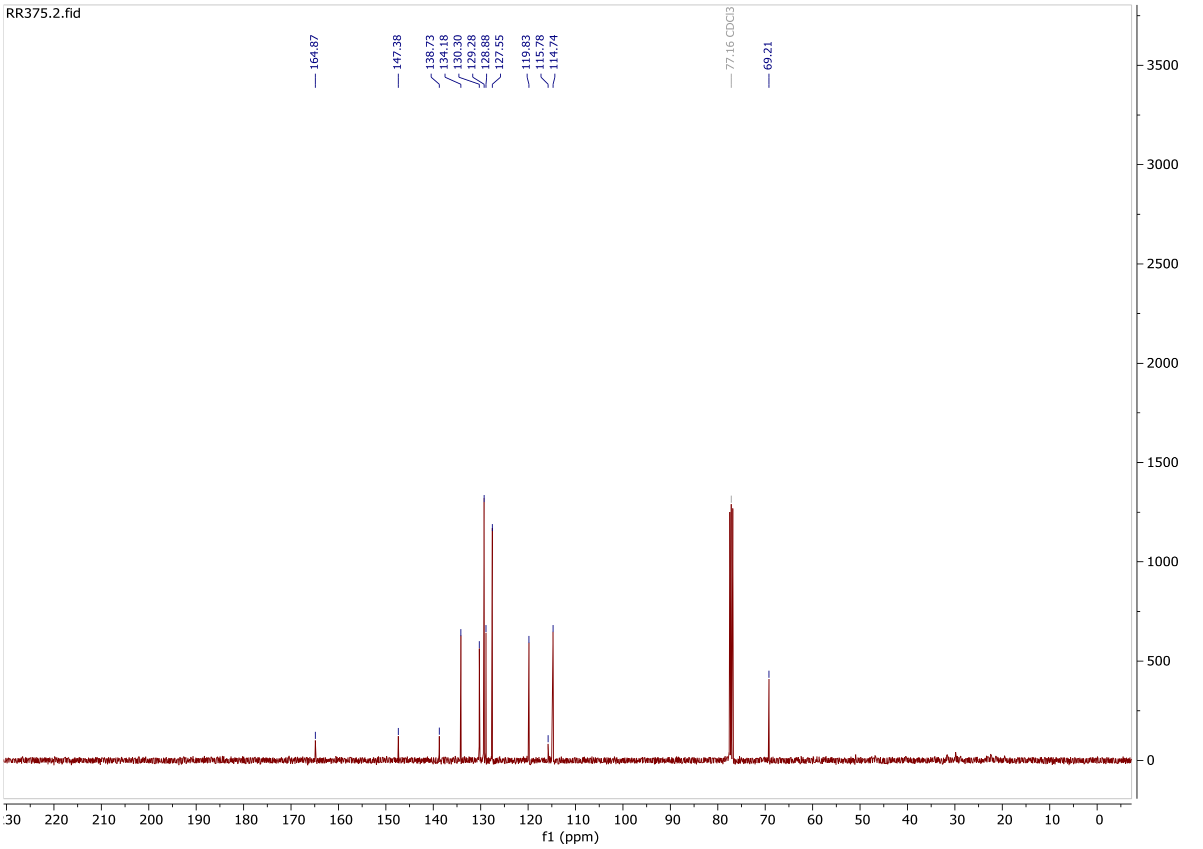

$^{13}$C{$^{1}$H} NMR (101 MHz, $CDCl_3$) of **3e**.

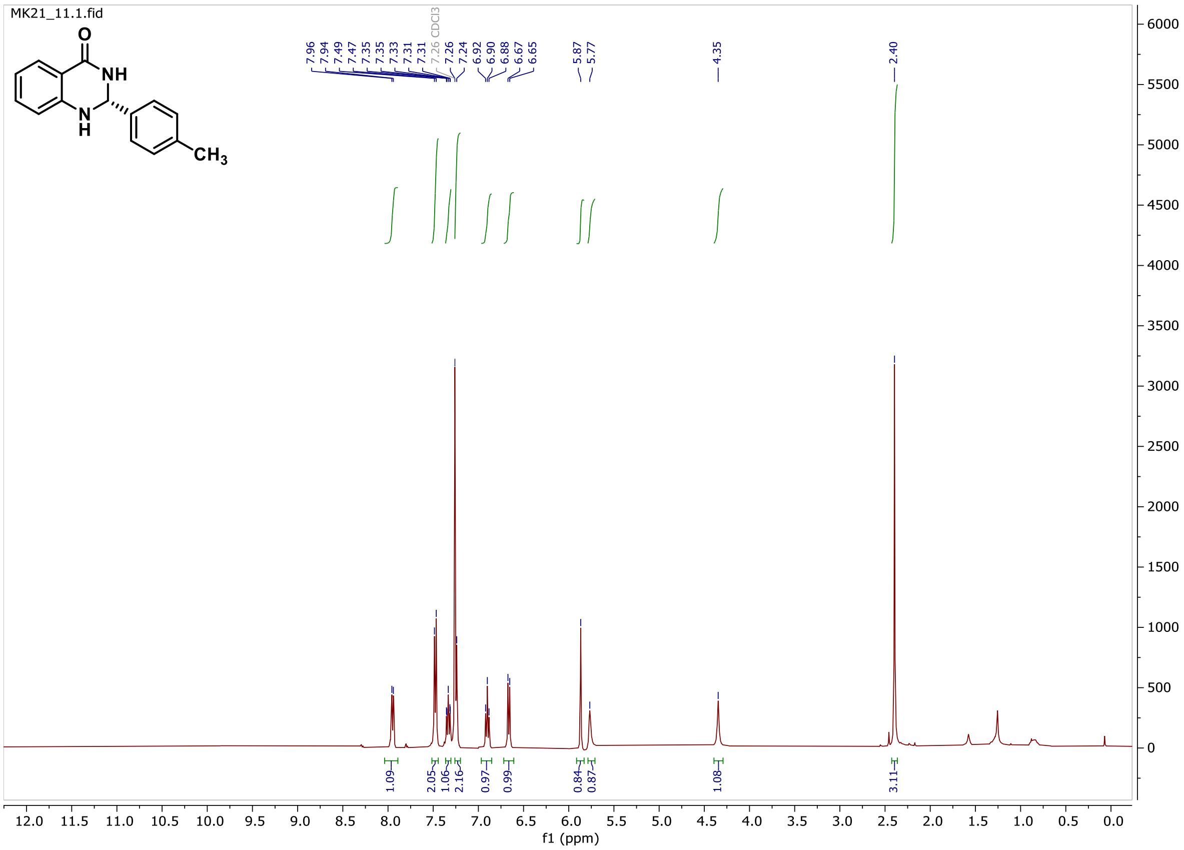

$^{1}$H NMR (400 MHz, $CDCl_3$) of **3f**.

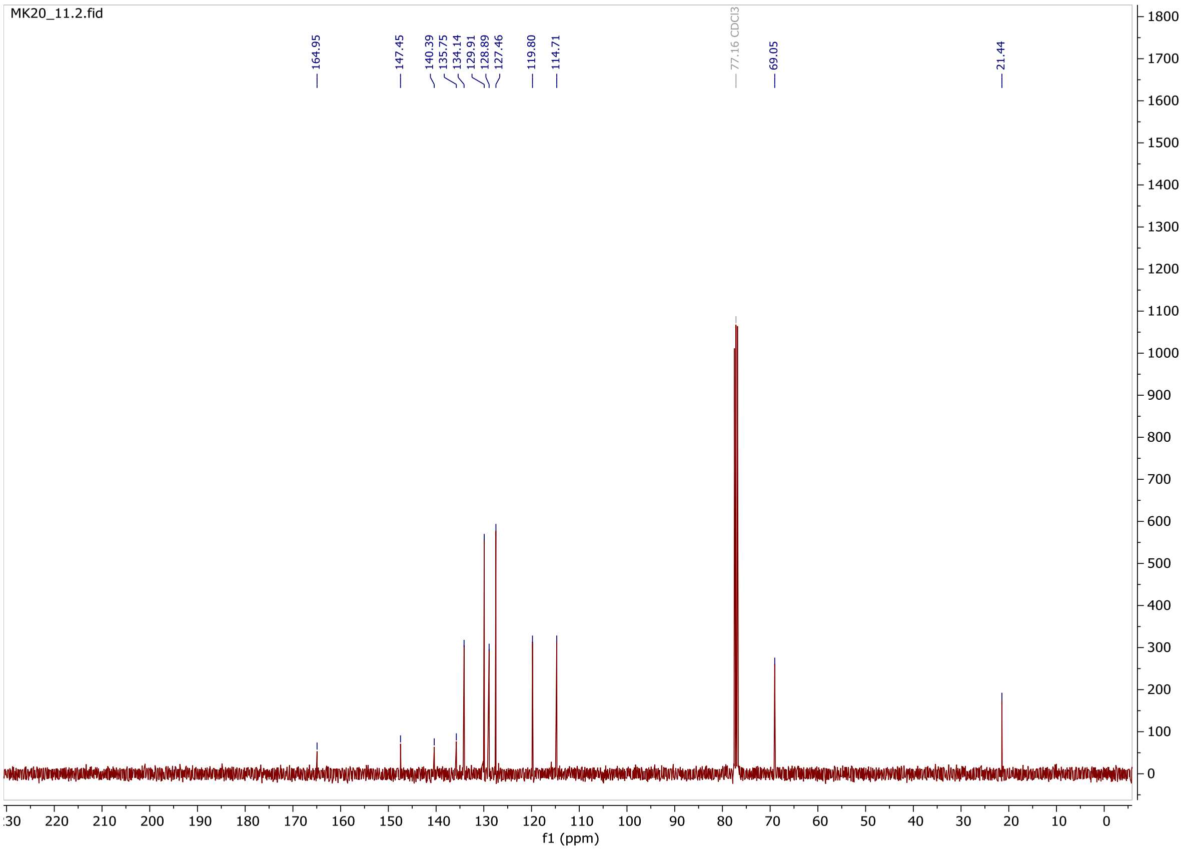

$^{13}$C{$^{1}$H} NMR (101 MHz, $CDCl_3$) of **3f**.

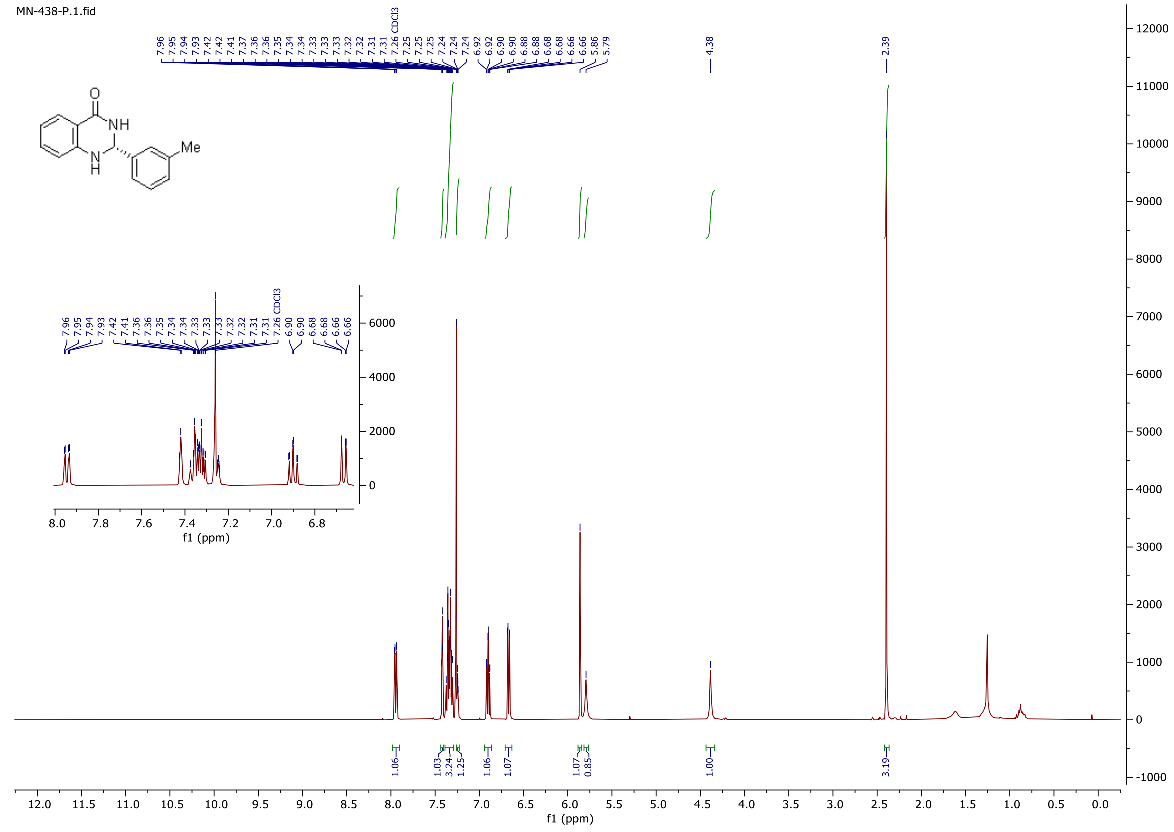

$^1$H NMR (400 MHz, $CDCl_3$) of **3g**.

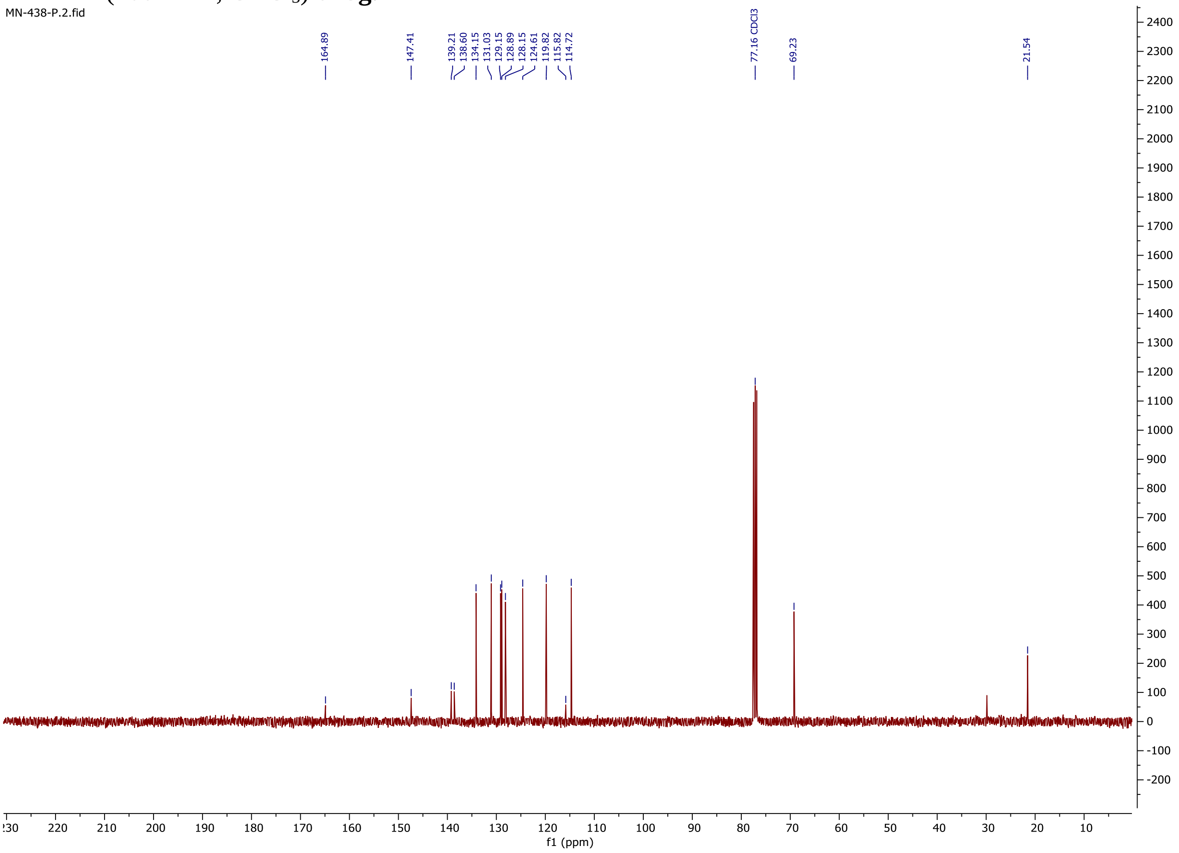

$^{13}$C{$^1$H} NMR (101 MHz, $CDCl_3$) of **3g**.

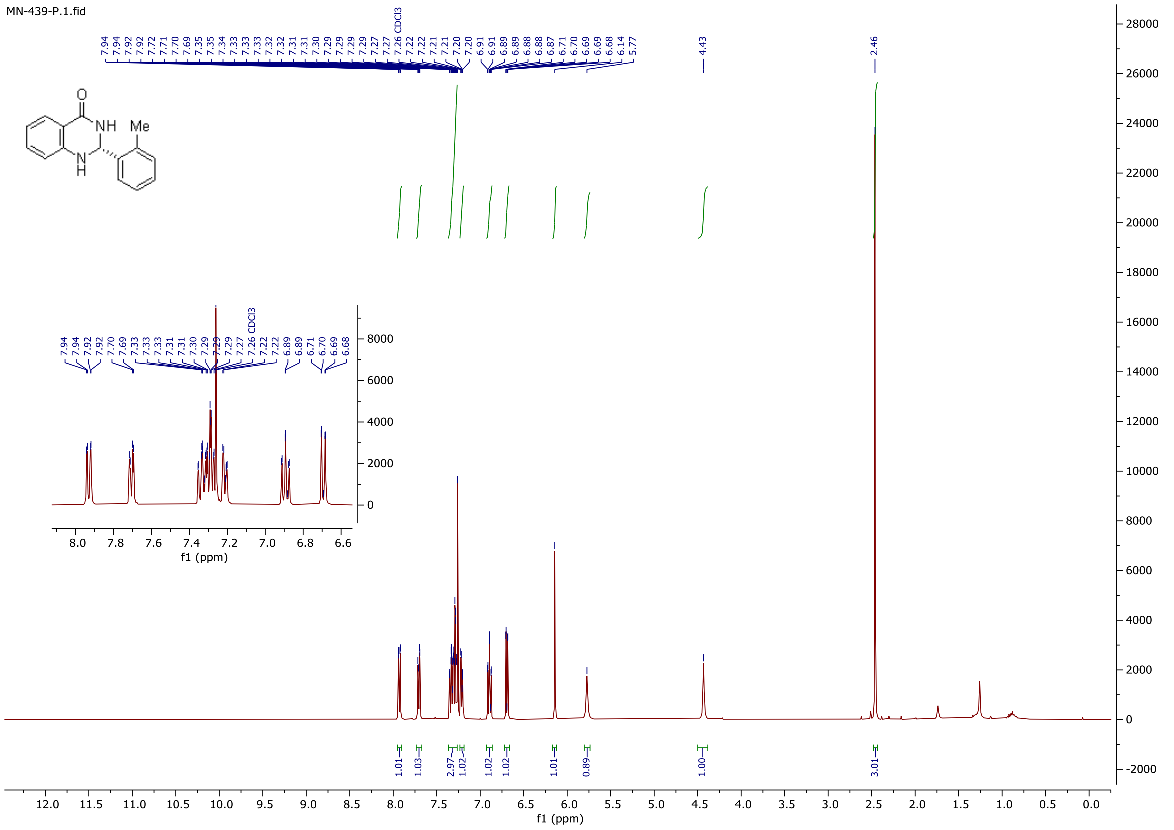

$^{1}H$ NMR (400 MHz, $CDCl_3$) of **3h**.

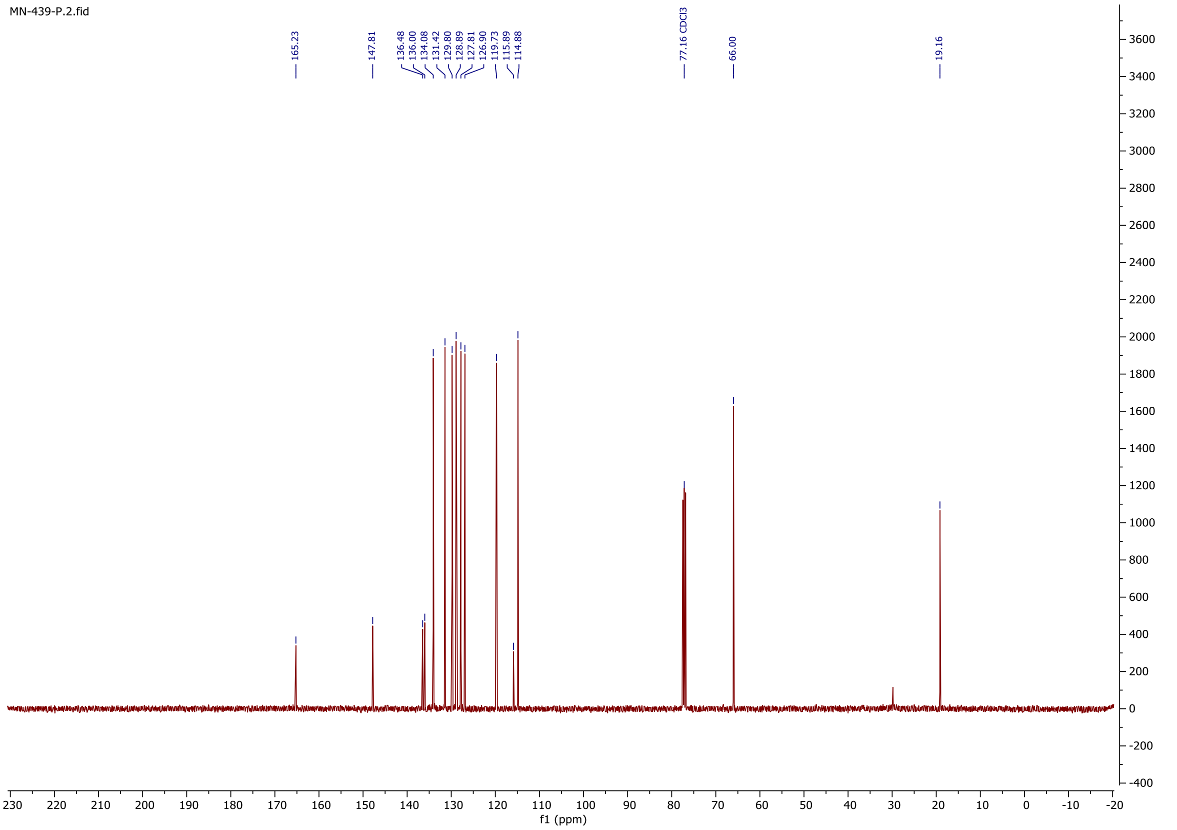

$^{13}C\{^{1}H\}$ NMR (101 MHz, $CDCl_3$) of **3h**.

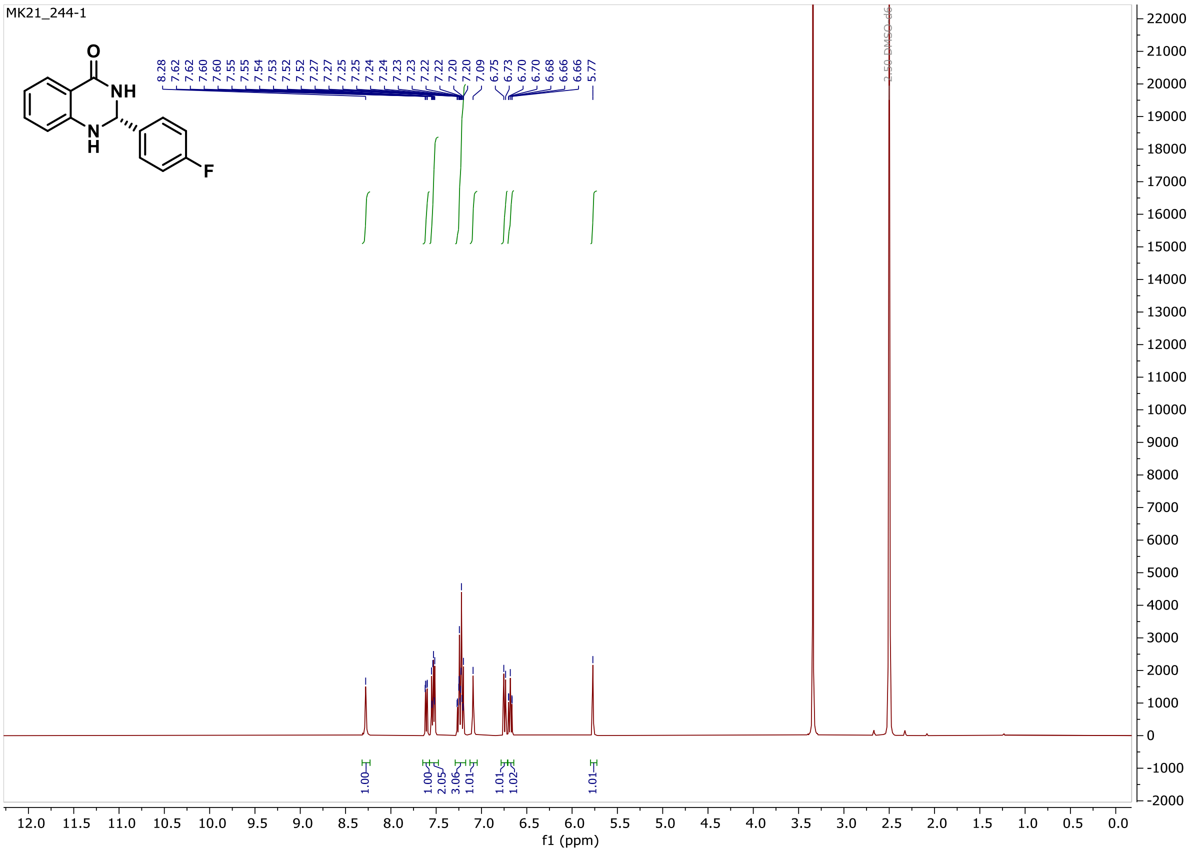

$^{1}H$ NMR (400 MHz, DMSO-$d_6$) of **3i**.

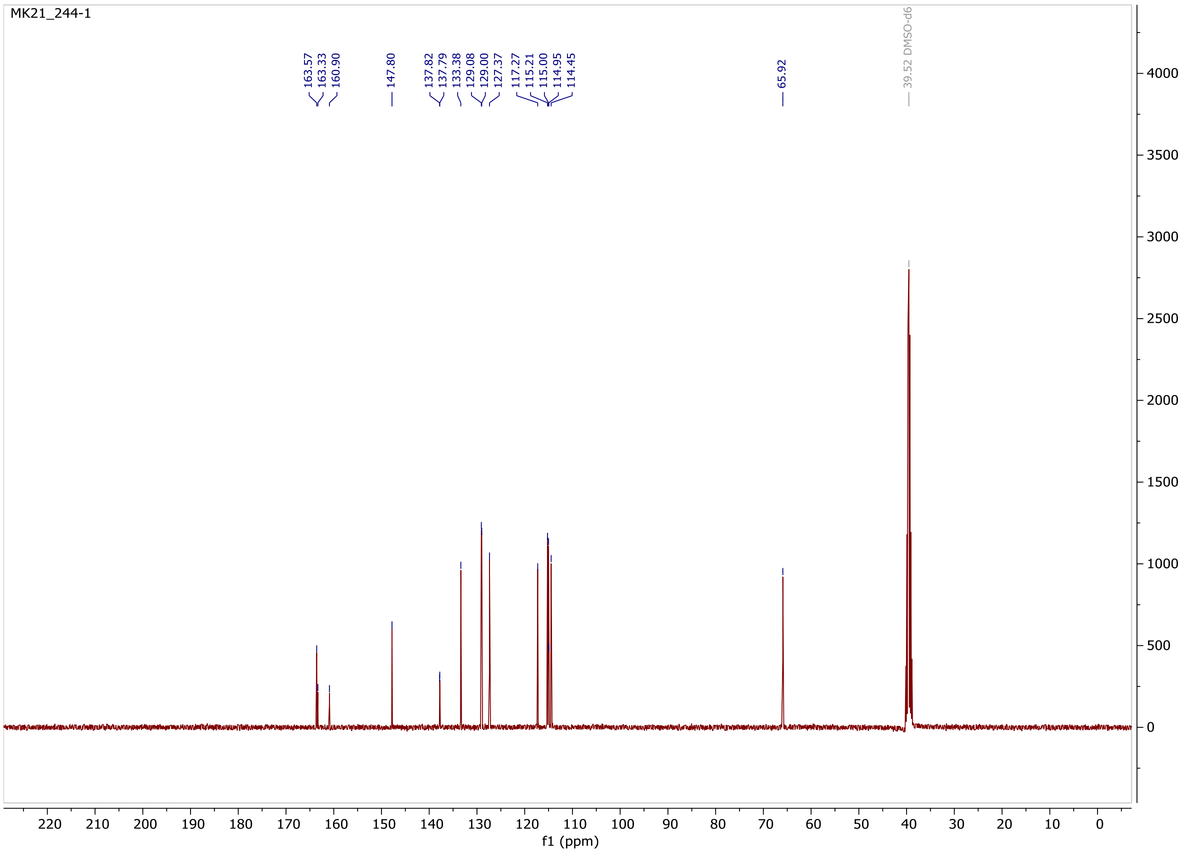

$^{13}C\{^{1}H\}$ NMR (101 MHz, DMSO-$d_6$) of **3i**.

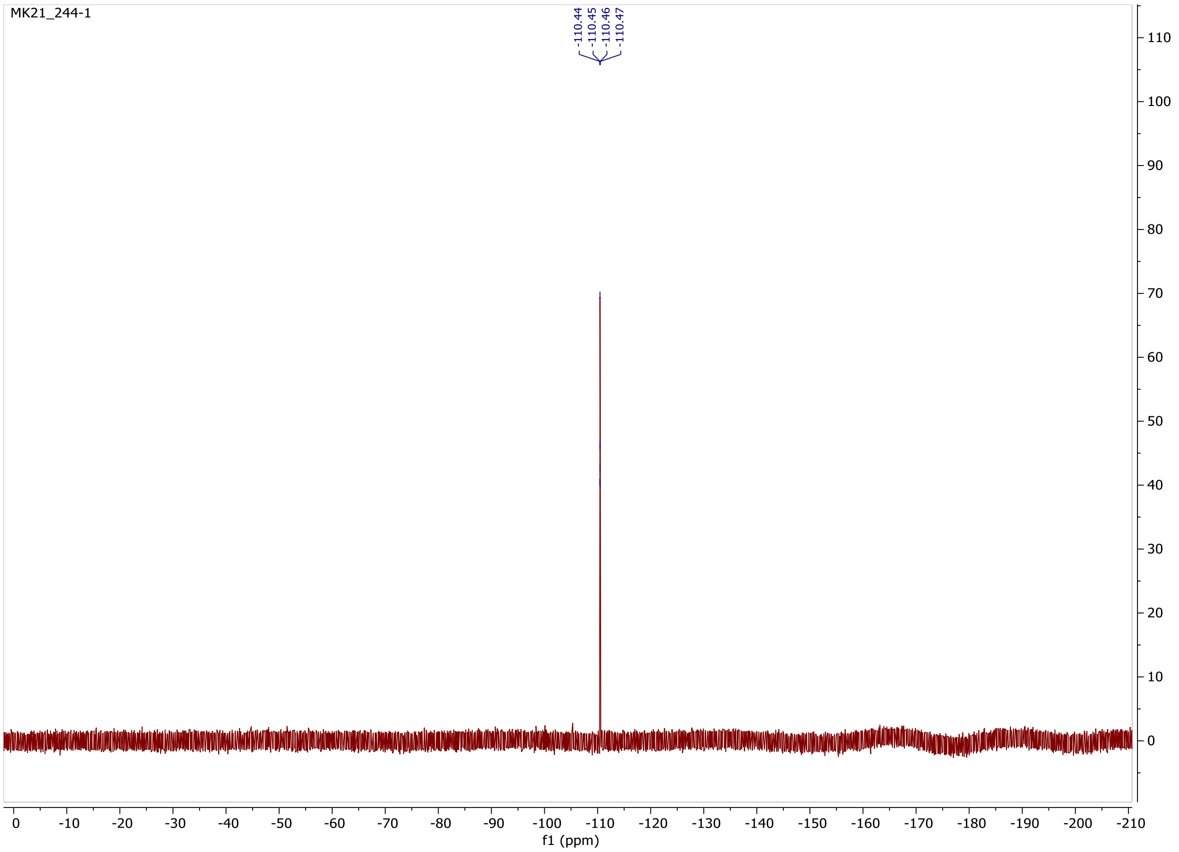


$^{19}$F NMR (376 MHz, DMSO-$d_6$) of **3i**

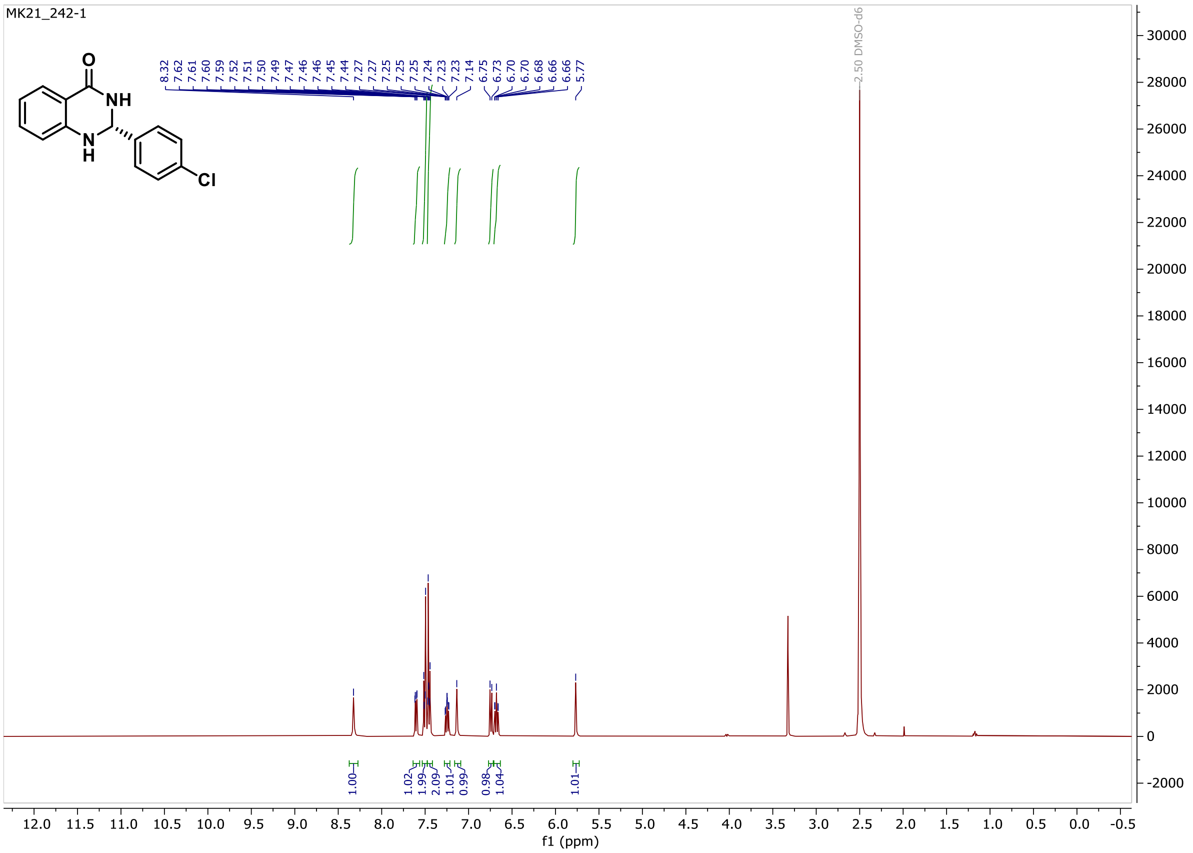


$^{1}$H NMR (400 MHz, DMSO-d$_6$) of **3j**.

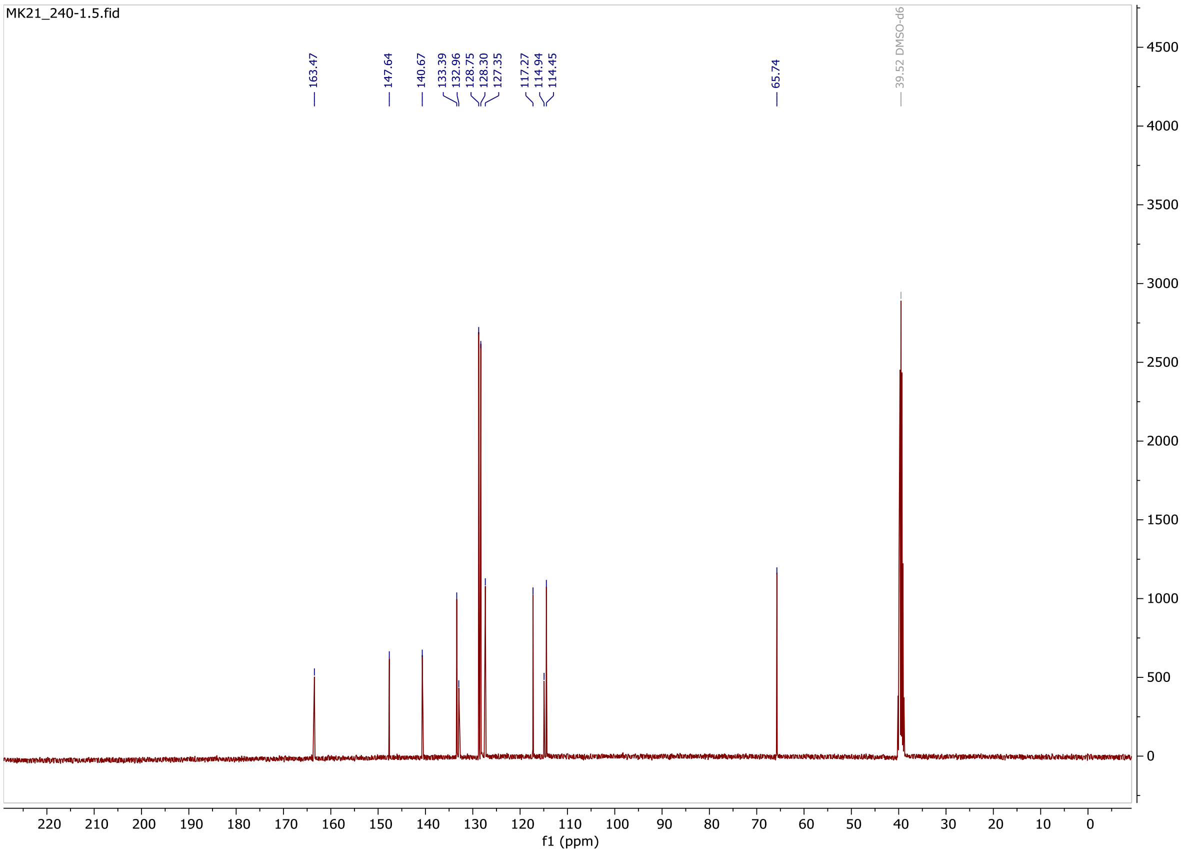


$^{13}$C{$^{1}$H} NMR (101 MHz, DMSO-d$_6$) of **3j.**

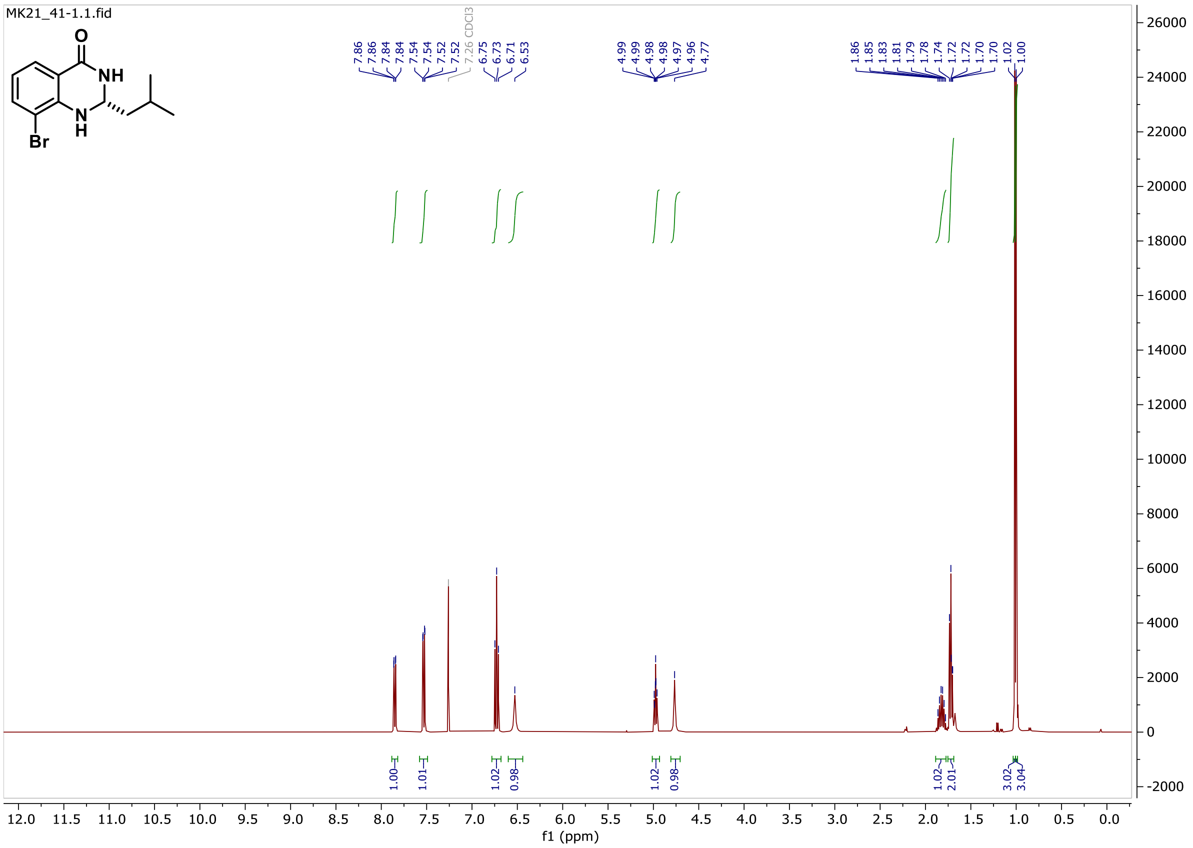

$^{1}$H NMR (400 MHz, $CDCl_3$) of **3k**.

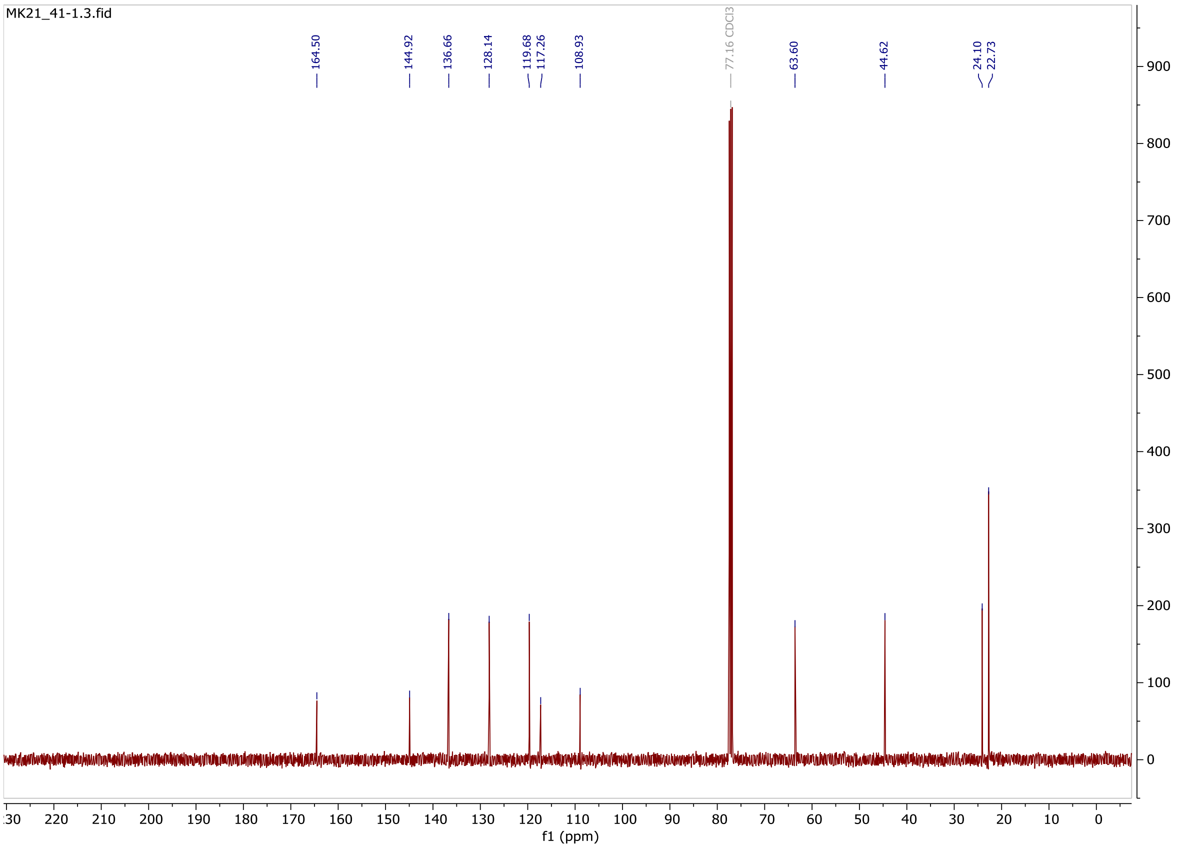

$^{13}$C{$^{1}$H} NMR (101 MHz, $CDCl_3$) of **3k**.

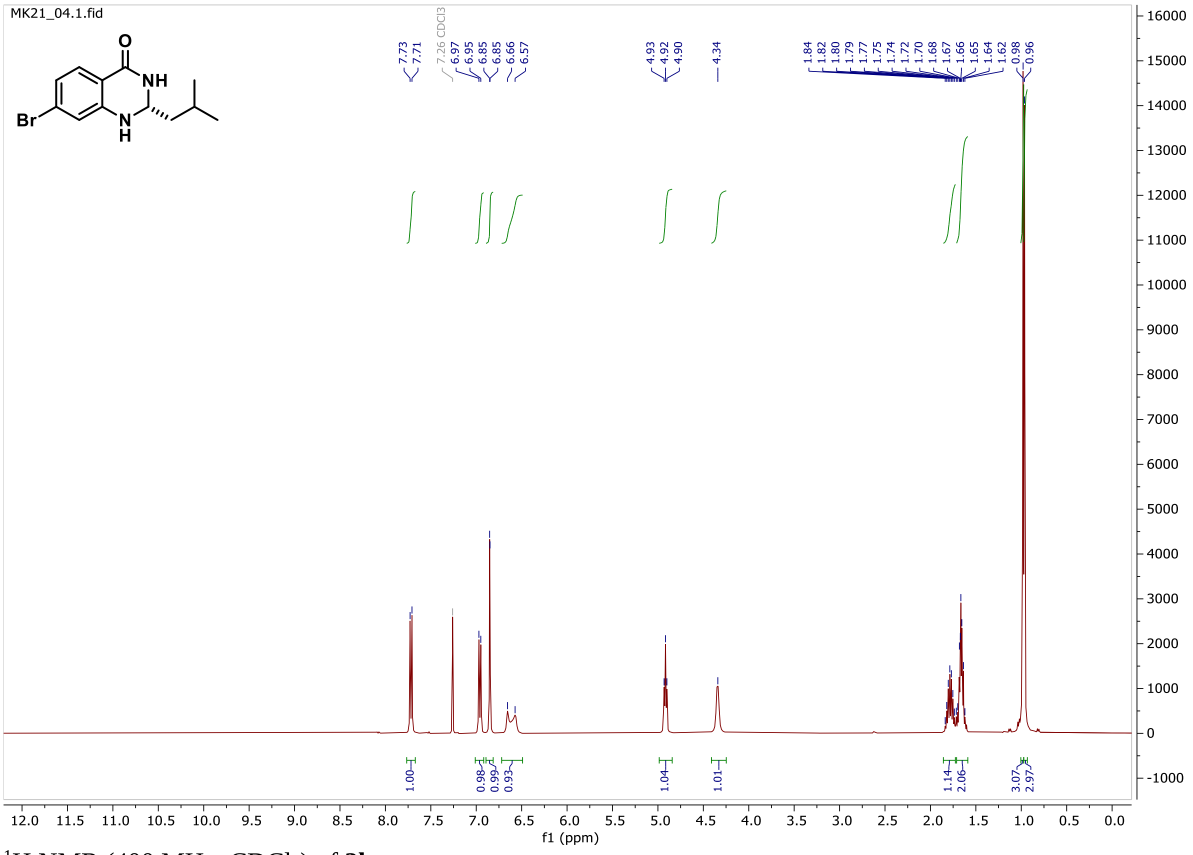

$^{1}H$ NMR (400 MHz, $CDCl_3$) of **3l**.

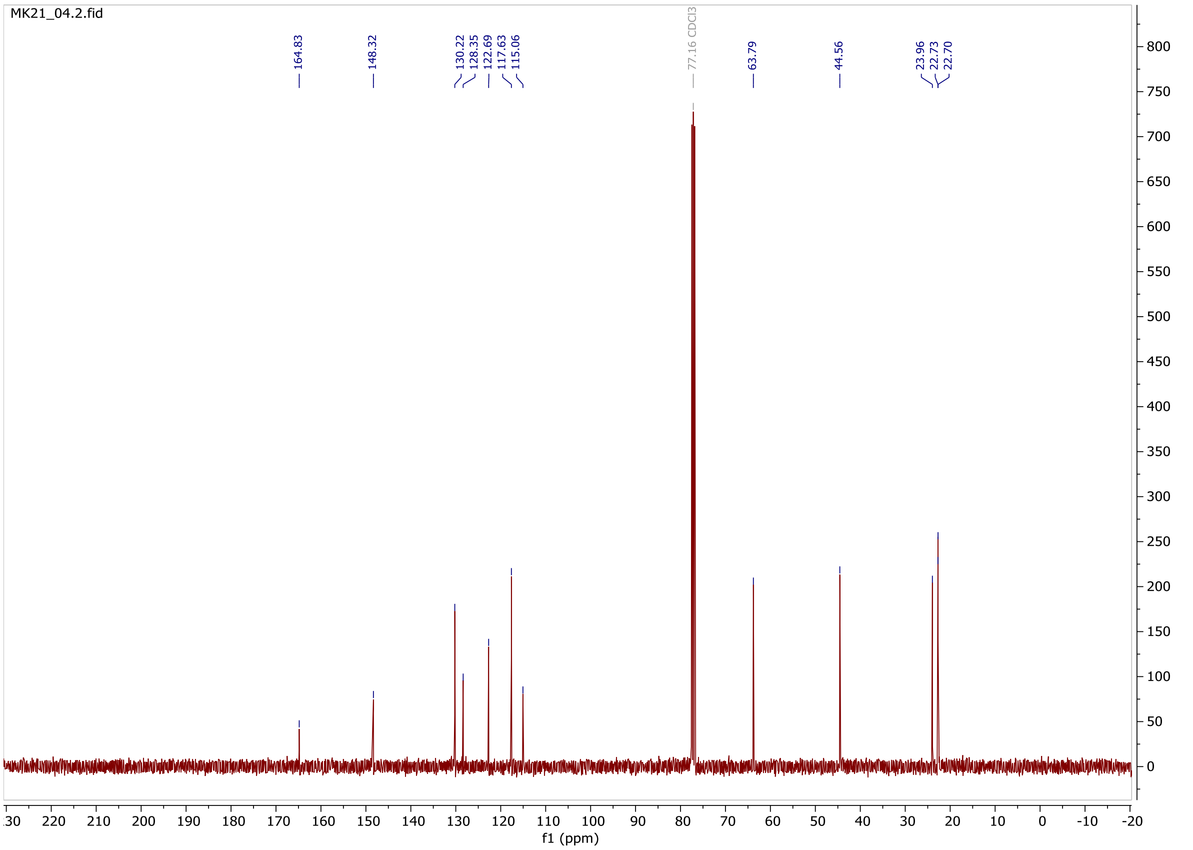

$^{13}C\{^{1}H\}$ NMR (101 MHz, $CDCl_3$) of **3l**.

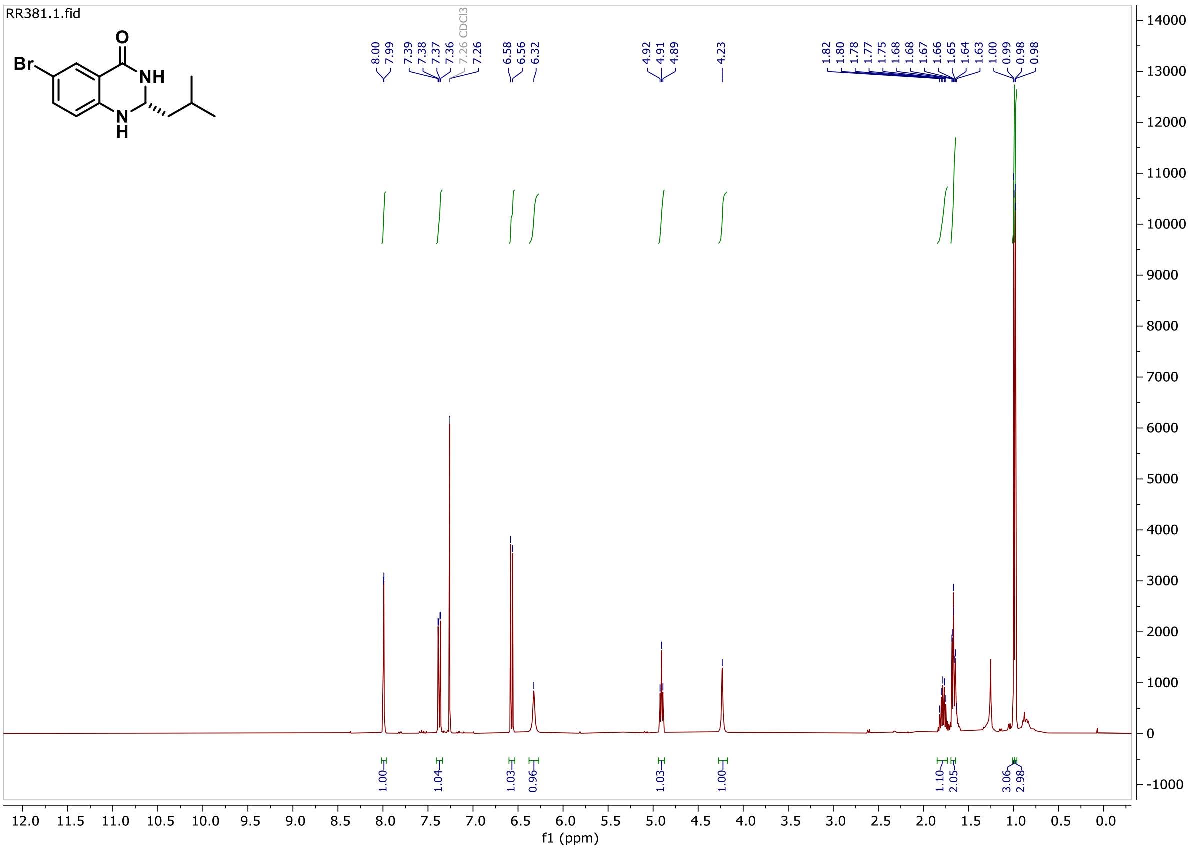

$^{1}$H NMR (400 MHz, $CDCl_3$) of **3m**.

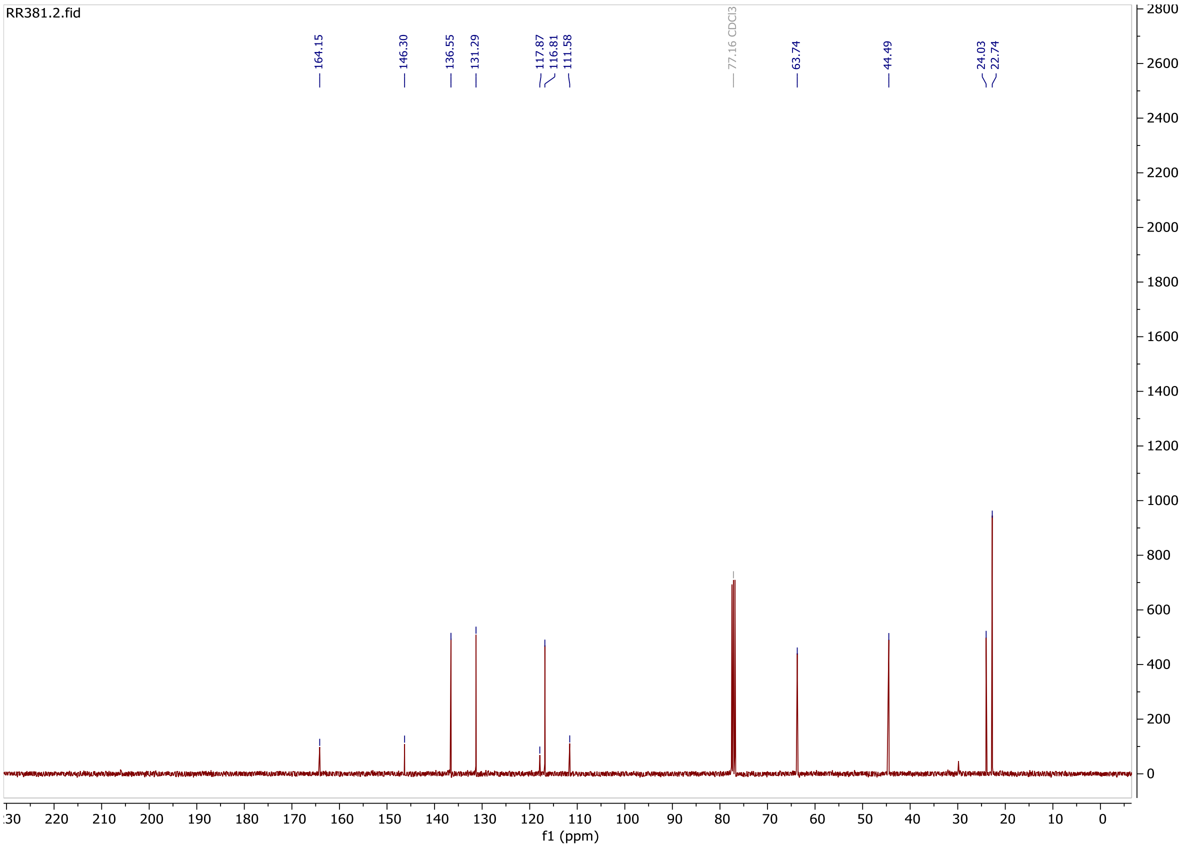

$^{13}$C{$^{1}$H} NMR (101 MHz, $CDCl_3$) of **3m**.

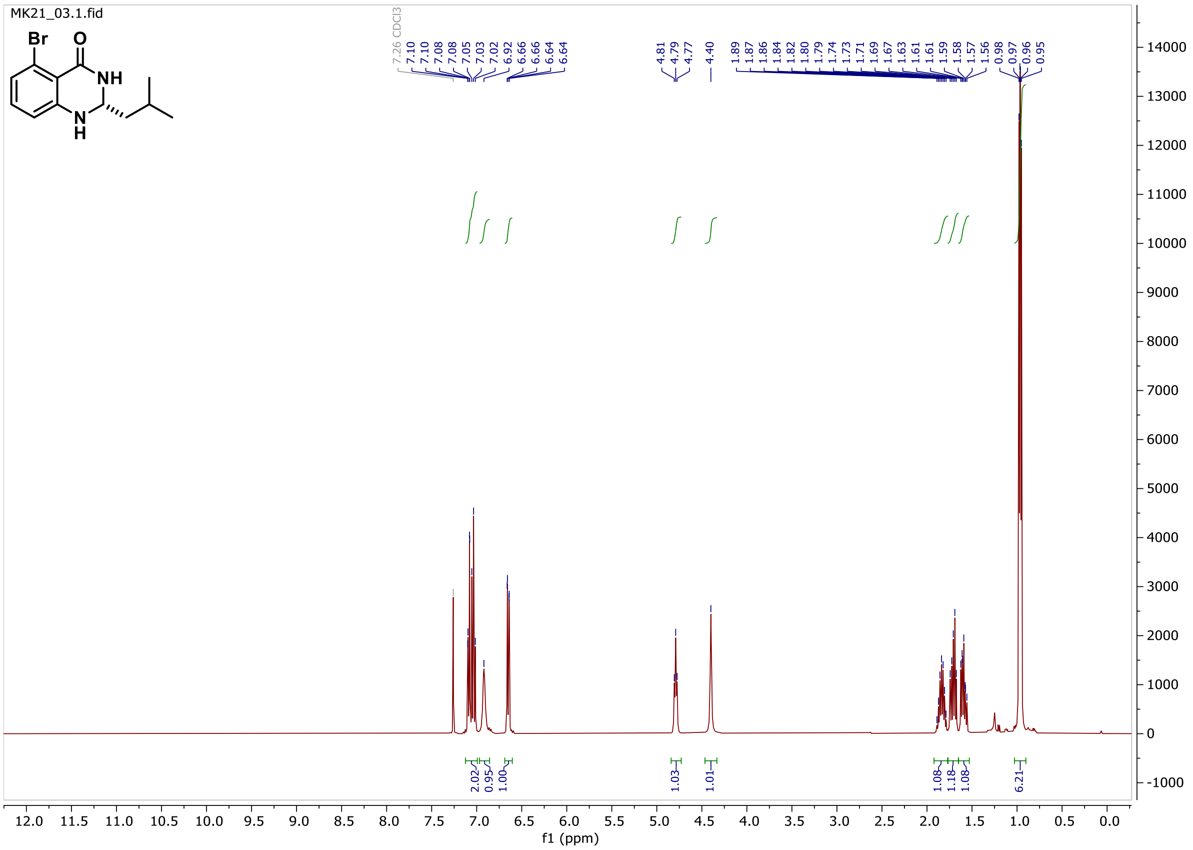

$^1$H NMR (400 MHz, $CDCl_3$) of **3n**.

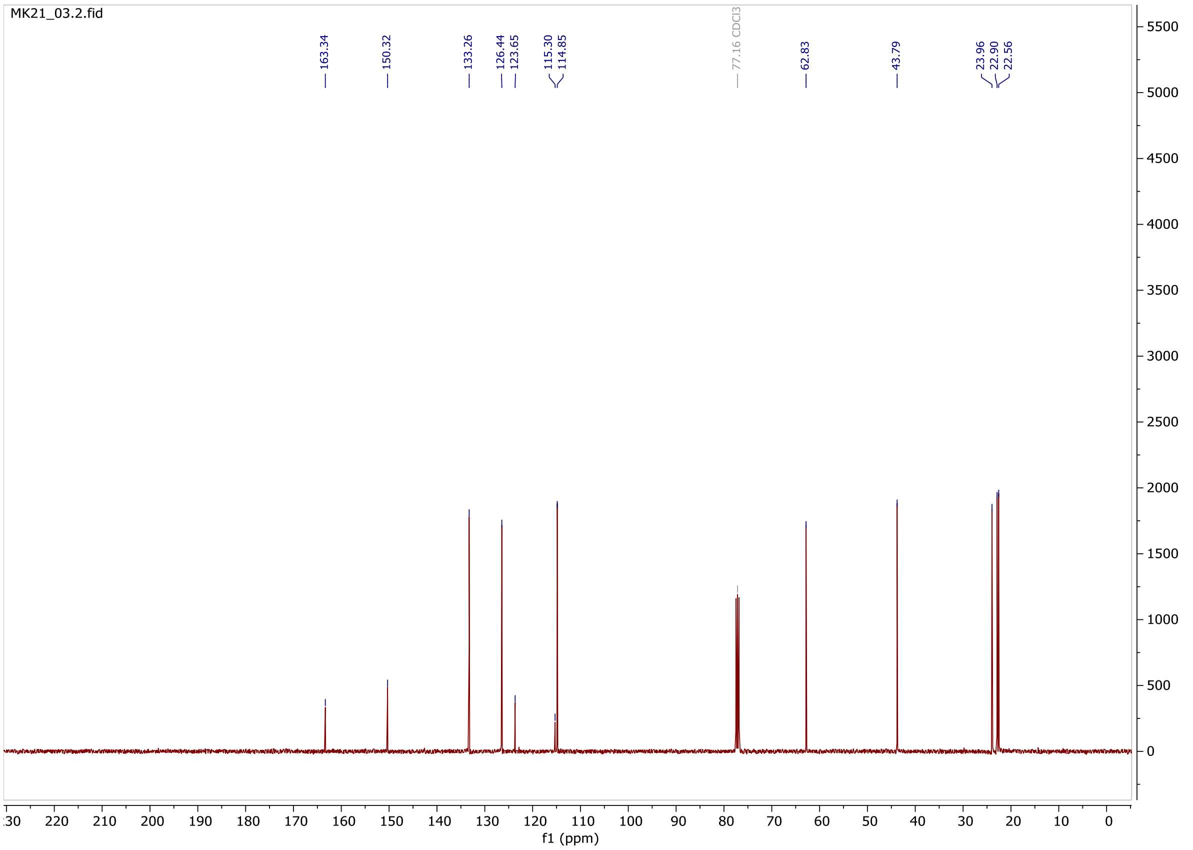

$^{13}C\{^1H\}$ NMR (101 MHz, $CDCl_3$) of **3n**.

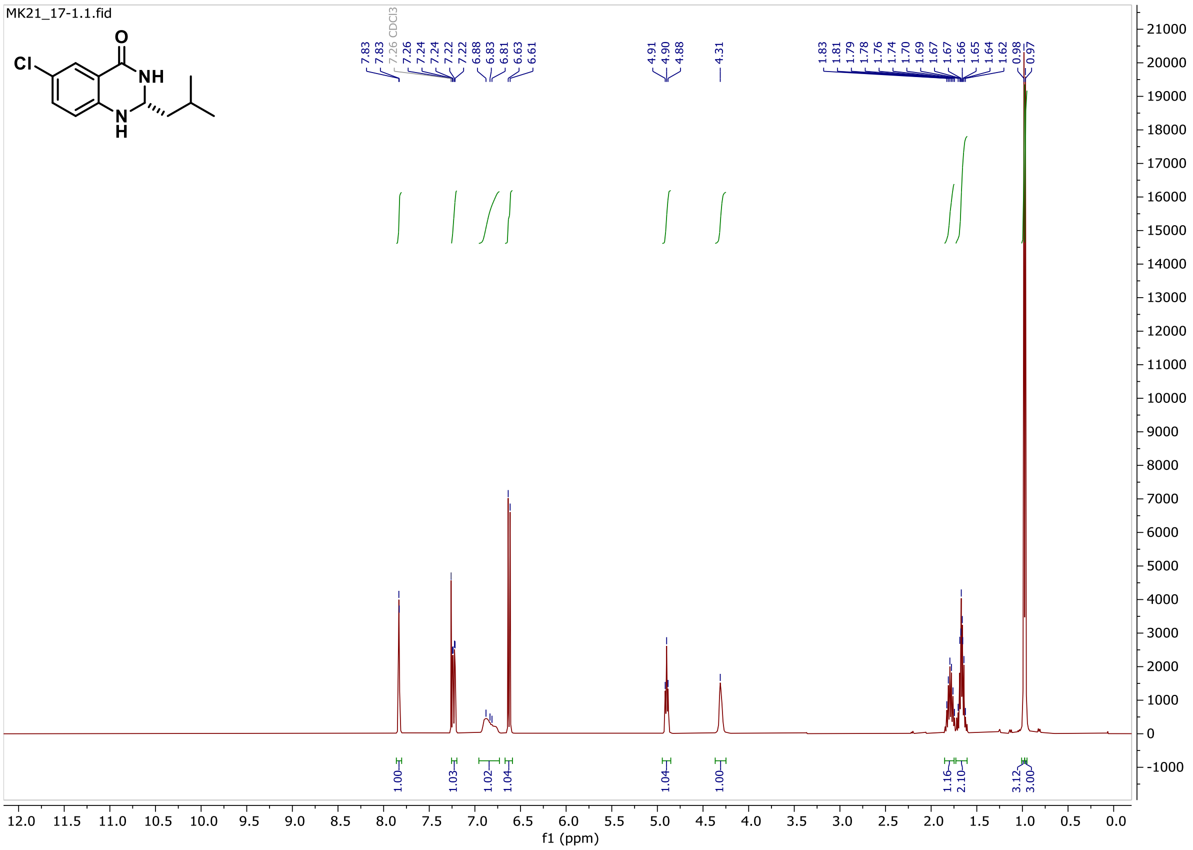


$^{1}$H NMR (400 MHz, $CDCl_3$) of **3o**.

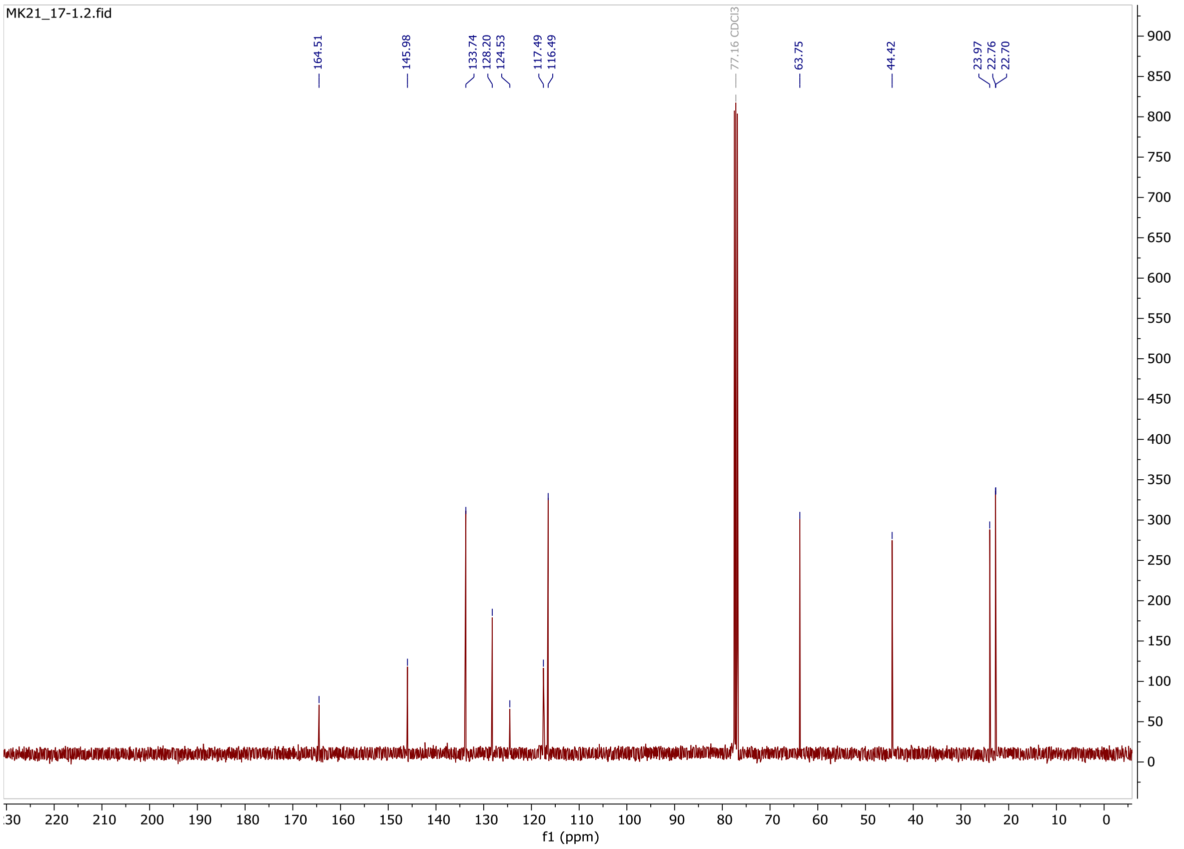


$^{13}$C{$^{1}$H} NMR (101 MHz, $CDCl_3$) of **3o**.

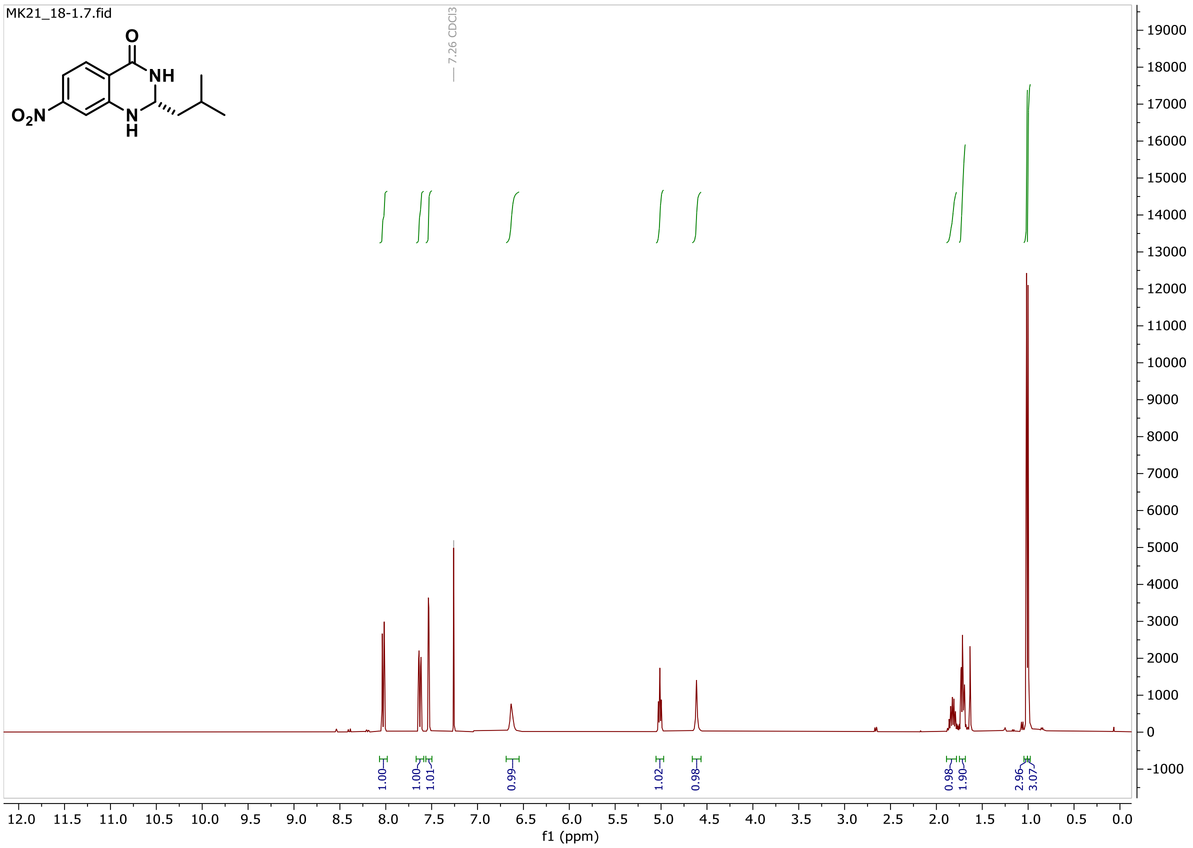


$^{1}$H NMR (400 MHz, $CDCl_3$) of **3p**.

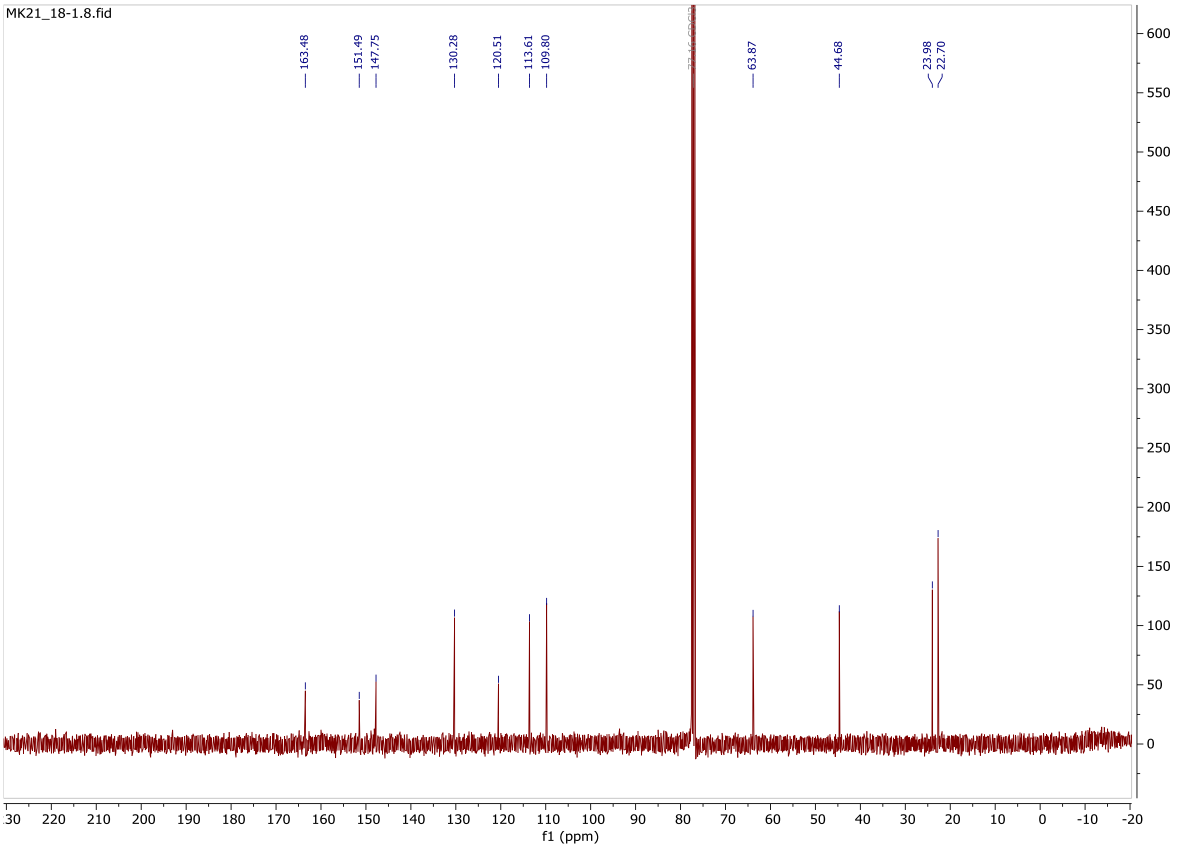


$^{13}$C{$^{1}$H} NMR (101 MHz, $CDCl_3$) of **3p**.

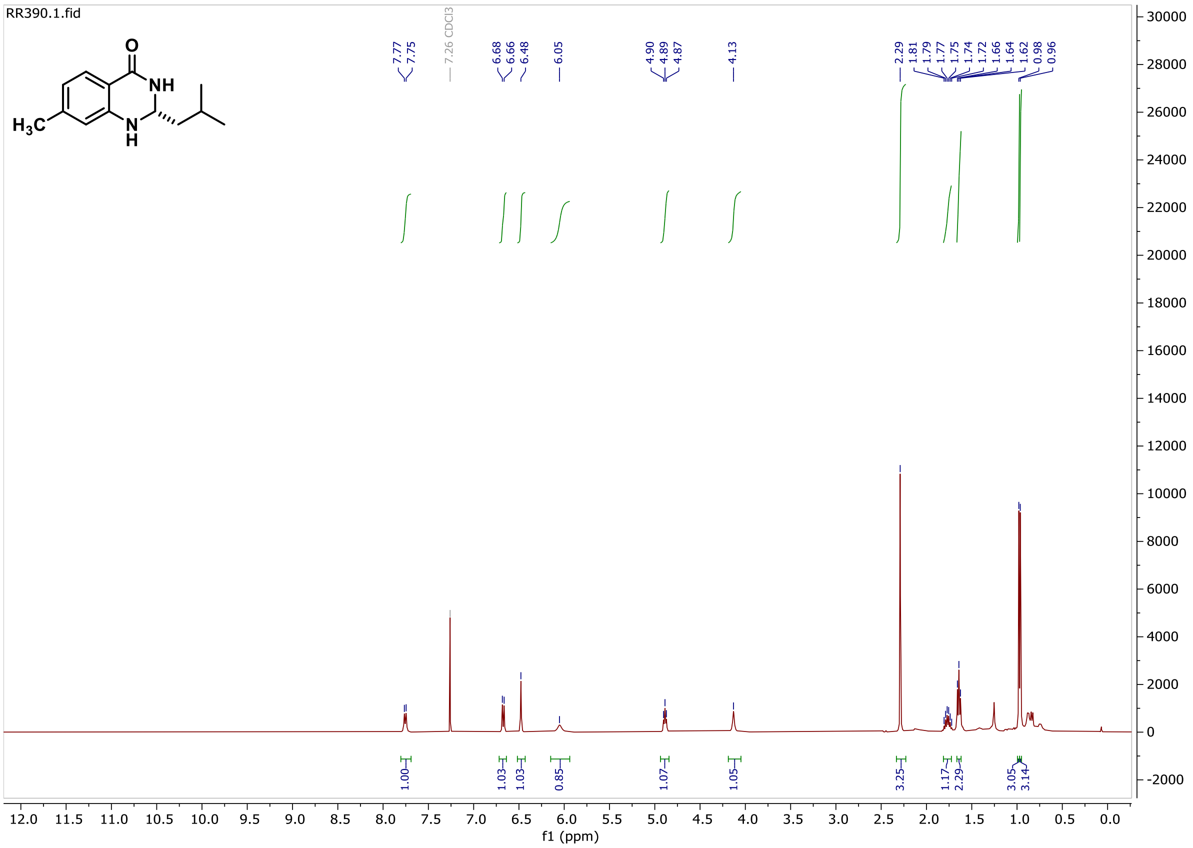

$^{1}H$ NMR (400 MHz, $CDCl_3$) of **3q**.

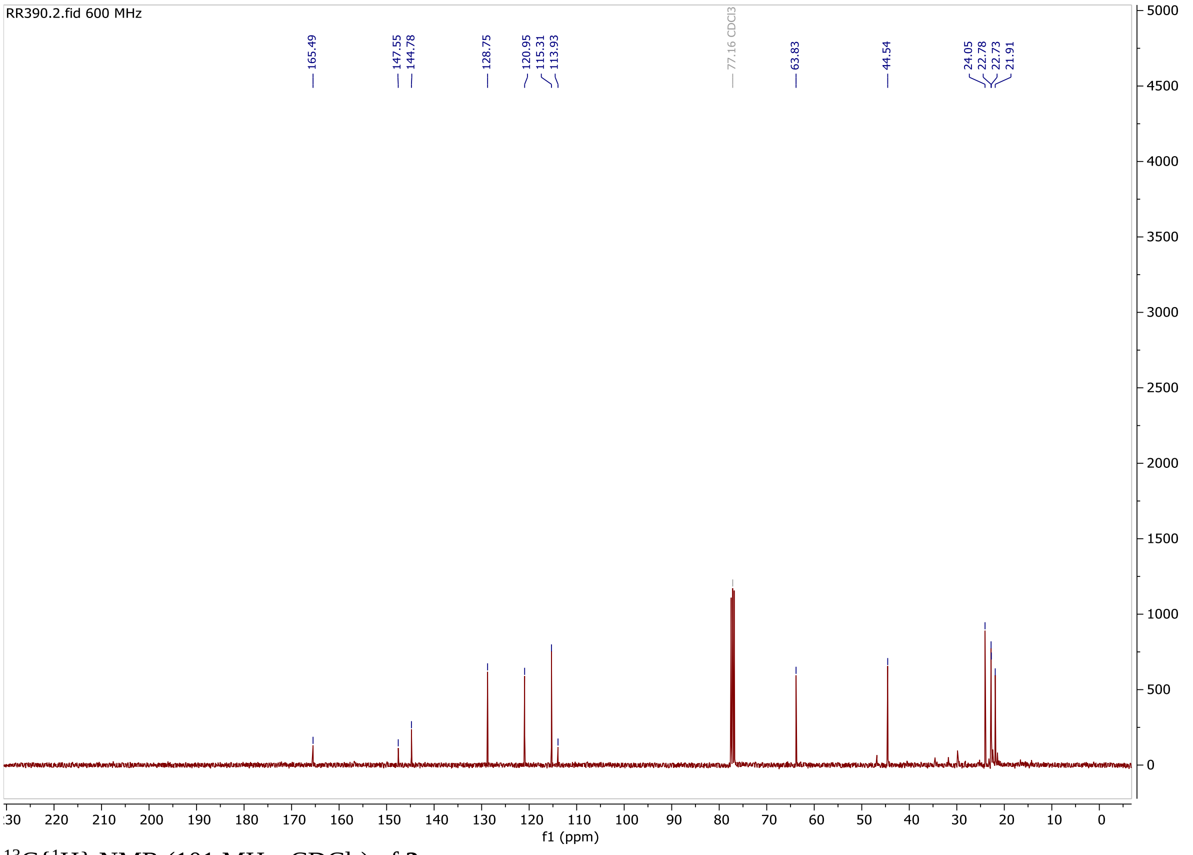

$^{13}C\{^{1}H\}$ NMR (101 MHz, $CDCl_3$) of **3q**.

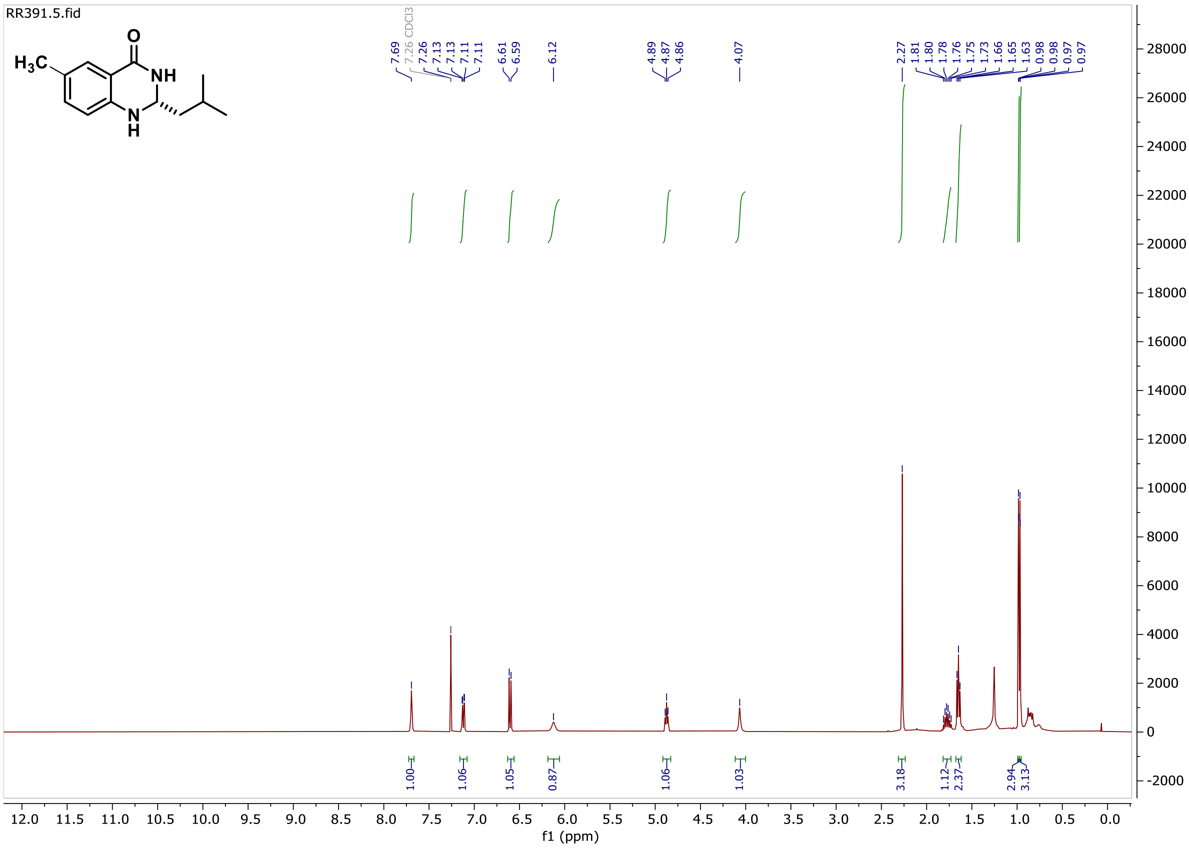

$^{1}H$ NMR (400 MHz, $CDCl_3$) of **3r**.

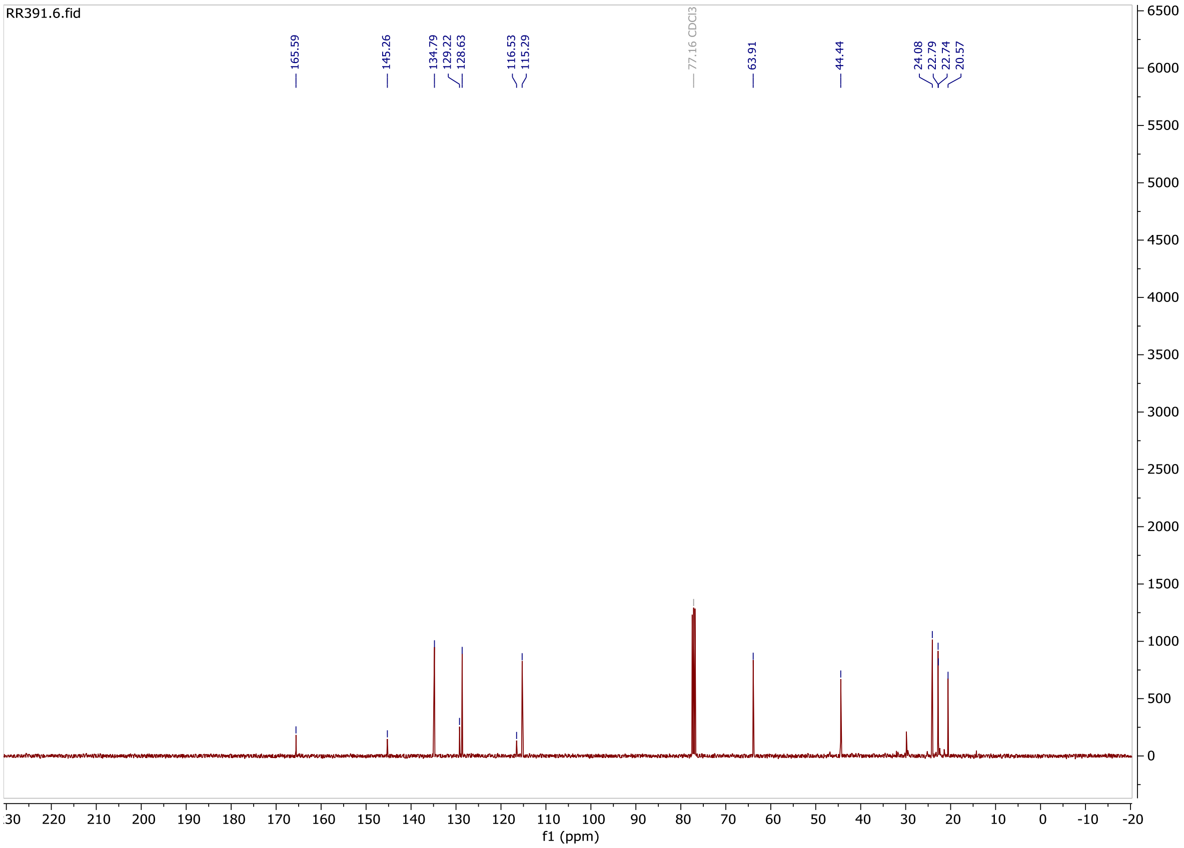

$^{13}C\{^{1}H\}$ NMR (101 MHz, $CDCl_3$) of **3r**.

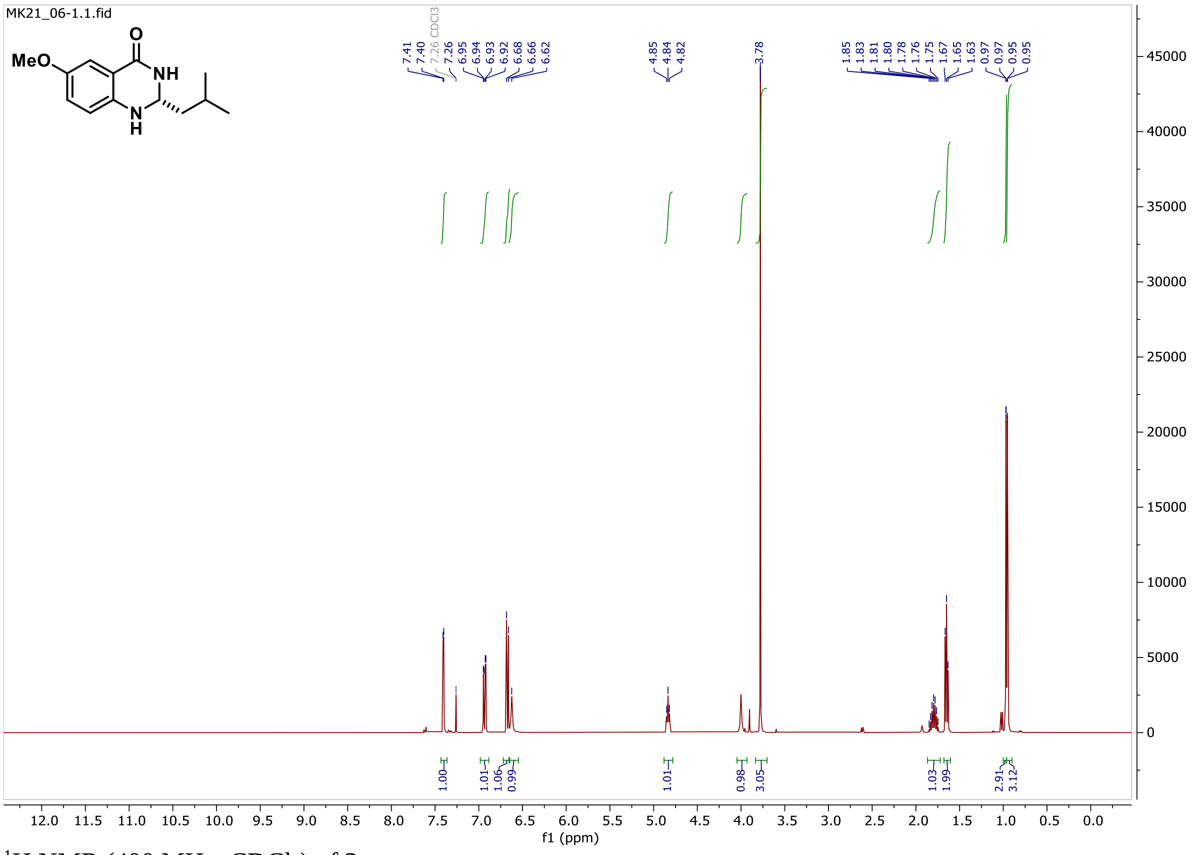


$^{1}$H NMR (400 MHz, $CDCl_3$) of **3s**.

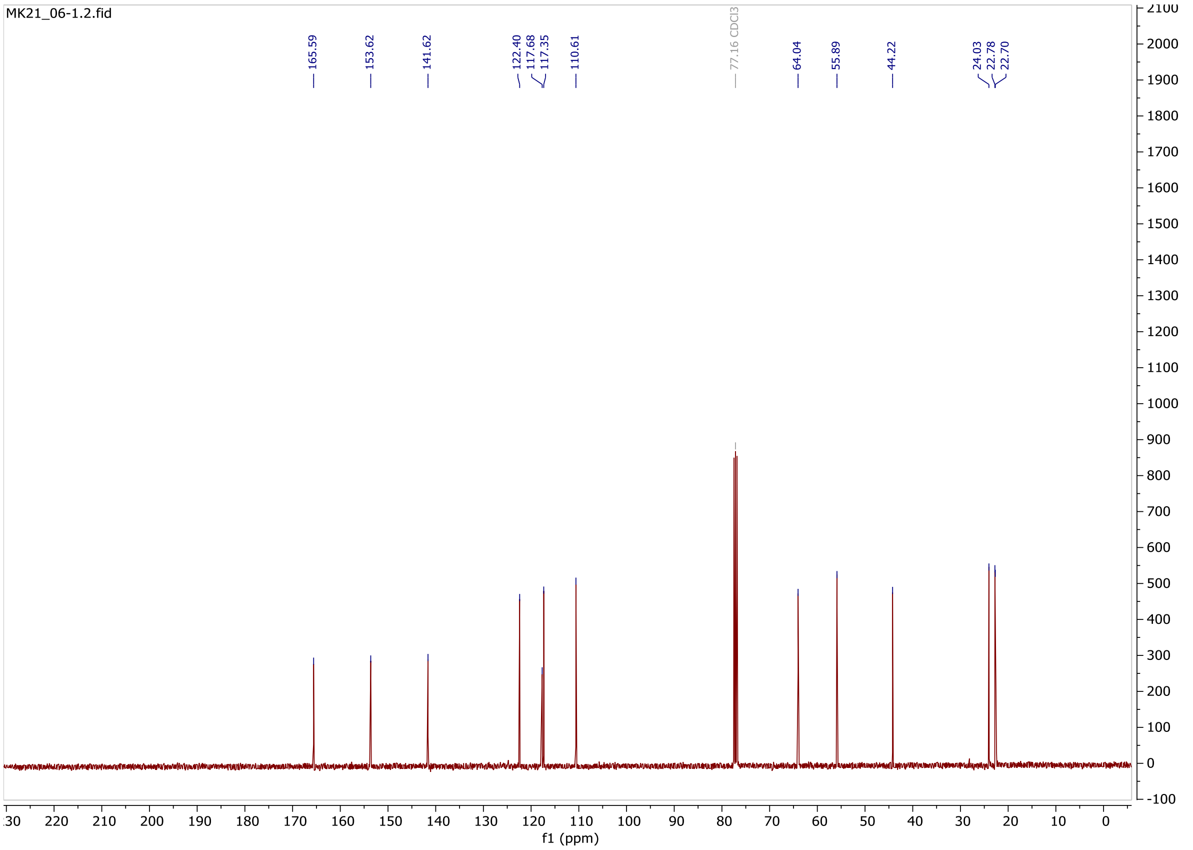


$^{13}$C{$^{1}$H} NMR (101 MHz, $CDCl_3$) of **3s**.

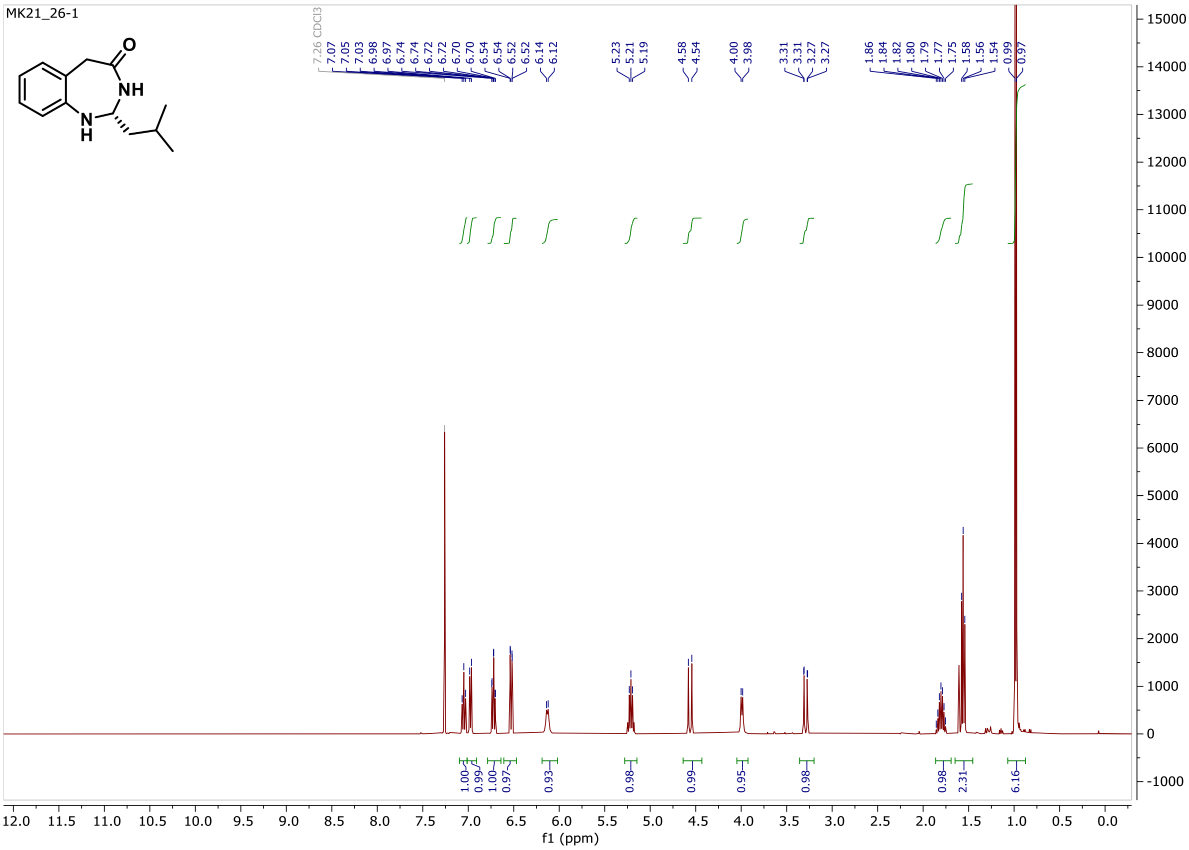

$^{1}$H NMR (400 MHz, $CDCl_3$) of **3t**.

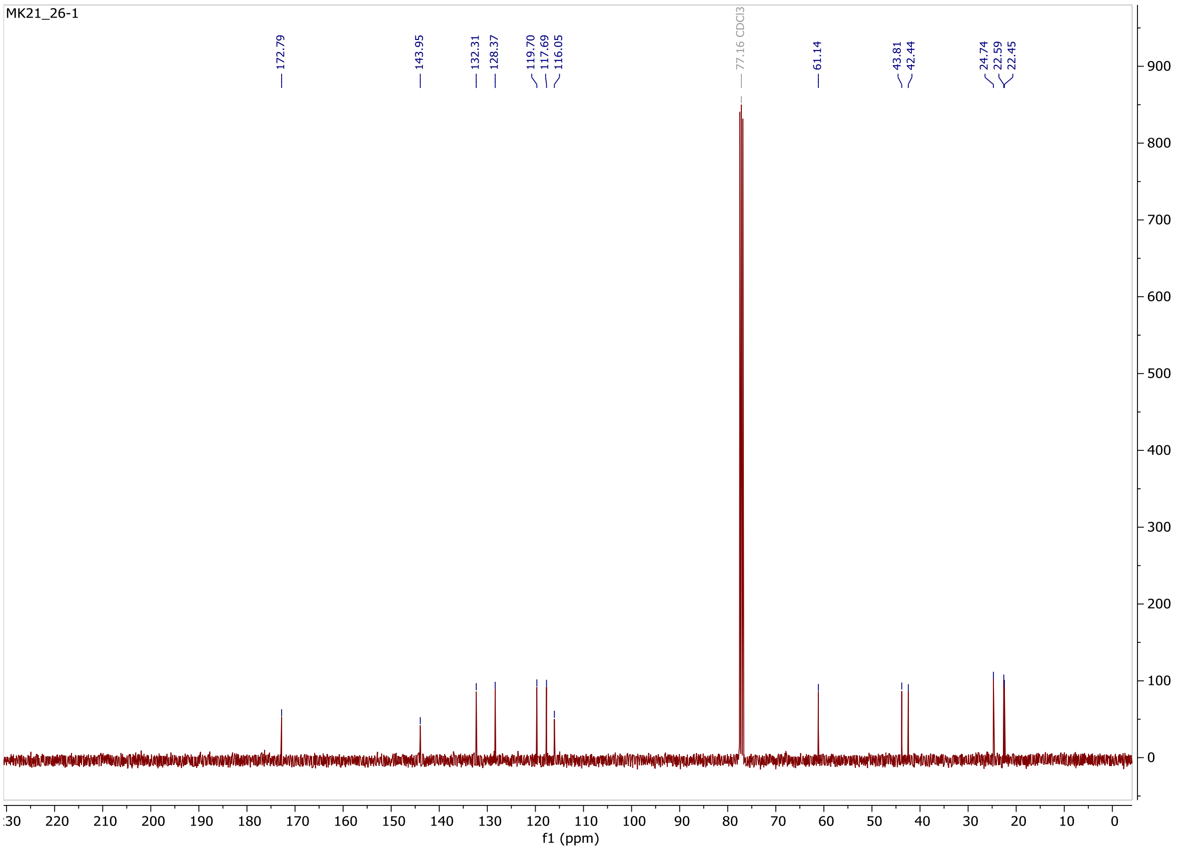

$^{13}$C{$^{1}$H} NMR (101 MHz, $CDCl_3$) of **3t**.

# HPLC chromatograms

**Conditions:** OD-H column, mobile phase: *n*-heptane/iPrOH 80:20, $\lambda$= 191 nm, $V$ = 1.0 mL/min, $T$ = 25 °C, $t_R$ = 8.1 min (minor), $t_R$ = 10.1 min (major), *ee* 80% (93% after recrystallization).

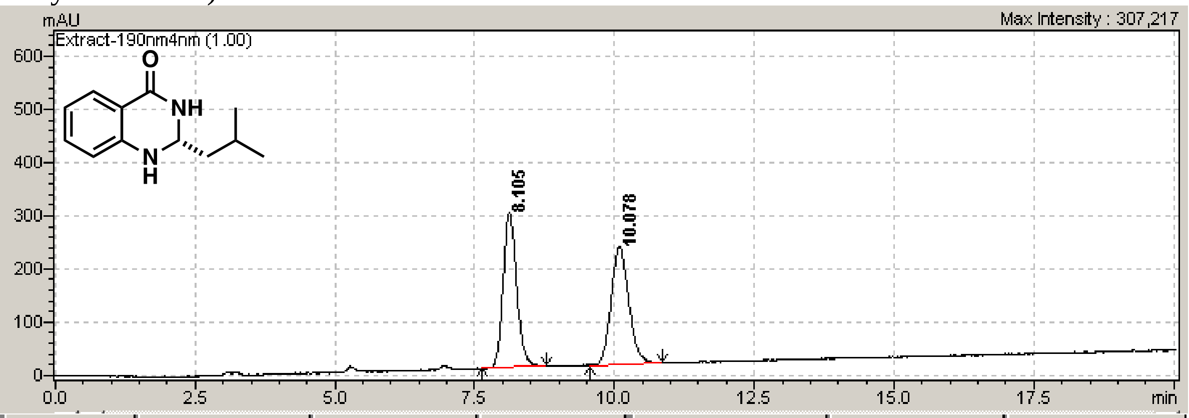


| Ret. Time | Conc. | Area | Height | Peak Start | Peak End | Area% |
|---|---|---|---|---|---|---|
| 8.105 | 50.24399 | 4883478 | 292154 | 7.637 | 8.768 | 50.2440 |
| 10.078 | 49.75601 | 4836048 | 220875 | 9.568 | 10.859 | 49.7560 |

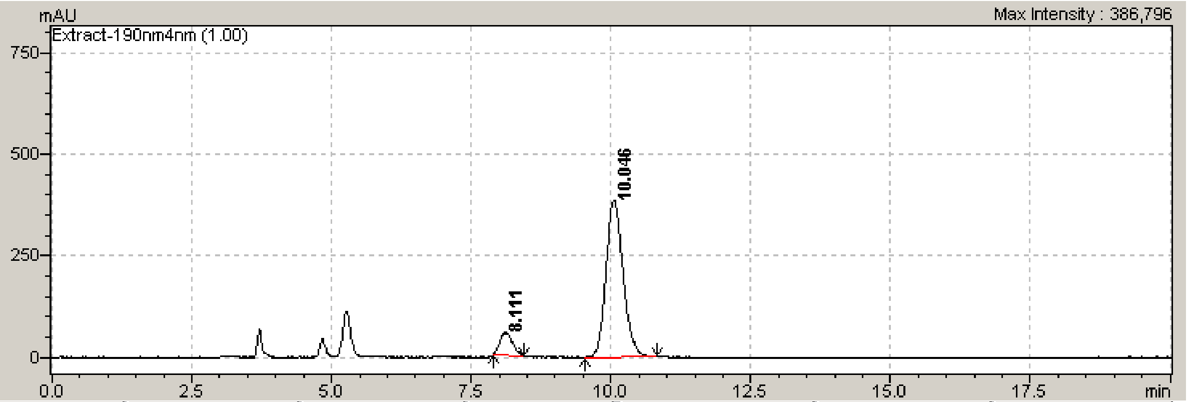


| Ret. Time | Conc. | Area | Height | Peak Start | Peak End | Area% |
|---|---|---|---|---|---|---|
| 8.111 | 9.85331 | 899375 | 56975 | 7.883 | 8.448 | 9.8533 |
| 10.046 | 90.14669 | 8228274 | 386294 | 9.547 | 10.837 | 90.1467 |

After recrystallization of **3a**.

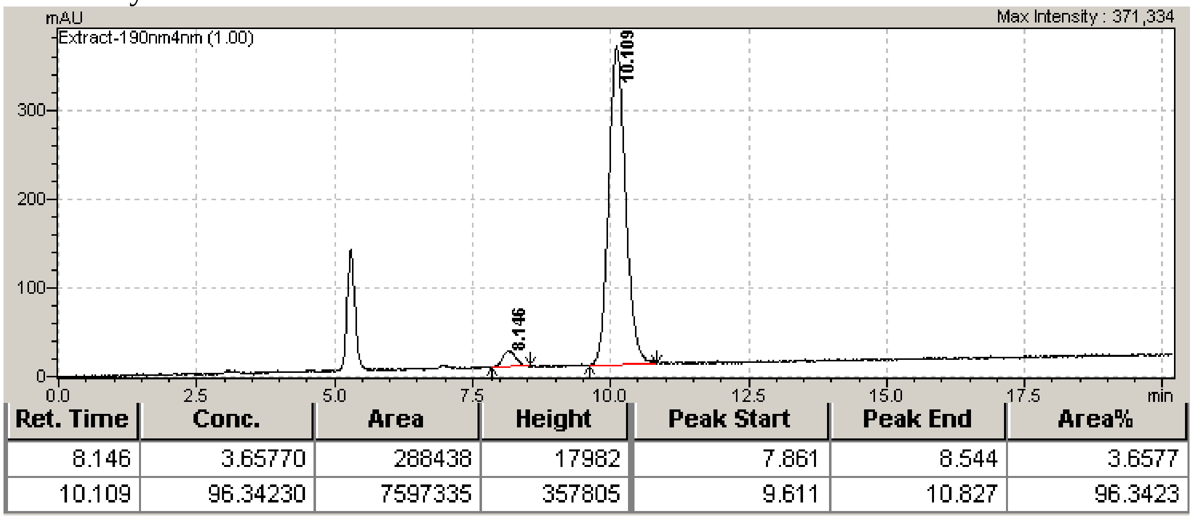


| Ret. Time | Conc. | Area | Height | Peak Start | Peak End | Area% |
|---|---|---|---|---|---|---|
| 8.146 | 3.65770 | 288438 | 17982 | 7.861 | 8.544 | 3.6577 |
| 10.109 | 96.34230 | 7597335 | 357805 | 9.611 | 10.827 | 96.3423 |

**Conditions:** IA column, mobile phase: *n*-heptane/iPrOH 80:20, $\lambda$ = 190 nm, $V$ = 1.0 mL/min, $T$ = 25 °C, $t_R$ = 8.0 min (minor), $t_R$ = 9.7 min (major), *ee* 74%.

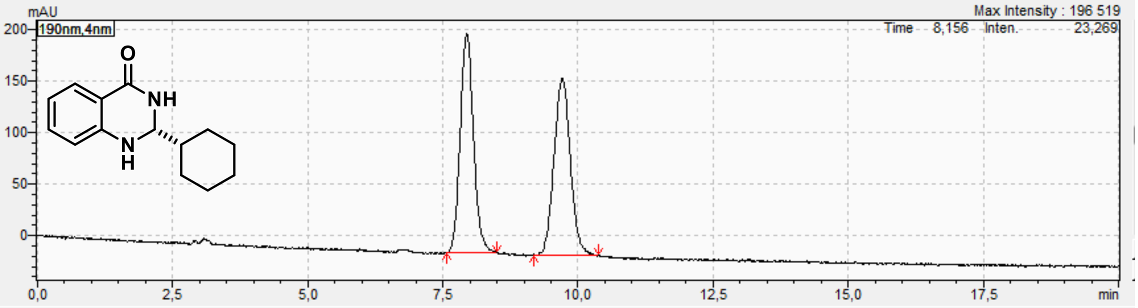


sults View - Peak Table

Compound | Group | Calibration Curve

| Ret. Time | Conc. | Area | Height | Similarity Index | Mark | Peak Start | Peak End | Area% |
|---|---|---|---|---|---|---|---|---|
| 7,945 | 50,235 | 3641882 | 213306 | 0,000000 | M | 7,563 | 8,501 | 50,235 |
| 9,713 | 49,765 | 3607798 | 171901 | 0,000000 | M | 9,184 | 10,368 | 49,765 |
| | 100,000 | 7249679 | 385206 | | | | | 100,000 |

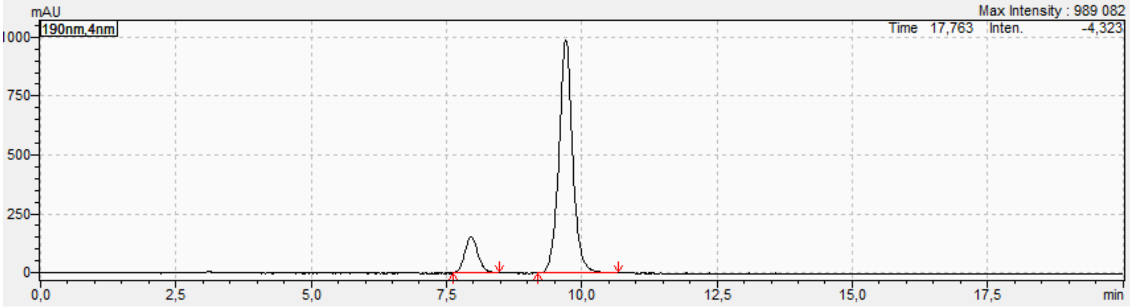


sults View - Peak Table

Compound | Group | Calibration Curve

| Ret. Time | Conc. | Area | Height | Similarity Index | Mark | Peak Start | Peak End | Area% |
|---|---|---|---|---|---|---|---|---|
| 7,960 | 12,925 | 2597782 | 152465 | 0,000000 | M | 7,616 | 8,469 | 12,925 |
| 9,705 | 87,075 | 17500445 | 990758 | 0,000000 | M | 9,184 | 10,688 | 87,075 |
| | 100,000 | 20098227 | 1143223 | | | | | 100,000 |

**Conditions:** IH column, mobile phase: *n*-heptane/iPrOH 80:20, $\lambda$ = 190 nm, $V$ = 1.0 mL/min, $T$ = 25 °C, $t_R$ = 12.1 min (minor), $t_R$ = 13.9 min (major), *ee* 10%.

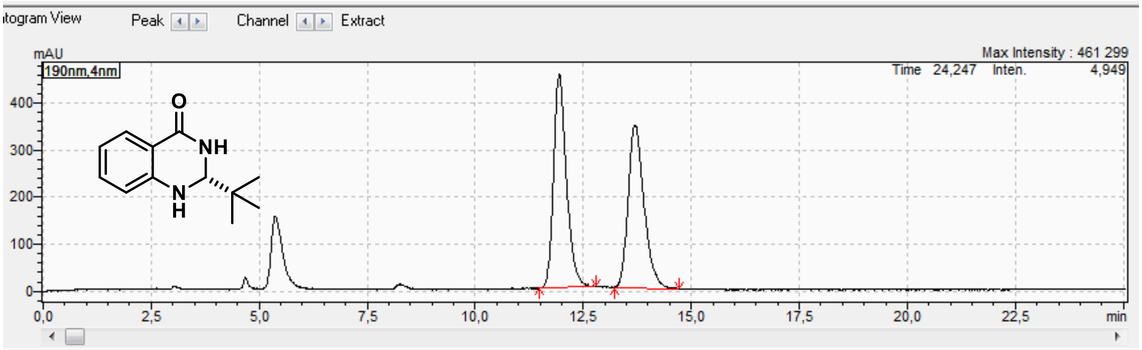


sults View - Peak Table

Compound | Group | Calibration Curve

| Ret. Time | Conc. | Area | Height | Similarity Index | Mark | Peak Start | Peak End | Area% |
|---|---|---|---|---|---|---|---|---|
| 11,938 | 51,307 | 9349099 | 452909 | 0,000000 | M | 11,477 | 12,789 | 51,307 |
| 13,698 | 48,693 | 8872862 | 346618 | 0,000000 | M | 13,216 | 14,731 | 48,693 |
| | 100,000 | 18221961 | 799527 | | | | | 100,000 |

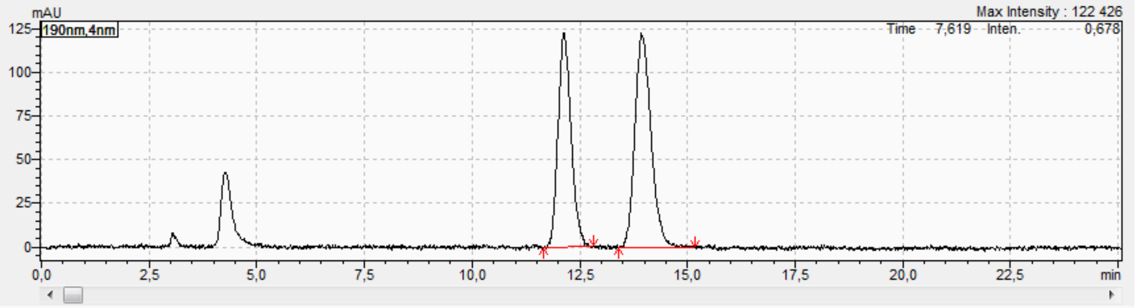


sults View - Peak Table

Compound | Group | Calibration Curve

| Ret. Time | Conc. | Area | Height | Similarity Index | Mark | Peak Start | Peak End | Area% |
|---|---|---|---|---|---|---|---|---|
| 12,146 | 44,770 | 2589769 | 122574 | 0,000000 | M | 11,648 | 12,821 | 44,770 |
| 13,931 | 55,230 | 3194814 | 122787 | 0,000000 | M | 13,408 | 15,168 | 55,230 |
| | 100,000 | 5784582 | 245361 | | | | | 100,000 |

**Conditions:** IG column, mobile phase: *n*-heptane/iPrOH 80:20, $\lambda$ = 199 nm, $V$ = 1.0 mL/min, $T$ = 25 °C, $t_R$ = 9.4 min (minor), $t_R$ = 10.1 min (major), *ee* 76%.

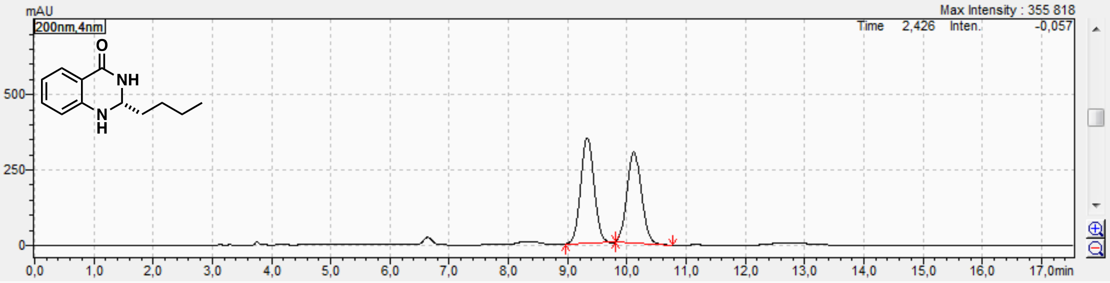


sults View - Peak Table

Compound | Group | Calibration Curve

| Ret. Time | Conc. | Area | Height | Similarity Index | Mark | Peak Start | Peak End | Area% |
|---|---|---|---|---|---|---|---|---|
| 9,330 | 51,496 | 5478119 | 348411 | 0,000000 | M | 8,960 | 9,803 | 51,496 |
| 10,118 | 48,504 | 5159820 | 301926 | 0,000000 | M | 9,803 | 10,773 | 48,504 |
| | 100,000 | 10637939 | 650337 | | | | | 100,000 |

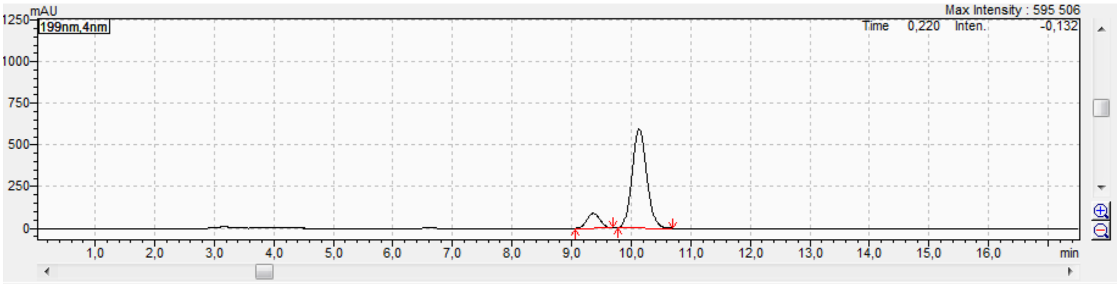


sults View - Peak Table

Compound | Group | Calibration Curve

| Ret. Time | Conc. | Area | Height | Similarity Index | Mark | Peak Start | Peak End | Area% |
|---|---|---|---|---|---|---|---|---|
| 9,363 | 11,916 | 1364777 | 88700 | 0,000000 | M | 9,067 | 9,696 | 11,916 |
| 10,134 | 88,084 | 10088601 | 593007 | 0,000000 | M | 9,781 | 10,688 | 88,084 |
| | 100,000 | 11453377 | 681707 | | | | | 100,000 |

**Conditions:** AD-H column, mobile phase: *n*-heptane/iPrOH 80:20, $\lambda$ = 220 nm, $V$ = 1.0 mL/min, $T$ = 25 °C, $t_R$ = 11.73 min (minor), $t_R$ = 13.79 min (major), *ee* 68%.

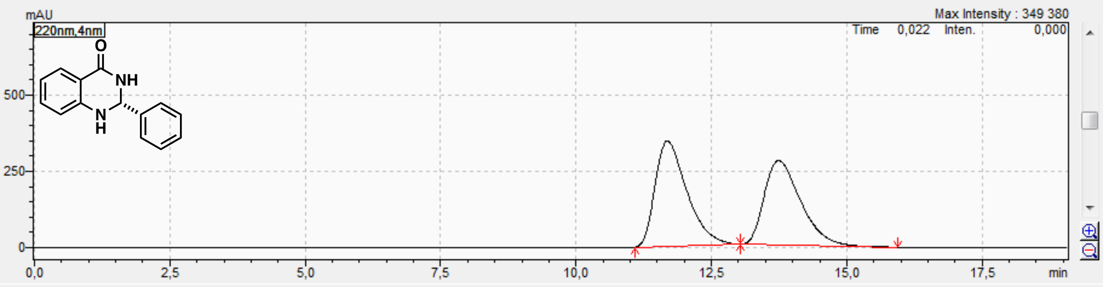


sults View - Peak Table

Compound | Group | Calibration Curve

| Ret. Time | Conc. | Area | Height | Similarity Index | Mark | Peak Start | Peak End | Area% |
|---|---|---|---|---|---|---|---|---|
| 11,687 | 52,280 | 14160187 | 345184 | 0,000000 | M | 11,093 | 13,035 | 52,280 |
| 13,743 | 47,720 | 12925118 | 277372 | 0,000000 | M | 13,035 | 15,947 | 47,720 |
| | 100,000 | 27085305 | 622556 | | | | | 100,000 |

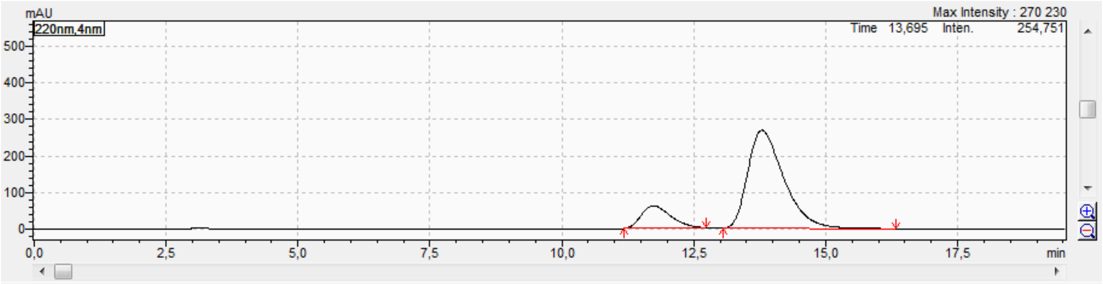


ults View - Peak Table

Compound | Group | Calibration Curve

| Ret. Time | Conc. | Area | Height | Similarity Index | Mark | Peak Start | Peak End | Area% |
|---|---|---|---|---|---|---|---|---|
| 11,733 | 15,814 | 2399670 | 61631 | 0,000000 | M | 11,179 | 12,725 | 15,814 |
| 13,793 | 84,186 | 12774769 | 268849 | 0,000000 | M | 13,056 | 16,320 | 84,186 |
| | 100,000 | 15174439 | 330479 | | | | | 100,000 |

**Conditions:** IA column, mobile phase: *n*-heptane/iPrOH 90:10, $\lambda$ = 190 nm, $V$ = 1.0 mL/min, $T$ = 25 °C, $t_R$ = 25.9 min (minor), $t_R$ = 29.8 min (major), *ee* 70% (after recrystallization 97%).

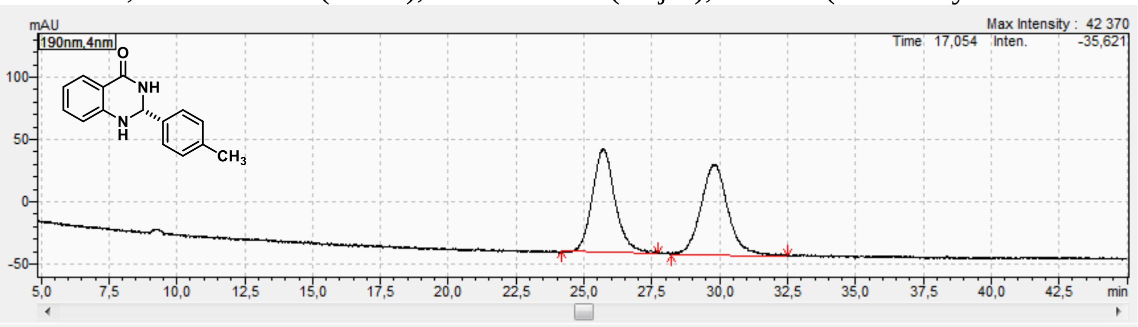


sults View - Peak Table

Compound | Group | Calibration Curve

| Ret. Time | Conc. | Area | Height | Similarity Index | Mark | Peak Start | Peak End | Area% |
|---|---|---|---|---|---|---|---|---|
| 25,687 | 49,020 | 4802710 | 82677 | 0,000000 | M | 24,181 | 27,701 | 49,020 |
| 29,834 | 50,980 | 4994772 | 72549 | 0,000000 | M | 28,192 | 32,512 | 50,980 |
| | 100,000 | 9797483 | 155226 | | | | | 100,000 |

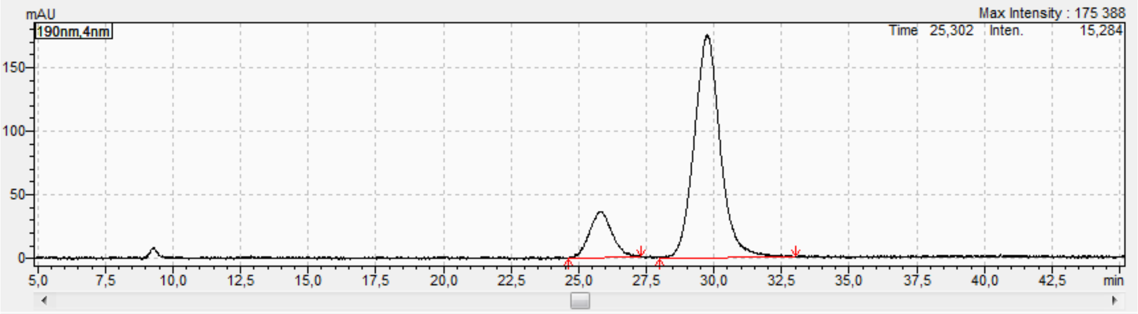


ults View - Peak Table

Compound | Group | Calibration Curve

| Ret. Time | Conc. | Area | Height | Similarity Index | Mark | Peak Start | Peak End | Area% |
|---|---|---|---|---|---|---|---|---|
| 25,871 | 15,379 | 2100959 | 36230 | 0,000000 | M | 24,597 | 27,307 | 15,379 |
| 29,753 | 84,621 | 11560190 | 174359 | 0,000000 | M | 28,000 | 33,013 | 84,621 |
| | 100,000 | 13661149 | 210588 | | | | | 100,000 |

After crystallization of **3f**.

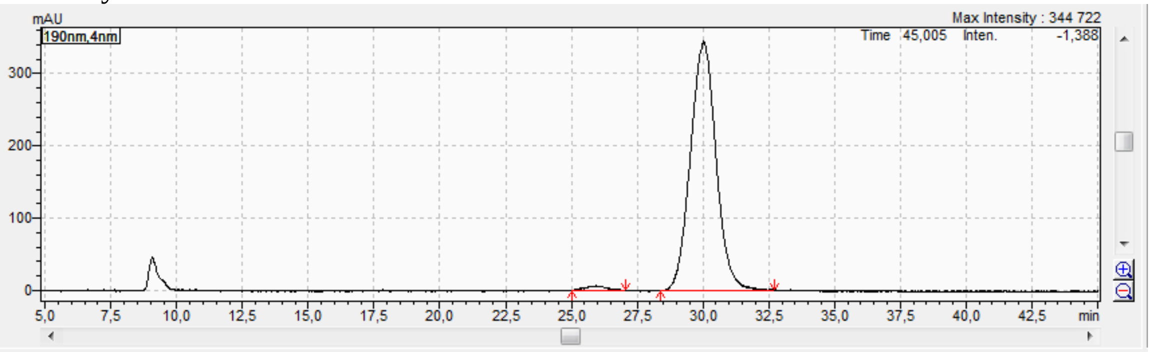


sults View - Peak Table

Compound | Group | Calibration Curve

| Ret. Time | Conc. | Area | Height | Similarity Index | Mark | Peak Start | Peak End | Area% |
|---|---|---|---|---|---|---|---|---|
| 25,922 | 1,373 | 322401 | 6401 | 0,000000 | M | 25,024 | 27,040 | 1,373 |
| 30,029 | 98,627 | 23164015 | 344118 | 0,000000 | M | 28,405 | 32,704 | 98,627 |
| | 100,000 | 23486416 | 350519 | | | | | 100,000 |

**Conditions:** IA column, mobile phase: *n*-heptane/iPrOH 90:10, $\lambda$ = 225 nm, $V$ = 1.0 mL/min, $T$ = 25 °C, $t_R$ = 21.2 min (minor), $t_R$ = 24.6 min (major), *ee* 36%.

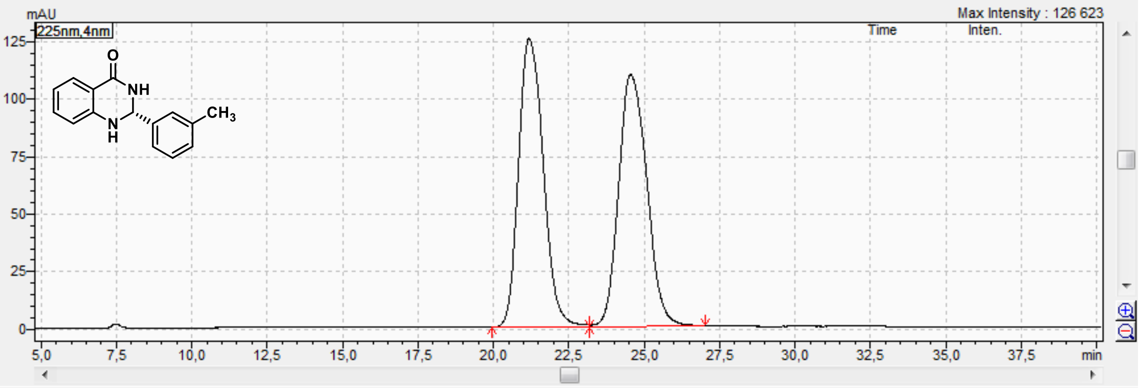


ults View - Peak Table

Compound | Group | Calibration Curve

| Ret. Time | Conc. | Area | Height | Similarity Index | Mark | Peak Start | Peak End | Area% |
|---|---|---|---|---|---|---|---|---|
| 21,183 | 50,075 | 7155863 | 125631 | 0,000000 | | 19,936 | 23,189 | 50,075 |
| 24,553 | 49,925 | 7134535 | 109552 | 0,000000 | V | 23,189 | 27,040 | 49,925 |
| | 100,000 | 14290398 | 235183 | | | | | 100,000 |

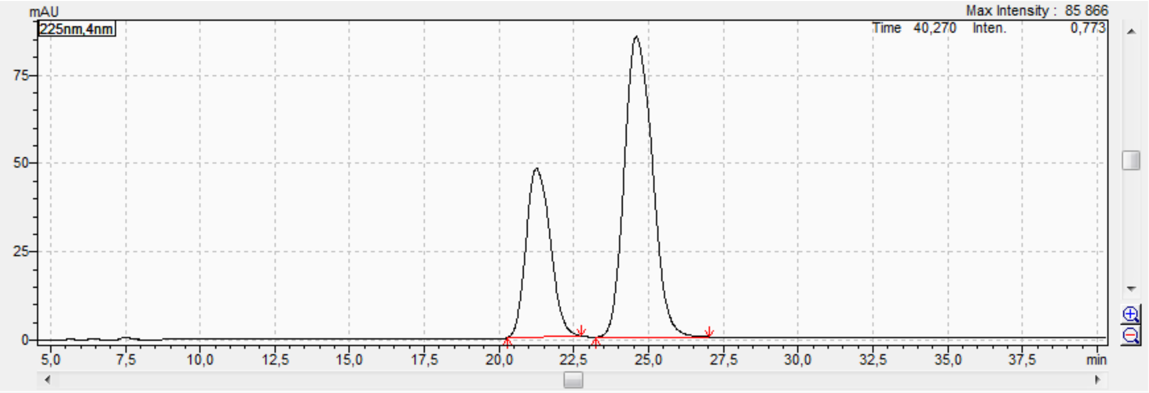


sults View - Peak Table

Compound | Group | Calibration Curve

| Ret. Time | Conc. | Area | Height | Similarity Index | Mark | Peak Start | Peak End | Area% |
|---|---|---|---|---|---|---|---|---|
| 21,243 | 32,701 | 2687455 | 47680 | 0,000000 | M | 20,256 | 22,741 | 32,701 |
| 24,584 | 67,299 | 5530935 | 84942 | 0,000000 | | 23,243 | 27,029 | 67,299 |
| | 100,000 | 8218390 | 132621 | | | | | 100,000 |

**Conditions:** IA column, mobile phase: *n*-heptane/iPrOH 90:10, $\lambda$ = 225 nm, $V$ = 1.0 mL/min, $T$ = 25 °C, $t_R$ = 21.2 min (minor), $t_R$ = 31.9 min (major), *ee* 20%.

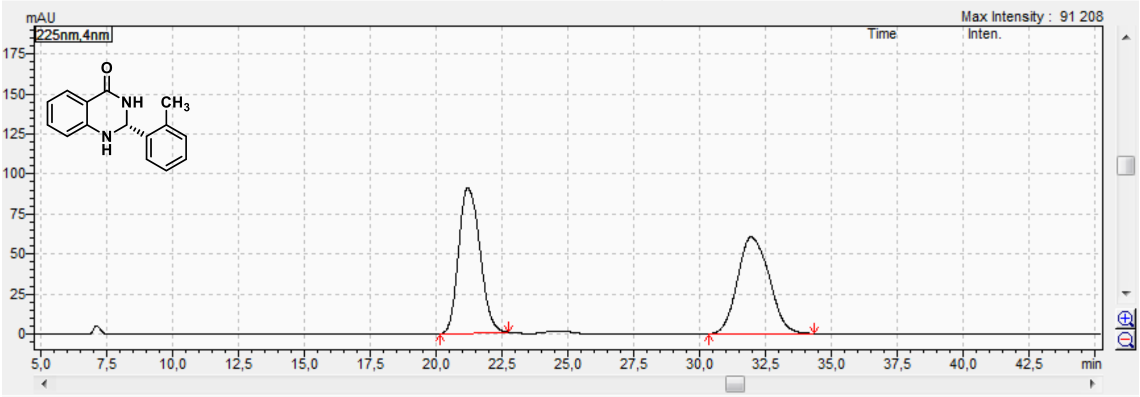


ults View - Peak Table

Compound | Group | Calibration Curve

| Ret. Time | Conc. | Area | Height | Similarity Index | Mark | Peak Start | Peak End | Area% |
|---|---|---|---|---|---|---|---|---|
| 21,208 | 50,363 | 5131901 | 90695 | 0,000000 | M | 20,171 | 22,773 | 50,363 |
| 31,970 | 49,637 | 5057994 | 60491 | 0,000000 | M | 30,368 | 34,389 | 49,637 |
| | 100,000 | 10189895 | 151186 | | | | | 100,000 |

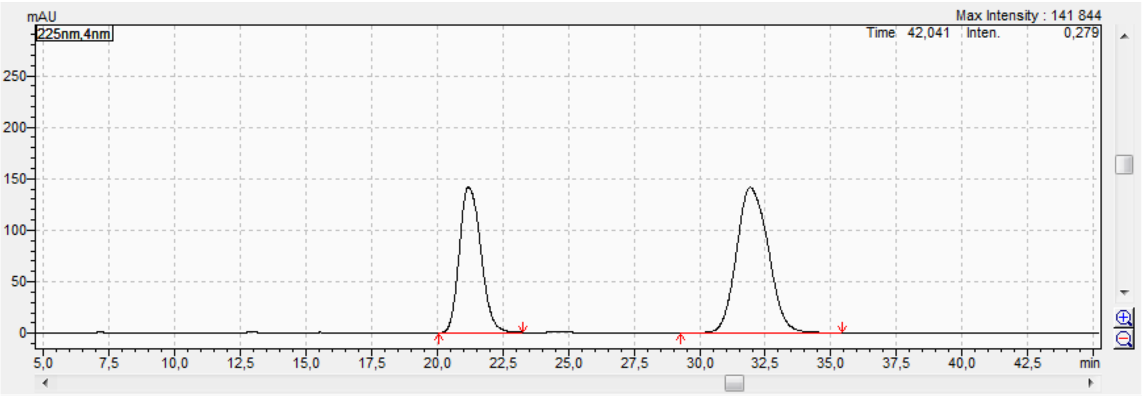


ults View - Peak Table

Compound | Group | Calibration Curve

| Ret. Time | Conc. | Area | Height | Similarity Index | Mark | Peak Start | Peak End | Area% |
|---|---|---|---|---|---|---|---|---|
| 21,191 | 40,374 | 8070113 | 141240 | 0,000000 | M | 20,053 | 23,275 | 40,374 |
| 31,928 | 59,626 | 11918067 | 141136 | 0,000000 | M | 29,280 | 35,424 | 59,626 |
| | 100,000 | 19988180 | 282376 | | | | | 100,000 |

**Conditions:** IC column, mobile phase: *n*-heptane/iPrOH 90:10, $\lambda$ = 225 nm, $V$ = 1.0 mL/min, $T$ = 25 °C, $t_R$ = 12.2 min (major), $t_R$ = 14.5 min (minor), *ee* 48%.

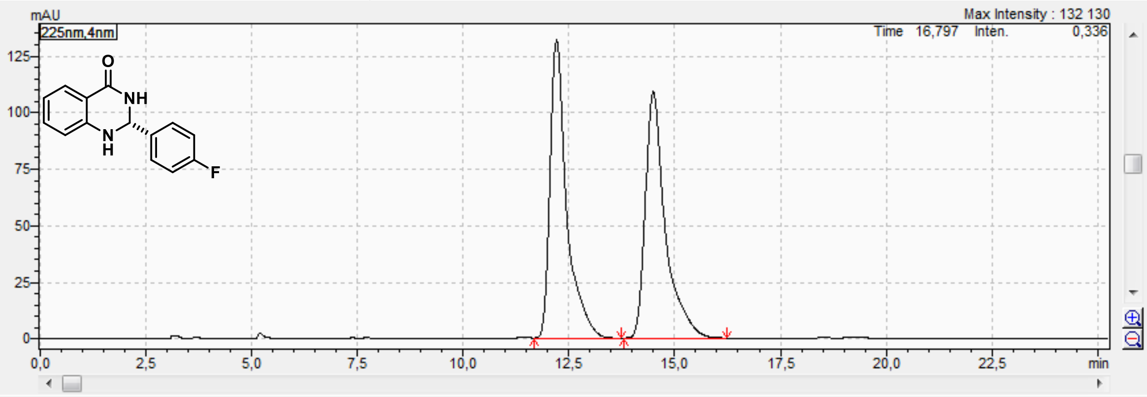


sults View - Peak Table

Compound | Group | Calibration Curve

| Ret. Time | Conc. | Area | Height | Similarity Index | Mark | Peak Start | Peak End | Area% |
|---|---|---|---|---|---|---|---|---|
| 12,208 | 50,006 | 3583743 | 131698 | 0,000000 | M | 11,680 | 13,728 | 50,006 |
| 14,494 | 49,994 | 3582870 | 108825 | 0,000000 | M | 13,792 | 16,245 | 49,994 |
| | 100,000 | 7166613 | 240522 | | | | | 100,000 |

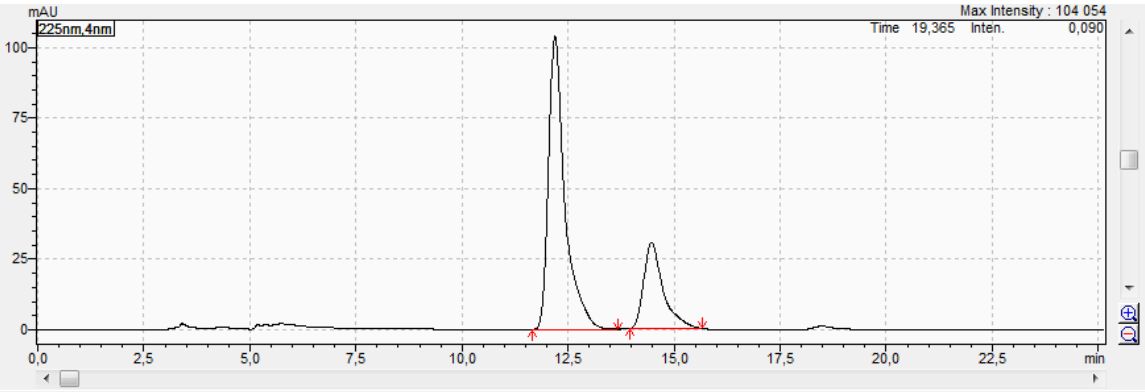


sults View - Peak Table

Compound | Group | Calibration Curve

| Ret. Time | Conc. | Area | Height | Similarity Index | Mark | Peak Start | Peak End | Area% |
|---|---|---|---|---|---|---|---|---|
| 12,192 | 74,033 | 2783726 | 103881 | 0,000000 | M | 11,669 | 13,675 | 74,033 |
| 14,470 | 25,967 | 976368 | 30641 | 0,000000 | M | 13,941 | 15,669 | 25,967 |
| | 100,000 | 3760094 | 134522 | | | | | 100,000 |

**Conditions:** IA column, mobile phase: *n*-heptane/iPrOH 90:10, $\lambda$ = 224 nm, $V$ = 1.0 mL/min, $T$ = 25 °C, $t_R$ = 10.3 min (minor), $t_R$ = 13.0 min (major), *ee* 53%.

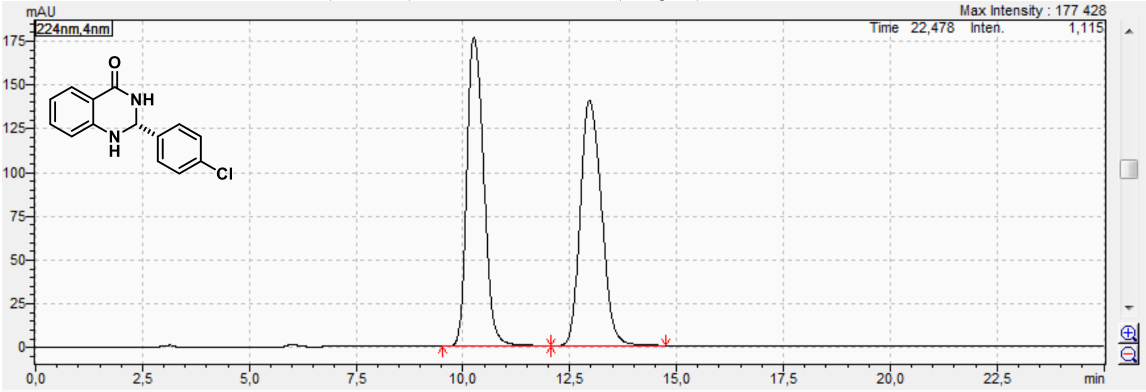


ults View - Peak Table

Compound | Group | Calibration Curve

| Ret. Time | Conc. | Area | Height | Similarity Index | Mark | Peak Start | Peak End | Area% |
|---|---|---|---|---|---|---|---|---|
| 10,259 | 50,093 | 4860040 | 176647 | 0,000000 | S | 9,536 | 12,064 | 50,093 |
| 12,962 | 49,907 | 4842024 | 139899 | 0,000000 | V | 12,064 | 14,741 | 49,907 |
| | 100,000 | 9702064 | 316546 | | | | | 100,000 |

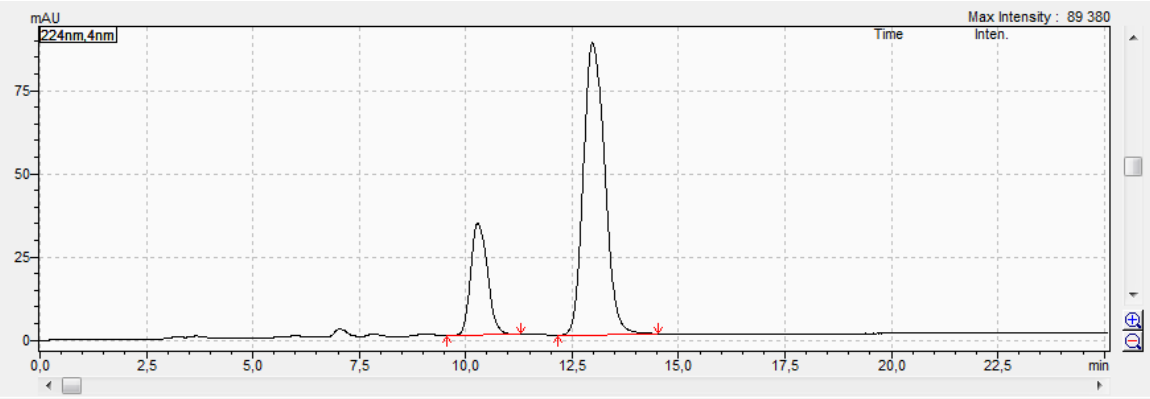


sults View - Peak Table

Compound | Group | Calibration Curve

| Ret. Time | Conc. | Area | Height | Similarity Index | Mark | Peak Start | Peak End | Area% |
|---|---|---|---|---|---|---|---|---|
| 10,285 | 23,279 | 928460 | 33635 | 0,000000 | | 9,568 | 11,285 | 23,279 |
| 12,981 | 76,721 | 3059905 | 87698 | 0,000000 | | 12,171 | 14,507 | 76,721 |
| | 100,000 | 3988365 | 121333 | | | | | 100,000 |

**Conditions:** IA column, mobile phase: *n*-heptane/iPrOH 80:20, $\lambda$ = 190 nm, $V$ = 1.0 mL/min, $T$ = 25 °C, $t_R$ = 5.0 min (minor), $t_R$ = 5.9 min (major), *ee* 30%.

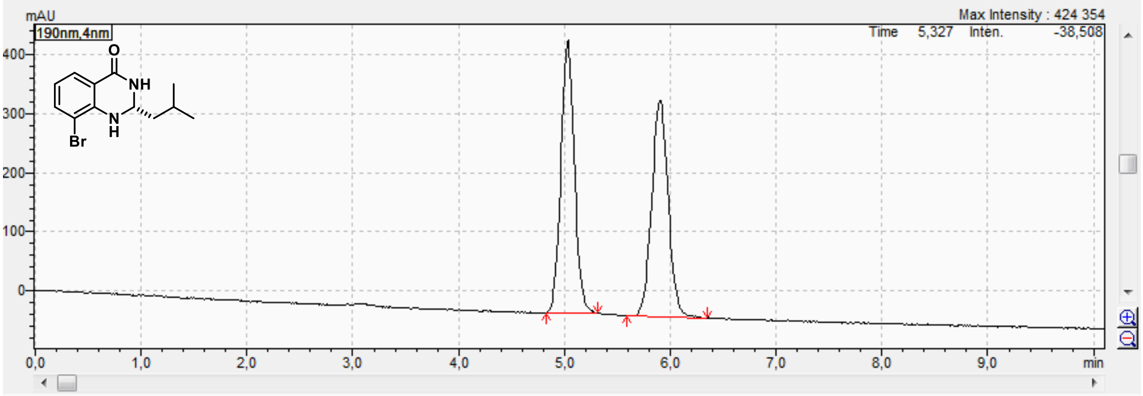


lts View - Peak Table

Compound | Group | Calibration Curve

| Ret. Time | Conc. | Area | Height | Similarity Index | Mark | Peak Start | Peak End | Area% |
|---|---|---|---|---|---|---|---|---|
| 5,032 | 50,826 | 4075931 | 461743 | 0,000000 | M | 4,832 | 5,312 | 50,826 |
| 5,905 | 49,174 | 3943441 | 366059 | 0,000000 | M | 5,589 | 6,347 | 49,174 |
| | 100,000 | 8019372 | 827802 | | | | | 100,000 |

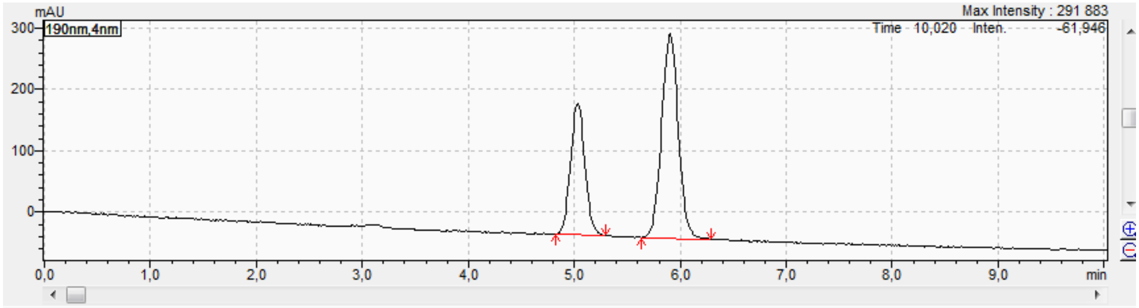


sults View - Peak Table

Compound | Group | Calibration Curve

| Ret. Time | Conc. | Area | Height | Similarity Index | Mark | Peak Start | Peak End | Area% |
|---|---|---|---|---|---|---|---|---|
| 5,033 | 35,428 | 2005040 | 213347 | 0,000000 | M | 4,821 | 5,291 | 35,428 |
| 5,906 | 64,572 | 3654427 | 334356 | 0,000000 | M | 5,632 | 6,293 | 64,572 |
| | 100,000 | 5659468 | 547703 | | | | | 100,000 |

**Conditions:** IA column, mobile phase: *n*-heptane/iPrOH 80:20, *V* = 1.0 mL/min, λ = 190 nm, *T* = 25 °C, $t_R$ = 7.3 min (minor), $t_R$ = 8.2 min (major), *ee* 70% (80% after recrystallization) .

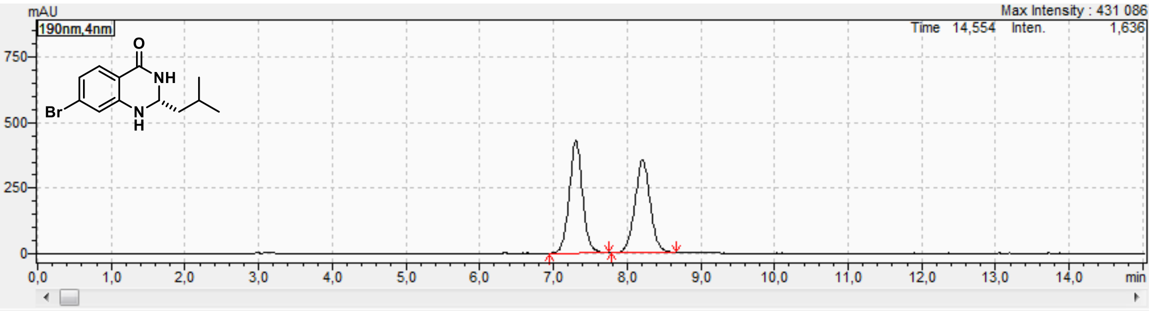


sults View - Peak Table

Compound | Group | Calibration Curve

| Ret. Time | Conc. | Area | Height | Similarity Index | Mark | Peak Start | Peak End | Area% |
|---|---|---|---|---|---|---|---|---|
| 7,305 | 50,834 | 5323574 | 427639 | 0,000000 | M | 6,944 | 7,744 | 50,834 |
| 8,213 | 49,166 | 5148989 | 355116 | 0,000000 | M | 7,797 | 8,672 | 49,166 |
| | 100,000 | 10472563 | 782755 | | | | | 100,000 |

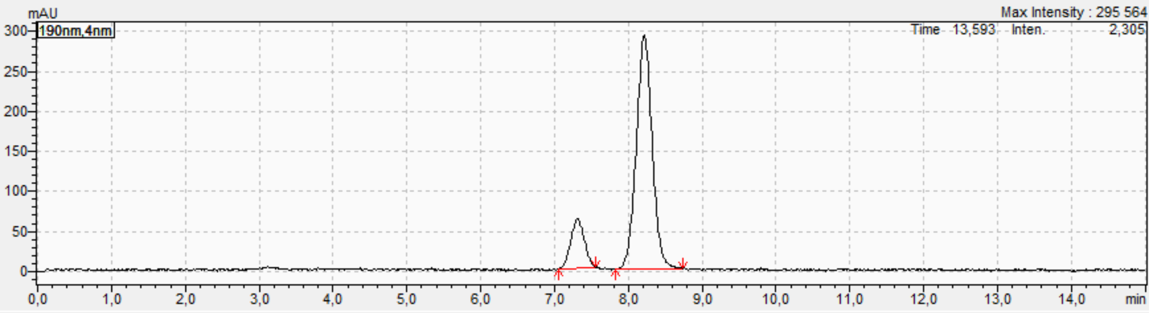


sults View - Peak Table

Compound | Group | Calibration Curve

| Ret. Time | Conc. | Area | Height | Similarity Index | Mark | Peak Start | Peak End | Area% |
|---|---|---|---|---|---|---|---|---|
| 7,310 | 15,296 | 782109 | 61238 | 0,000000 | M | 7,061 | 7,552 | 15,296 |
| 8,216 | 84,704 | 4330886 | 292021 | 0,000000 | M | 7,829 | 8,747 | 84,704 |
| | 100,000 | 5112995 | 353260 | | | | | 100,000 |

After recrystallization of **3l**.

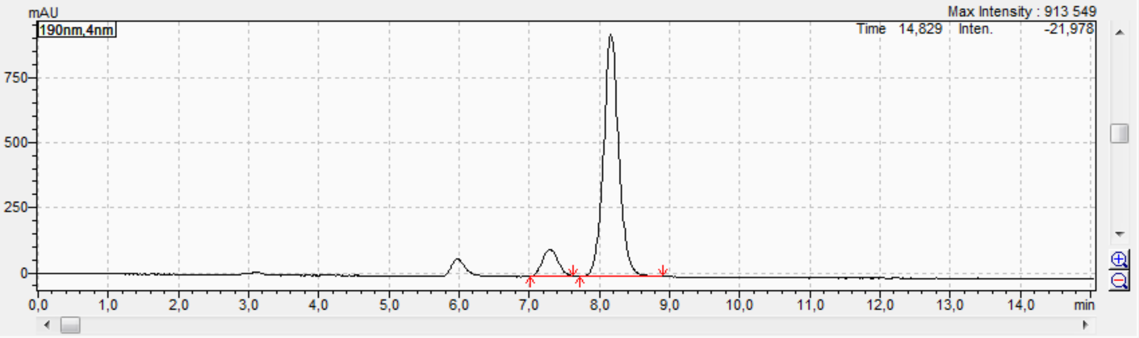


sults View - Peak Table

Compound | Group | Calibration Curve

| Ret. Time | Conc. | Area | Height | Similarity Index | Mark | Peak Start | Peak End | Area% |
|---|---|---|---|---|---|---|---|---|
| 7,289 | 9,904 | 1554510 | 101980 | 0,000000 | M | 7,019 | 7,616 | 9,904 |
| 8,162 | 90,096 | 14140617 | 927410 | 0,000000 | M | 7,712 | 8,896 | 90,096 |
| | 100,000 | 15695127 | 1029390 | | | | | 100,000 |

**Conditions:** OD-H column, mobile phase: *n*-heptane/iPrOH 90:10, $\lambda$ = 190 nm, $V$ = 1.0 mL/min, $T$ = 25 °C, $t_R$ = 9.7 min (minor), $t_R$ = 14.0 min (major), *ee* 80%.

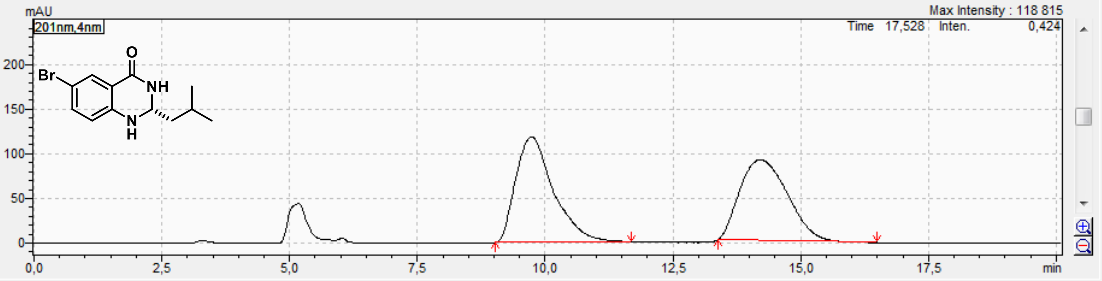


sults View - Peak Table

Compound | Group | Calibration Curve

| Ret. Time | Conc. | Area | Height | Similarity Index | Mark | Peak Start | Peak End | Area% |
|---|---|---|---|---|---|---|---|---|
| 9,734 | 50,824 | 6049318 | 117772 | 0,000000 | M | 9,024 | 11,680 | 50,824 |
| 14,207 | 49,176 | 5853204 | 90291 | 0,000000 | M | 13,387 | 16,491 | 49,176 |
| | 100,000 | 11902522 | 208063 | | | | | 100,000 |

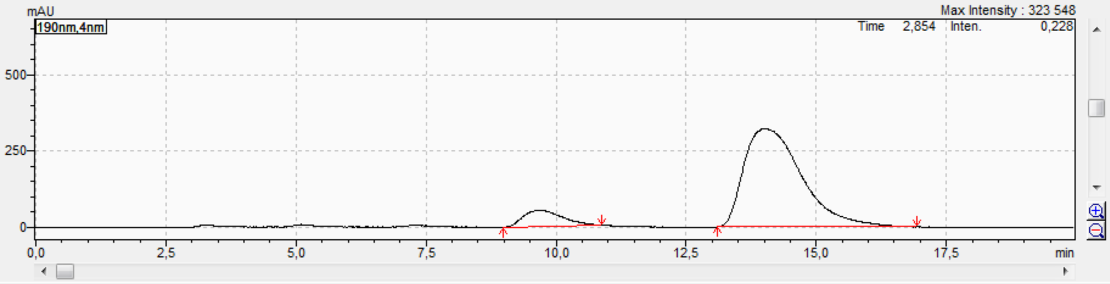


sults View - Peak Table

Compound | Group | Calibration Curve

| Ret. Time | Conc. | Area | Height | Similarity Index | Mark | Peak Start | Peak End | Area% |
|---|---|---|---|---|---|---|---|---|
| 9,673 | 9,934 | 2747274 | 52402 | 0,000000 | M | 8,971 | 10,869 | 9,934 |
| 14,015 | 90,066 | 24907109 | 322399 | 0,000000 | M | 13,088 | 16,928 | 90,066 |
| | 100,000 | 27654382 | 374801 | | | | | 100,000 |

**Conditions:** IA column, mobile phase: *n*-heptane/iPrOH 80:10, $V$ = 1.0 mL/min, $\lambda$ = 190 nm, $T$ = 25 °C, $t_R$ = 6.0 min (minor), $t_R$ = 6.7 min (major), *ee* 66%.

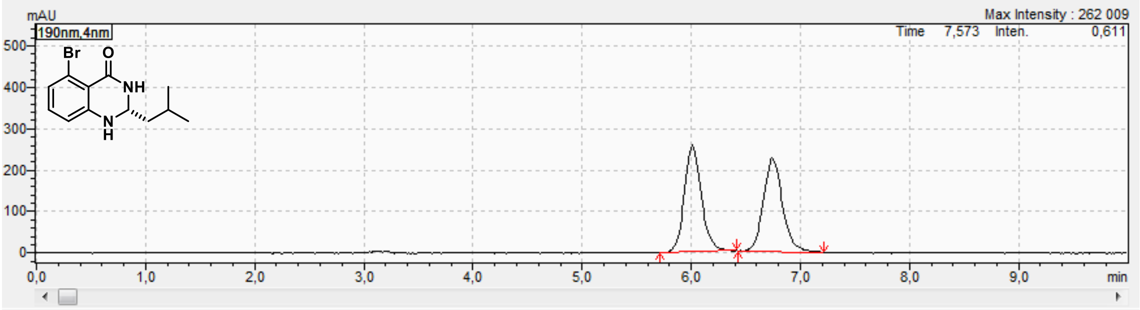


ults View - Peak Table

Compound | Group | Calibration Curve

| Ret. Time | Conc. | Area | Height | Similarity Index | Mark | Peak Start | Peak End | Area% |
|---|---|---|---|---|---|---|---|---|
| 6,010 | 50,728 | 2804441 | 259462 | 0,000000 | M | 5,717 | 6,411 | 50,728 |
| 6,743 | 49,272 | 2723998 | 226359 | 0,000000 | M | 6,432 | 7,211 | 49,272 |
| | 100,000 | 5528439 | 485821 | | | | | 100,000 |

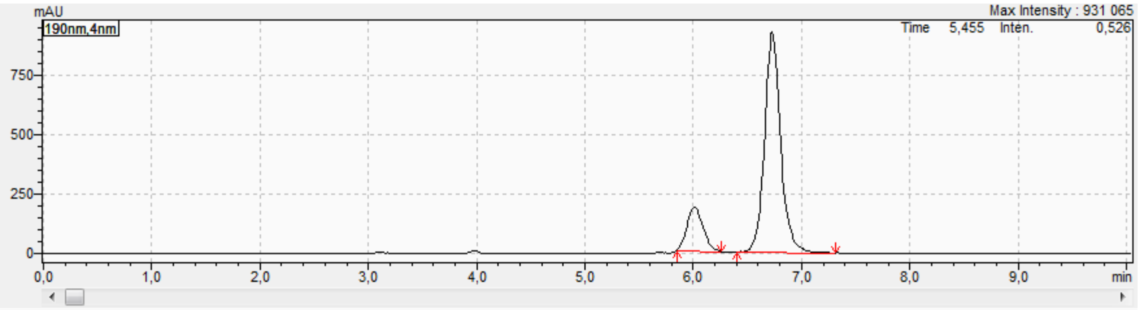


esults View - Peak Table

e | Compound | Group | Calibration Curve

| Ret. Time | Conc. | Area | Height | Similarity Index | Mark | Peak Start | Peak End | Area% |
|---|---|---|---|---|---|---|---|---|
| 6,008 | 16,674 | 1899964 | 183084 | 0,000000 | M | 5,845 | 6,261 | 16,674 |
| 6,730 | 83,326 | 9494655 | 925771 | 0,000000 | M | 6,400 | 7,317 | 83,326 |
| | 100,000 | 11394619 | 1108855 | | | | | 100,000 |

**Conditions:** OD-H column, mobile phase: *n*-heptane/iPrOH 90:10, $\lambda$ = 223 nm, $V$ = 1.0 mL/min, $T$ = 25 °C, $t_R$ = 9.2 min (minor), $t_R$ = 13.0 min (major), *ee* 76%.

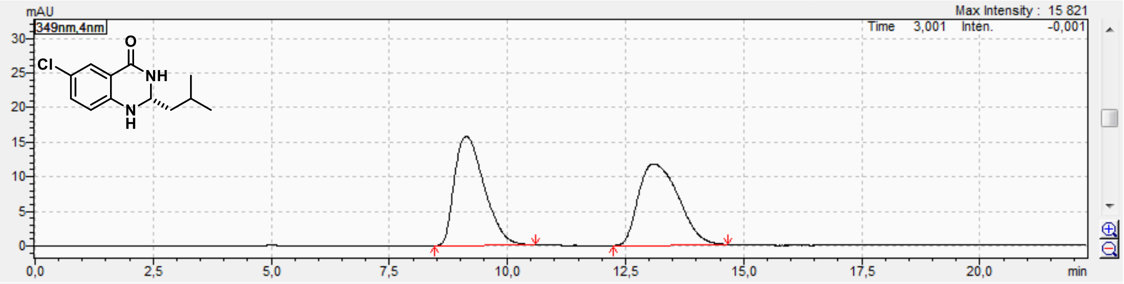


sults View - Peak Table

Compound | Group | Calibration Curve

| Ret. Time | Conc. | Area | Height | Similarity Index | Mark | Peak Start | Peak End | Area% |
|---|---|---|---|---|---|---|---|---|
| 9,130 | 50,135 | 693530 | 15756 | 0,000000 | | 8,459 | 10,603 | 50,135 |
| 13,096 | 49,865 | 689787 | 11752 | 0,000000 | | 12,245 | 14,677 | 49,865 |
| | 100,000 | 1383317 | 27508 | | | | | 100,000 |

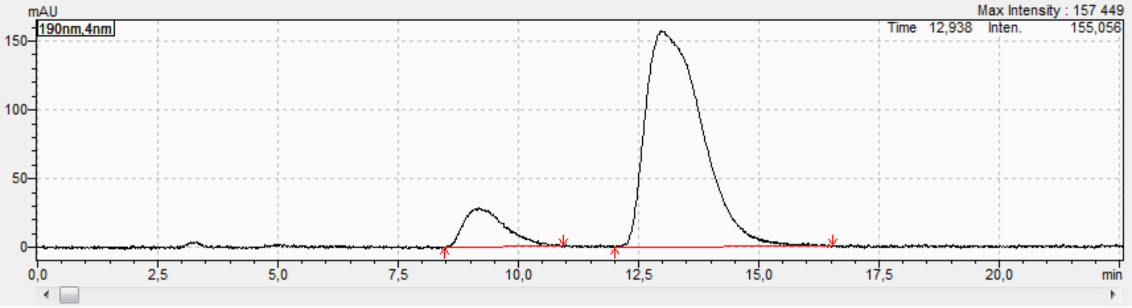


sults View - Peak Table

Compound | Group | Calibration Curve

| Ret. Time | Conc. | Area | Height | Similarity Index | Mark | Peak Start | Peak End | Area% |
|---|---|---|---|---|---|---|---|---|
| 9,164 | 11,752 | 1694422 | 28598 | 0,000000 | M | 8,459 | 10,933 | 11,752 |
| 12,971 | 88,248 | 12723793 | 156786 | 0,000000 | M | 12,000 | 16,533 | 88,248 |
| | 100,000 | 14418215 | 185385 | | | | | 100,000 |

**Conditions:** IG column, mobile phase: *n*-heptane/iPrOH 80:20, $\lambda$ = 190 nm, $V$ = 1.0 mL/min, $T$ = 25 °C, $t_R$ = 7.0 min (major), $t_R$ = 8.0 min (minor) *ee* 36%.

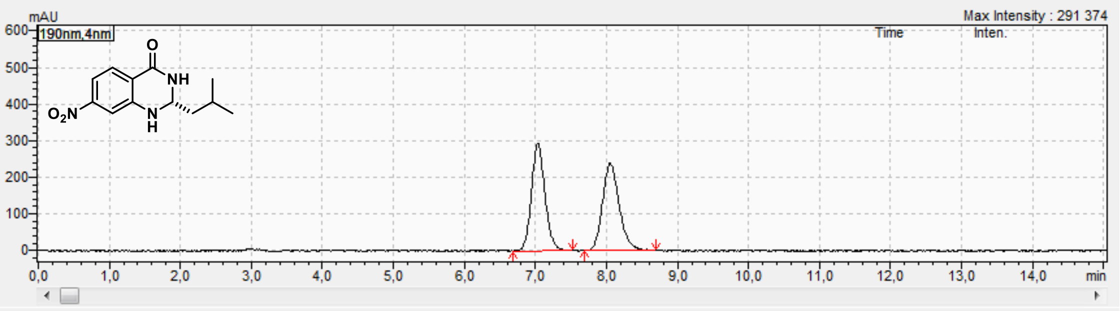


sults View - Peak Table

Compound | Group | Calibration Curve

| Ret. Time | Conc. | Area | Height | Similarity Index | Mark | Peak Start | Peak End | Area% |
|---|---|---|---|---|---|---|---|---|
| 7,035 | 49,915 | 3854976 | 292065 | 0,000000 | M | 6,688 | 7,531 | 49,915 |
| 8,053 | 50,085 | 3868169 | 238646 | 0,000000 | M | 7,691 | 8,693 | 50,085 |
| | 100,000 | 7723145 | 530711 | | | | | 100,000 |

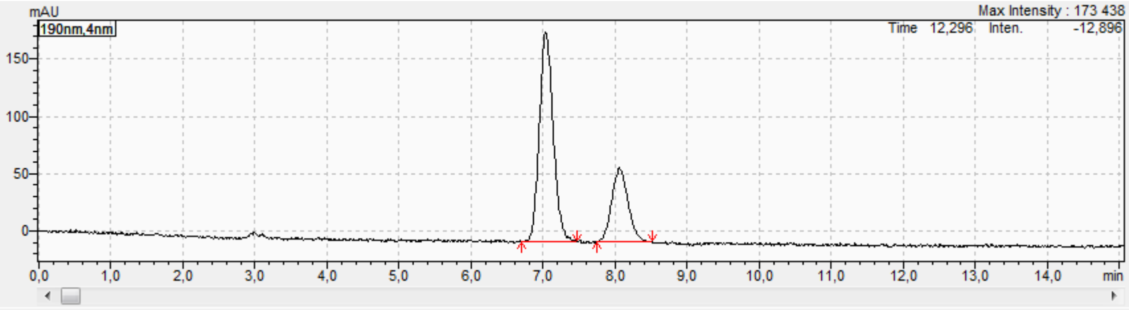


sults View - Peak Table

Compound | Group | Calibration Curve

| Ret. Time | Conc. | Area | Height | Similarity Index | Mark | Peak Start | Peak End | Area% |
|---|---|---|---|---|---|---|---|---|
| 7,033 | 71,023 | 2468521 | 183190 | 0,000000 | M | 6,699 | 7,467 | 71,023 |
| 8,060 | 28,977 | 1007147 | 64832 | 0,000000 | M | 7,744 | 8,512 | 28,977 |
| | 100,000 | 3475669 | 248022 | | | | | 100,000 |

**Conditions:** IG column, mobile phase: *n*-heptane/iPrOH 80:20, $\lambda$ = 223 nm, $V$ = 1.0 mL/min, $T$ = 25 °C, $t_R$ = 16.76 min (minor), $t_R$ = 18.28 min (major), *ee* 69%.

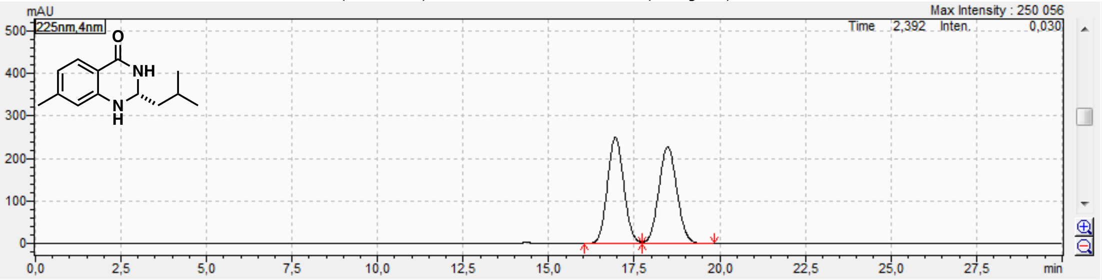


sults View - Peak Table

Compound | Group | Calibration Curve

| Ret. Time | Conc. | Area | Height | Similarity Index | Mark | Peak Start | Peak End | Area% |
|---|---|---|---|---|---|---|---|---|
| 16,949 | 49,791 | 8315227 | 249925 | 0,000000 | | 16,032 | 17,728 | 49,791 |
| 18,477 | 50,209 | 8384995 | 227446 | 0,000000 | V | 17,728 | 19,840 | 50,209 |
| | 100,000 | 16700223 | 477371 | | | | | 100,000 |

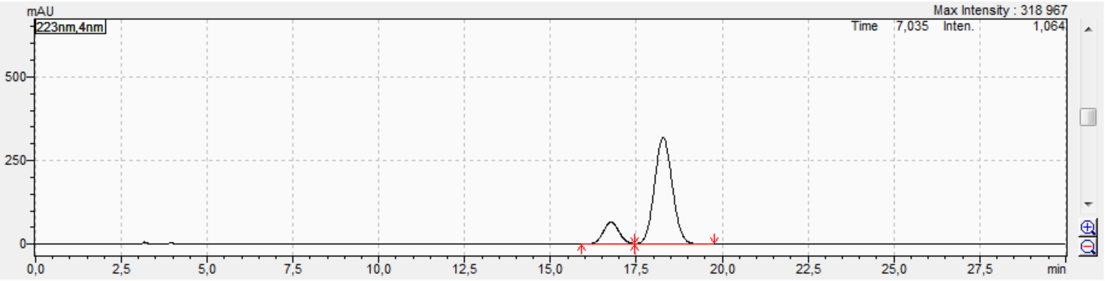


sults View - Peak Table

Compound | Group | Calibration Curve

| Ret. Time | Conc. | Area | Height | Similarity Index | Mark | Peak Start | Peak End | Area% |
|---|---|---|---|---|---|---|---|---|
| 16,761 | 15,710 | 2150824 | 65984 | 0,000000 | | 15,904 | 17,461 | 15,710 |
| 18,280 | 84,290 | 11539565 | 318829 | 0,000000 | V | 17,461 | 19,765 | 84,290 |
| | 100,000 | 13690388 | 384813 | | | | | 100,000 |

**Conditions:** OD-H column, mobile phase: *n*-heptane/iPrOH 80:20, $\lambda$ = 220 nm, $V$ = 1.0 mL/min, $T$ = 25 °C, $t_R$ = 7.18 min (minor), $t_R$ = 9.47 min (major), *ee* 72%.

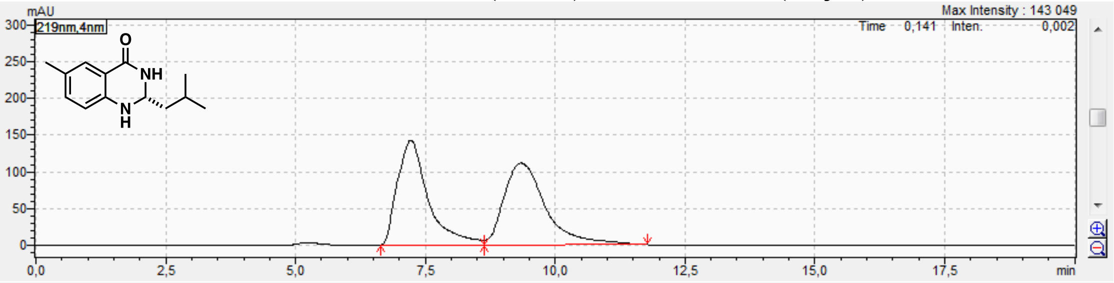


sults View - Peak Table

Compound | Group | Calibration Curve

| Ret. Time | Conc. | Area | Height | Similarity Index | Mark | Peak Start | Peak End | Area% |
|---|---|---|---|---|---|---|---|---|
| 7,217 | 48,577 | 5853335 | 142191 | 0,000000 | M | 6,635 | 8,629 | 48,577 |
| 9,341 | 51,423 | 6196185 | 110879 | 0,000000 | V M | 8,629 | 11,787 | 51,423 |
| | 100,000 | 12049519 | 253070 | | | | | 100,000 |

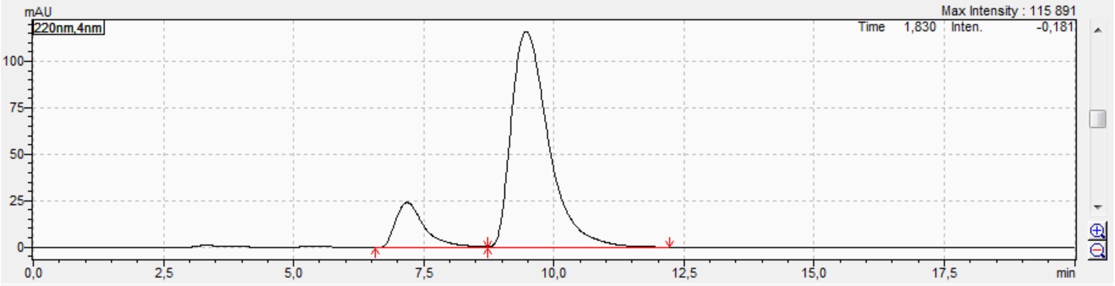


ults View - Peak Table

Compound | Group | Calibration Curve

| Ret. Time | Conc. | Area | Height | Similarity Index | Mark | Peak Start | Peak End | Area% |
|---|---|---|---|---|---|---|---|---|
| 7,183 | 13,994 | 938101 | 24193 | 0,000000 | | 6,571 | 8,725 | 13,994 |
| 9,466 | 86,006 | 5765648 | 116031 | 0,000000 | V | 8,725 | 12,224 | 86,006 |
| | 100,000 | 6703750 | 140224 | | | | | 100,000 |

**Conditions:** IG column, mobile phase: *n*-heptane/iPrOH 80:20, $\lambda$ = 190 nm, $V$ = 1.0 mL/min, $T$ = 25 °C, $t_R$ = 11.6 min (minor), $t_R$ = 13.0 min (major), *ee* 64%.

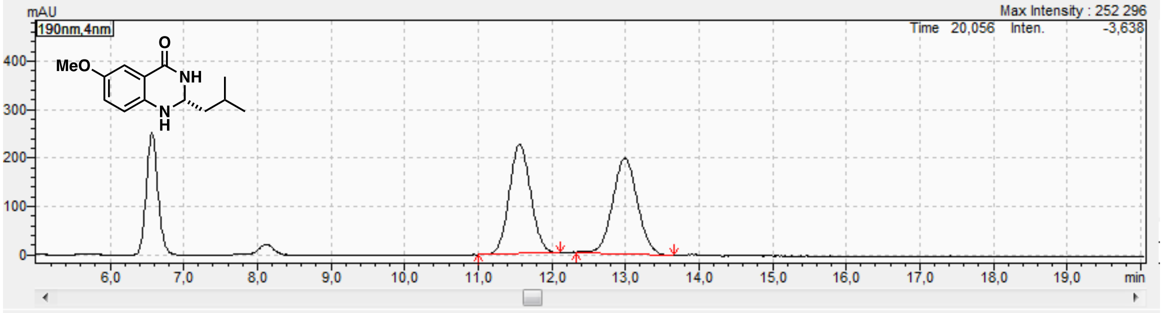


ults View - Peak Table

Compound | Group | Calibration Curve

| Ret. Time | Conc. | Area | Height | Similarity Index | Mark | Peak Start | Peak End | Area% |
|---|---|---|---|---|---|---|---|---|
| 11,567 | 49,961 | 4584257 | 226636 | 0,000000 | M | 10,997 | 12,117 | 49,961 |
| 12,993 | 50,039 | 4591378 | 197315 | 0,000000 | M | 12,331 | 13,664 | 50,039 |
| | 100,000 | 9175635 | 423951 | | | | | 100,000 |

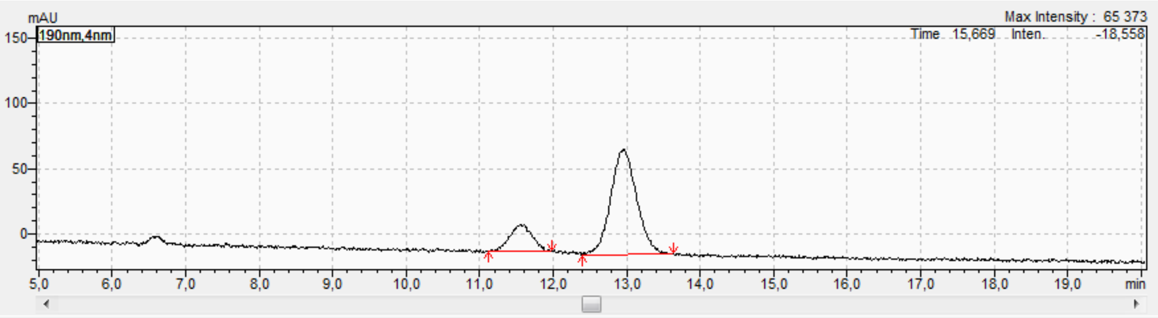


sults View - Peak Table

Compound | Group | Calibration Curve

| Ret. Time | Conc. | Area | Height | Similarity Index | Mark | Peak Start | Peak End | Area% |
|---|---|---|---|---|---|---|---|---|
| 11,546 | 18,071 | 433629 | 19967 | 0,000000 | M | 11,125 | 11,979 | 18,071 |
| 12,961 | 81,929 | 1966018 | 80800 | 0,000000 | M | 12,395 | 13,632 | 81,929 |
| | 100,000 | 2399647 | 100767 | | | | | 100,000 |

**Conditions:** IA column, mobile phase: *n*-heptane/iPrOH 80:20, $\lambda$ = 190 nm, $V$ = 1.0 mL/min, $T$ = 25 °C, $t_R$ = 7.0 min (major), $t_R$ = 10.7 min (minor), *ee* 35%.

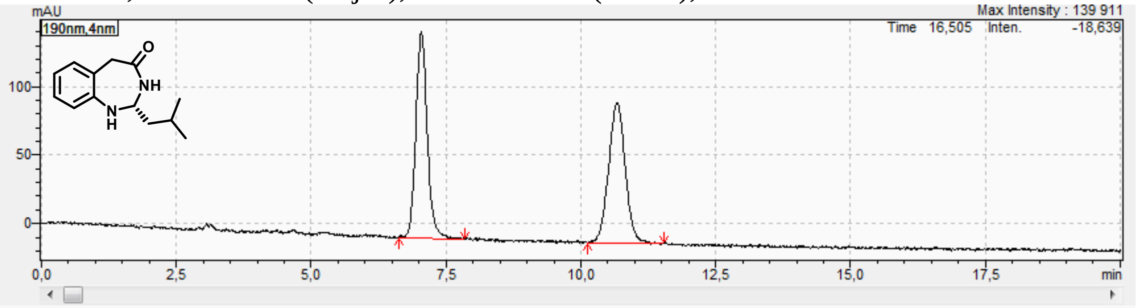


sults View - Peak Table

Compound | Group | Calibration Curve

| Ret. Time | Conc. | Area | Height | Similarity Index | Mark | Peak Start | Peak End | Area% |
|---|---|---|---|---|---|---|---|---|
| 7,043 | 50,158 | 2207270 | 150446 | 0,000000 | M | 6,624 | 7,851 | 50,158 |
| 10,670 | 49,842 | 2193357 | 102241 | 0,000000 | M | 10,133 | 11,541 | 49,842 |
| | 100,000 | 4400627 | 252687 | | | | | 100,000 |

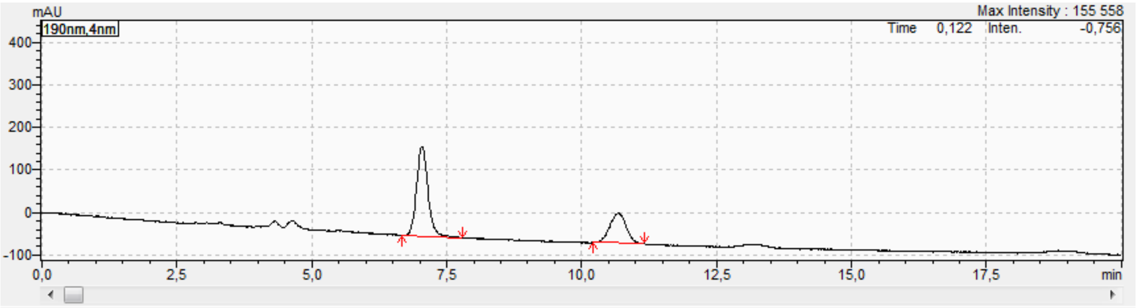


sults View - Peak Table

Compound | Group | Calibration Curve

| Ret. Time | Conc. | Area | Height | Similarity Index | Mark | Peak Start | Peak End | Area% |
|---|---|---|---|---|---|---|---|---|
| 7,044 | 67,453 | 3073730 | 210558 | 0,000000 | M | 6,667 | 7,787 | 67,453 |
| 10,671 | 32,547 | 1483129 | 70355 | 0,000000 | M | 10,208 | 11,157 | 32,547 |
| | 100,000 | 4556858 | 280913 | | | | | 100,000 |

# X-Ray section

The diffraction experiment for crystal structure determination was performed on a Bruker D8 VENTURE Kappa Duo with PHOTONIII detector by IµS micro-focus sealed tube with MoKα (0.71073) radiation at a temperature 120(2) K. The structure was solved by direct methods (XT) [8] and refined by full matrix least squares based on $F^2$ (SHELXL2018) [9]. The hydrogen atoms on carbon were fixed into idealized positions (riding model) and assigned temperature factors either Hiso(H) = 1.2 $U_{eq}$(pivot atom) or Hiso(H) = 1.5 $U_{eq}$(pivot atom) for methyl moiety, the hydrogen atoms in –N-H amoieties were found on difference Fourier maps and refined under rigid body assumption with assigned temperature factors $H_{iso}$(H) = 1.2 $U_{eq}$(pivot atom).

Crystal data for **3l**: $C_{12}H_{15}BrN_2O$; *Mr* = 283.17; monoclinic, $P2_1$ (No 4), *a* = 11.0816 (3) Å, *b* = 9.0888 (3) Å, *c* = 12.4473 (4) Å, *β* = 95.745 (1)°, *V* = 1247.38 (7) Å$^3$, Z = 4, $D_x$ = 1.508 Mg m$^{-3}$. Prism, colourless of dimensions 0.19 × 0.12 × 0.12 mm, multi-scan absorption correction (µ = 3.28 mm$^{-1}$) $T_{min}$ = 0.63, $T_{max}$ = 0.70; a total of 38831 measured reflections ($\theta_{max}$= 30˚), from which 7225 were unique ($R_{int}$ = 0.028) and 6671 observed according to the *I* > 2σ(*I*) criterion. The refinement converged (Δ/σmax= 0.002) to *R* = 0.022 for observed reflections and w$R$($F^2$) = 0.059, *GOF* = 1.14 for 293 parameters and all 7225 reflections. The final difference map displayed no peaks of chemical significance (Δρmax = 0.53, Δρmin -0.31 e.Å$^{-3}$).

The two symmetrically independent molecules fit each other well, with maximal deviation 0.7 Å between isopropyl moieties. The determination of absolute structure was based on anomalous scattering of bromine atom. Absolute structure parameter: -0.011 (2) [10].

X-ray crystallographic data have been deposited with the Cambridge Crystallographic Data Centre (CCDC) under deposition number **2081064** for **3h** and can be obtained free of charge from the Centre via its website (www.ccdc.cam.ac.uk/getstructures).

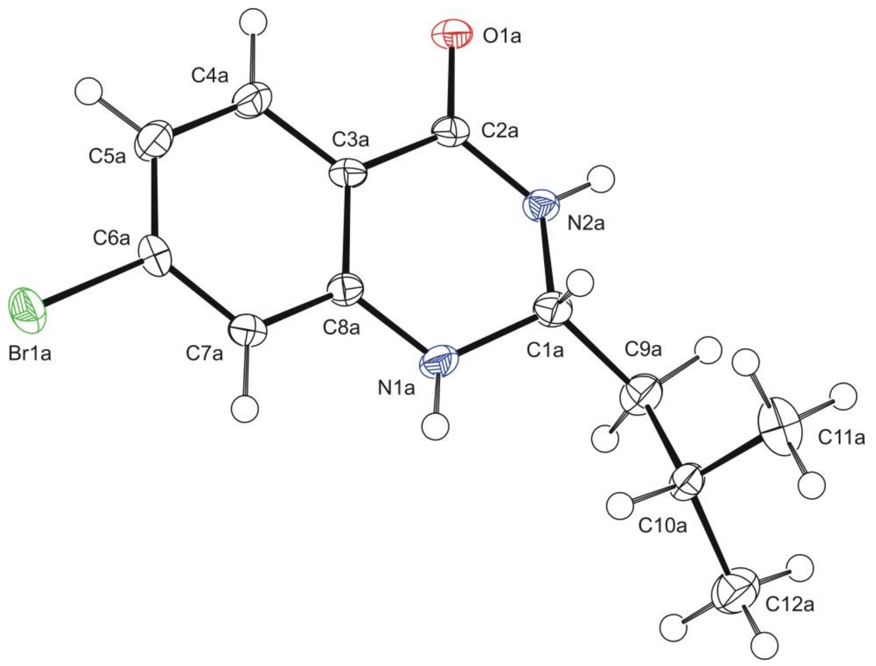


**Figure S1:** View on the one of two symmetrically independent molecules of **3l**. Displacement ellipsoids are drawn at 30% probability level. Two independent molecules fit one on other almost perfectly with maximal difference of corresponding atoms 0.275 Å.

# References


1. Gheewala, C. D.; Radtke, M. A.; Hui, J.; Hon, A. B.; Lambert, T. H. *Org. Lett.* **2017**, *19* (16), 4227–4230. https://doi.org/10.1021/acs.orglett.7b01867.
2. Parua, S.; Das, S.; Sikari, R.; Sinha, S.; Paul, N. D. *J. Org. Chem.* **2017**, *82*, (14), 7165–7175. https://doi.org/10.1021/acs.joc.7b00643.
3. Sutherell, C. L. et al. *J. Med. Chem.* **2016**, *59*, (10), 5095–5101. https://doi.org/10.1021/acs.jmedchem.5b01997.
4. Chen, Z. et al. *Org. Biomol. Chem.* **2020**, *18*, 8677–8685. https://doi.org/10.1039/D0OB01864C.
5. Hikawa, H.; Ino, Y.; Suzuki, H.; Yokoyama, Y. *J. Org. Chem.* **2012**, *77*, (16), 7046–7051. https://doi.org/10.1021/jo301282n.
6. Ma, X.; Lu, M. *J. Chem. Res.* **2011**, *35*, (8), 480–483. https://doi.org/10.3184/174751911X13133294115830.
7. Sutherell, C. L.; Ley, S. V. *Synthesis.* **2017**, *49*, (01), 135–144 . https://doi.org/10.1055/S-0035-1562792.
8. SHELXT: Sheldrick, G.M. *Acta Cryst.* **2015**, *A71*, 3-8.
9. SHELXL: Sheldrick, G.M. *Acta Cryst.* **2015**, *C71*, 3-8.
10. Parsons,S.; Flack, H. D; Wagner, T. *Acta Cryst.* **2013**, B69, 249-259.
